# Randomised controlled trial of fosfomycin in neonatal sepsis: pharmacokinetics and safety in relation to sodium overload

**DOI:** 10.1136/archdischild-2021-322483

**Published:** 2022-01-25

**Authors:** Christina W Obiero, Phoebe Williams, Sheila Murunga, Johnstone Thitiri, Raymond Omollo, Ann Sarah Walker, Thaddaeus Egondi, Borna Nyaoke, Erika Correia, Zoe Kane, Silke Gastine, Karin Kipper, Joseph F Standing, Sally Ellis, Mike Sharland, James Alexander Berkley, the NeoFosfo Study Group

**Affiliations:** 1 Clinical Research Deptartment, KEMRI-Wellcome Trust Research Programme, Kilifi, Kenya; 2 Department of Global Health, University of Amsterdam, Amsterdam, The Netherlands; 3 Nuffield Department of Medicine, University of Oxford, Oxford, UK; 4 Global Antibiotic Research and Development Partnership, Nairobi, Kenya; 5 MRC Clinical Trials Unit, University College London, London, UK; 6 Global Antibiotic Research and Development Partnership, Geneva, Switzerland; 7 Great Ormond Street Institute of Child Health, University College London, London, UK; 8 Institute of Chemistry, University of Tartu, Tartu, Estonia; 9 Analytical Services International (ASI) Ltd, St George’s – University of London, London, UK; 10 Paediatric Infectious Diseases Research Group, Institute for Infection and Immunity, University of London, London, UK; 11 The Childhood Acute Illness & Nutrition (CHAIN) Network, Nairobi, Kenya

**Keywords:** neonatology, sepsis, pharmacology

## Abstract

**Objective:**

To assess pharmacokinetics and changes to sodium levels in addition to adverse events (AEs) associated with fosfomycin among neonates with clinical sepsis.

**Design:**

A single-centre open-label randomised controlled trial.

**Setting:**

Kilifi County Hospital, Kenya.

**Patients:**

120 neonates aged ≤28 days admitted being treated with standard-of-care (SOC) antibiotics for sepsis: ampicillin and gentamicin between March 2018 and February 2019.

**Intervention:**

We randomly assigned half the participants to receive additional intravenous then oral fosfomycin at 100 mg/kg two times per day for up to 7 days (SOC-F) and followed up for 28 days.

**Main outcome(s) and measure(s):**

Serum sodium, AEs and fosfomycin pharmacokinetics.

**Results:**

61 and 59 infants aged 0–23 days were assigned to SOC-F and SOC, respectively. There was no evidence of impact of fosfomycin on serum sodium or gastrointestinal side effects. We observed 35 AEs among 25 SOC-F participants and 50 AEs among 34 SOC participants during 1560 and 1565 infant-days observation, respectively (2.2 vs 3.2 events/100 infant-days; incidence rate difference −0.95 events/100 infant-days (95% CI −2.1 to 0.20)). Four SOC-F and 3 SOC participants died. From 238 pharmacokinetic samples, modelling suggests an intravenous dose of 150 mg/kg two times per day is required for pharmacodynamic target attainment in most children, reduced to 100 mg/kg two times per day in neonates aged <7 days or weighing <1500 g.

**Conclusion and relevance:**

Fosfomycin offers potential as an affordable regimen with a simple dosing schedule for neonatal sepsis. Further research on its safety is needed in larger cohorts of hospitalised neonates, including very preterm neonates or those critically ill. Resistance suppression would only be achieved for the most sensitive of organisms so fosfomycin is recommended to be used in combination with another antimicrobial.

**Trial registration number:**

NCT03453177.

## Abstract (precis)

A trial of Fosfamycin in combination with standard antibiotic therapy in newborn sepsis which established the dose that is appropriate in preterm and term neonates.

**What is already known on this topic?**

- Antimicrobial resistance poses a threat to neonatal survival and there is an urgent need for affordable new treatment options.
- Intravenous fosfomycin presents a significant sodium load and oral fosfomycin preparations contain a large amount of fructose, but limited safety data exist in neonates.
- Paediatric and neonatal dosing recommendations for intravenous fosfomycin are divergent and there are no published oral dosing regimens.

**What this study adds?**

- Intravenous and oral fosfomycin had no evidence of impact on serum sodium or gastrointestinal side effects at 100 mg/kg two times per day, respectively.
- Intravenous fosfomycin 150 mg/kg two times per day is likely required for pharmacodynamic target attainment in most children, reduced to 100 mg/kg two times per day in neonates aged <7 days or weighing <1500 g.
- Fosfomycin has potential for affordable treatment of neonatal sepsis in combination with other antimicrobials while sparing carbapenems in the context of increasing antimicrobial resistance.

## Introduction

Antimicrobial resistance (AMR) disproportionally impacts populations in low-income and middle-income countries (LMICs). Reductions in mortality have been less in neonates than older children, and at least one-quarter of neonatal deaths are attributable to infection.^1^ AMR contributes to this burden, with multidrug-resistant (MDR) pathogens accounting for ~30% of global neonatal sepsis deaths.^2^

WHO recommends ampicillin, penicillin or cloxacillin (if *Staphylococcus aureus* infection is suspected) plus gentamicin (first-line), and third-generation cephalosporins (second-line) for empiric treatment of neonatal sepsis.^3^ With spread of extended spectrum β-lactamase (ESBL) and carbapenemase enzymes,^4^ clinical isolates are commonly reported non-susceptible to this regimen.^5^ Carbapenem-sparing is important in controlling MDR,^6^ and reintroduction of legacy antibiotics has been advocated to address the lack of new affordable antibiotics.^7^

Fosfomycin is an off-patent phosphonic acid derivative identified as ‘critically important’ by WHO.^8^ Fosfomycin is bactericidal^9^ and exhibits activity against Gram-positive and Gram-negative bacteria, including methicillin-resistant *S. aureus*, vancomycin-resistant *Enterococcus* spp, ESBL producers and may penetrate biofilms.^10^ Fosfomycin demonstrates in vitro synergy with aminoglycosides and carbapenems^11 12^ and is commonly used for MDR urinary tract infections in adults.^13^

Current paediatric intravenous fosfomycin dosing recommendations are divergent, ranging between 100 and 400 mg/kg/day, without published oral dosing regimens. Four neonatal studies estimate an elimination half-life of 2.4–7 hours following 25–50 mg/kg intravenously.^14 15^ Protein binding was minimal and maximum concentration was in-line with adult data.^16 17^ Bactericidal effects are thought to correlate with either time above the minimum inhibitory concentration (MIC)^16^ or area under the curve (AUC):MIC ratio.^18 19^

Case reports totalling 84 neonates treated with intravenous fosfomycin 120–200 mg/kg/day suggest it is well-tolerated.^20–24^ Toxicity among adults and older children appears low.^25^ However, parenteral fosfomycin contains 14.4 mmol/330 mg sodium per gram—a potential safety concern in neonates whose sodium reabsorption is inversely proportional to gestational age (GA).^26^ Furthermore, oral fosfomycin contains a high fructose load (~1600 mg/kg/day), which may predispose to gastrointestinal side effects and impact fluid balance.^27 28^

We aimed to assess pharmacokinetics (PK) and changes to sodium levels in addition to adverse events (AEs) associated with intravenous followed by oral fosfomycin in neonates with clinical sepsis.

## Methods

### Participants and study design

We conducted an open-label randomised controlled trial of standard-of-care (SOC) antibiotics alone, versus SOC plus intravenous then oral fosfomycin, in neonates with clinical sepsis at Kilifi County Hospital (KCH), Kenya.

### Screening and eligibility

**Figure 1:**
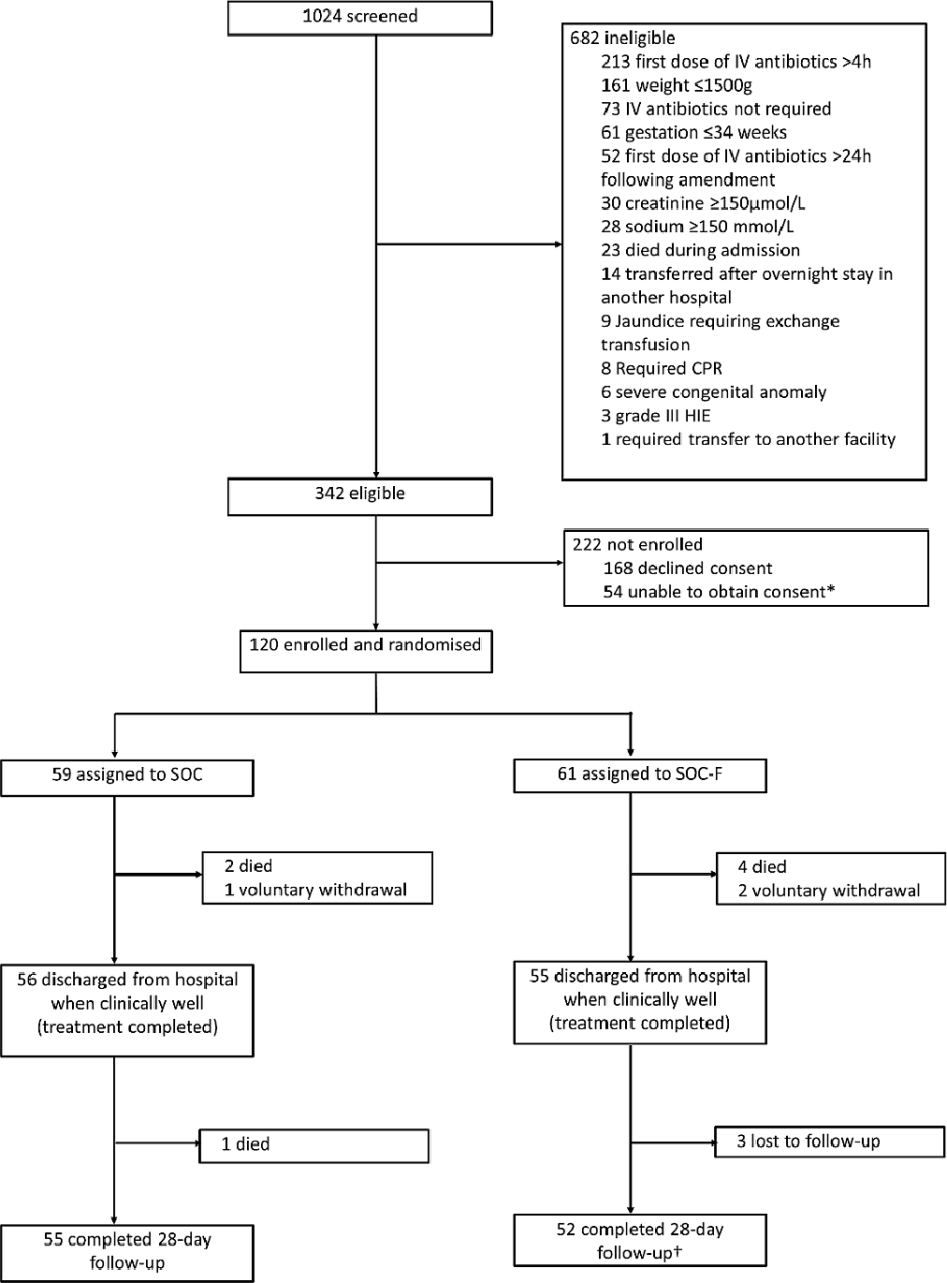
Trial flow chart. This original figure was created by CWO for this manuscript. CPR, cardiopulmonary resuscitation; HIE, hypoxic ischaemic encephalopathy; IV, intravenous; SOC, standard of care; SOC-F, standard of care plus fosfomycin. *Reasons include mother postcaesarean section (46) or seriously ill (6), absconded from hospital (3), discharged against advice (3), abandoned by mother (1) and already enrolled into another study (1). †One SOC-F participant died after completing follow-up (on day 106).

All neonates admitted to KCH were screened. Inclusion criteria were: age ≤28 days, weight >1500 g, gestation >34 weeks and meeting criteria for intravenous antibiotics per WHO^3^ and Kenyan^29^ guidelines. Neonates were excluded if requiring cardiopulmonary resuscitation, grade 3 hypoxic ischaemic encephalopathy,^30^ sodium ≥150 mmol/L, creatinine ≥150 µmol/L, jaundice requiring exchange transfusion, allergy or contraindication to fosfomycin, a specific indication for another antibiotic class, admitted from another hospital or not residing within Kilifi county (figure 1).

Participants were enrolled within 4 hours of the first dose of SOC antibiotics, until September 2018 when a protocol amendment extended this to within 24 hours to include overnight admissions.

### Enrolment and randomisation

A randomisation schedule with random block sizes was used to assign participants (1:1) to continue SOC antibiotics only or receive SOC plus (up to) 7 days of fosfomycin (SOC-F) (online supplemental figure S1). Concealment was by sequentially numbered opaque sealed envelopes.

10.1136/archdischild-2021-322483.supp2 Supplementary data

### Study treatment

SOC entailed ampicillin or cloxacillin (if staphylococcal infection was suspected) plus gentamicin as first-line antibiotics, or third-generation cephalosporins (eg, ceftriaxone) as second-line antibiotics according to WHO and Kenya paediatric guidelines.^3 29^ Participants randomised to SOC-F also received intravenous fosfomycin for at least 48 hours, switching to oral when tolerating feeds sufficiently to presume adequate absorption of oral medications. Fosfomycin (intravenous or oral) was administered for 7 days or until discharge, whichever occurred first. Fomicyt 40 mg/mL fosfomycin sodium solution for intravenous infusion (Infectopharm, Germany) and Fosfocina 250 mg/5 mL fosfomycin calcium suspension for oral administration (Laboratorios ERN, Spain) were given at 100 mg/kg/dose two times per day.

### Follow-up, safety monitoring and outcomes

Participants were followed-up for 28 days. All participants were cared for in the same high dependency unit to standardise AE monitoring. Complete blood count and biochemistry (including sodium) were done at admission, days 2 and 7, and were repeated if clinically indicated. AEs were coded according to MedDRA V.22.0. Severity was classified according to DAIDS V.2.1. AEs were followed up until clinical resolution or judged to be chronic and stable while receiving care. ‘Anticipated’ AEs were defined a priori as those expected to occur commonly in this population, including likely deteriorations of conditions present at birth (trial protocol in online supplemental file 1).

10.1136/archdischild-2021-322483.supp1 Supplementary data

### Pharmacokinetics

Patients allocated to SOC-F were randomly assigned to one early (5, 30 or 60 min) and one late (2, 4 or 8 hours) PK sample after both the first intravenous and first oral fosfomycin doses. A non-systematic fifth sample was collected for participants still hospitalised on day 7. Opportunistic cerebrospinal fluid (CSF) samples were collected from clinically indicated lumbar punctures (LP). Sample processing and fosfomycin measurement are described in online supplemental file 2.

### Statistical methods

We reviewed admission data between 2015 and 2016 and calculated a mean sodium of 139 mmol/L (SD 7.6, range 106–198) among 1785 neonates weighing >1500 g. Excluding 132 neonates who had serum sodium of >150 mmol/L (our exclusion criteria) resulted in a mean sodium of 137 mmol/L (SD 5.2) among the remaining 1653 neonates. A sample size of 45 per arm was subsequently calculated to ensure a 5 mmol/L difference in plasma sodium at day 2 could be determined with >85% power based on local prior sodium distribution data.

For PK, a sample size of 45 provided >85% power to estimate PK parameters for clearance, volume of distribution and bioavailability with 95% CIs with precision of ≥20% using simulation-estimation. For this, an adult disposition model, with age and size scaling to neonates with added first-order absorption and assumed bioavailability was used.^31^ To allow for missed samples, we aimed to recruit 60 neonates per arm.

Differences in baseline parameters were tested using χ^2^ test, Student’s t-test or Wilcoxon rank-sum test. Differences in sodium, potassium, creatinine and alanine aminotransferase at day 2 and 7 were tested using analysis of covariance adjusting for baseline values. For AEs, serious adverse events (SAEs) and adverse drug reactions, we estimated incidence rate ratios (IRR) and rate differences (IRD) between arms with two-sided exact CIs using STATA V.15.1 (StataCorp, College Station, Texas, USA).

Model-based estimation of PK parameters was undertaken using first-order conditional estimation with interaction in NONMEM V.7.4.^32^ Full details of PK model development and simulations are provided elsewhere.^32^

### Ethical review and oversight

DND*i/*GARDP undertook on-site monitoring and an independent Data Safety and Monitoring Board provided oversight.

## Results

### Enrolment

Between 19 March 2018 and 6 February 2019, 120 neonates (61 SOC-F, 59 SOC) were enrolled (figure 1), 42 (35%) before the protocol amendment. Median (IQR) age, weight and GA were 1 day (IQR 0–3), 2750 g (2370–3215) and 39 weeks (38–40), respectively. Baseline characteristics and laboratory parameters are presented in table 1 and online supplemental table S1.

**Table 1:** Baseline characteristics

	SOC (n=59)	SOC-F (n=61)	All (n=120)	P value (SOC vs SOC-F)
Age (days)	1 (0–4)	1 (0–3)	1 (0–3)	
Gestational age (weeks)	38 (37–40)	40 (38–40)	39 (38–40)	0.079
Sex				
Female	24 (41)	24 (39)	48 (40)	0.881
Male	35 (59)	37 (61)	72 (60)	
Anthropometry				
Weight (g)	2700 (2080–3200)	2800 (2500–3230)	2750 (2370–3215)	0.154
Head circumference (cm)	34.0 (32.5–36.0)	34.7 (33.6–36.0)	34.6 (33.0–36.0)	0.173
Length (cm)	48.0 (44.4–49.5)	48.0 (46.0–49.5)	48.0 (45.0–49.5)	0.371
Admitted from				
KCH maternity	24 (41)	28 (46)	52 (43)	0.846
Other health facility	20 (34)	19 (31)	39 (33)	
Home	15 (25)	14 (23)	29 (24)	
Clinical symptoms				
Fever	21 (36)	22 (36)	43 (36)	0.957
Difficulty in breathing	39 (66)	40 (66)	79 (66)	0.951
Difficulty feeding	10 (17)	11 (18)	21 (18)	0.876
Seizures	8 (14)	11 (18)	19 (16)	0.502
Vomiting	1 (1.7)	1 (1.6)	2 (1.7)	0.981
Clinical signs				
Axillary temperature (°C)	36.8 (36.3–37.4)	37 (35.7–37.6)	36.9 (35.9–37.5)	0.580
Heart rate (bpm)	147 (136–161)	147 (138–158)	147 (138–159)	0.471
Respiratory rate (bpm)	54 (45–68)	56 (48–68)	56 (48–68)	0.953
Oxygen saturation (%)	96 (86–97)	95 (88–98)	96 (88–98)	0.484
Capillary refill time ≥2 s	12 (20)	14 (23)	26 (22)	0.728
Respiratory distress*	43 (73)	37 (61)	80 (67)	0.156
Jaundice	6 (10)	11 (18)	17 (14)	0.217
Skin lesions†	4 (6.8)	3 (4.9)	7 (5.8)	0.664
Abdominal distension	5 (8.5)	1 (1.6)	6 (5.0)	0.086
Impaired consciousness‡	2 (3.4)	9 (15)	11 (9.2)	0.031
Abnormal posture	1 (1.7)	3 (4.9)	4 (3.3)	0.223
Abnormal tone	8 (14)	13 (21)	21 (18)	0.264
Bulging fontanel				
Agitated	9 (15)	11 (18)	20 (17)	0.683
Lethargic	10 (17)	17 (28)	27 (23)	0.152

Data are n (%) or median (q25–q75).

*Nasal flaring, lower chest wall indrawing and/or grunting.

†Pustules, vesicles, petechiae and/or cellulitis.

‡Responsive to pain only or unresponsive.

KCH, Kilifi County Hospital; SOC, standard of care; SOC-F, standard of care plus fosfomycin.

Two neonates had detected bacteraemia (online supplemental table S2). Two of 55 neonates who underwent an LP had laboratory-confirmed meningitis (*Streptococcus agalactiae* bacteraemia with CSF leucocytes ≥20 cells/µL (SOC-F); positive CSF antigen test for *Streptococcus pneumoniae* and CSF leucocytes ≥20 cells/µL (SOC)).

### Treatment fidelity and follow-up

One SOC-F neonate erroneously received only SOC antimicrobials and was excluded from PK analyses. Two SOC-F and one SOC neonate withdrew consent—data are included up to withdrawal. All except two SOC participants (cloxacillin plus gentamicin (n=1) and ceftriaxone (n=1)) received ampicillin plus gentamicin at admission. Online supplemental table S3 shows antibiotic combinations administered in participants who received antibiotics other than ampicillin plus gentamicin at admission or following change of treatment. Ten SOC-F participants switched to second-line therapy due to clinical deterioration or meningitis, five prior to the fourth PK sample (online supplemental table S3). Overall, 60 participants received at least one intravenous fosfomycin dose and 58 at least one oral dose.

Six (four SOC-F, two SOC) participants died in hospital (figure 1). One SOC participant died 3 days postdischarge (day 22). One SOC-F participant missed follow-up and was later found to have died on day 106 (outside the study follow-up period); data were included up to day 28. Three SOC-F infants were lost to follow-up. Total infant/days of observation were 1560 and 1565 for SOC-F and SOC, respectively, of which 422 and 314 were in hospital.

### Biochemical safety

On day 2, the mean (SD) plasma sodium values were 137 mmol/L (4.6) in SOC-F vs 136 mmol/L (3.7) in SOC participants; mean difference +0.7 mmol/L (95% CI −1.0 to +2.4). On day 7, mean (SD) sodium values were 136 mmol/L (4.2) vs 139 mmol/L (3.3); mean difference −2.9 mmol/L (95% CI −7.5 to +1.8) (table 2).

**Table 2:** Descriptive summary of blood chemistry parameters by randomised treatment arm

Parameter	Statistic	Day 0		Day 2		Day 7	
		SOC	SOC-F	SOC	SOC-F	SOC	SOC-F
		(n=59)	(n=61)	(n=59)	(n=61)	(n=6)	(n=7)
Sodium (mmol/L)	Range (min-max)	126–145	125–149	126–143	126–149	136–144.8	128–141
	Mean (SD)	135.4 (4.1)	136.4 (5.3)	135.7 (3.8)	136.6 (4.6)	138.6 (3.3)	135.7 (4.2)
	Median (IQR)	136 (132–138)	136 (133–140)	136 (133.5–138)	136 (134–140)	137.9 (136–139)	136 (134–139)
	n (missing)	59 (0)	61 (0)	48 (11)	54 (7)	6 (0)	7 (0)
Creatinine (μmol/L)	Range (min-max)	32–147	35–142	39–135	33–122	40–77	40–74
	Mean (SD)	92.3 (28)	88.5 (24.1)	73.7 (24.1)	72.2 (20)	59.2 (12.7)	62 (11.4)
	Median (IQR)	96.5 (70–113)	89 (74–109)	72 (54.5–87)	70 (57–83)	59.5 (53–66)	65 (57–72)
	n (missing)	58 (1)	61 (0)	52 (7)	55 (6)	6 (0)	7 (0)
Potassium (mmol/L)	Range (min-max)	2.9–6.2	2.7–6.2	2.8–5.7	2.3–4.8	2.5–4.9	2.9–5.2
	Mean (SD)	4.3 (0.6)	4.3 (0.7)	3.9 (0.7)	3.5 (0.7)	4.1 (0.9)	3.9 (0.9)
	Median (IQR)	4.3 (3.9–4.6)	4.2 (3.8–4.7)	3.9 (3.4–4.4)	3.5 (3–4)	4.3 (3.8–4.9)	4 (3–4.4)
	n (missing)	59 (0)	61 (0)	48 (11)	55 (6)	6 (0)	7 (0)
Alanine transaminase (U/L)	Range (min-max)	23–238	25–244	15–475	16–152	44–83	23–64
	Mean (SD)	90.6 (58.4)	81.8 (46.5)	73.1 (78.3)	59.9 (32.5)	64.8 (18.3)	44.7 (14.2)
	Median (IQR)	74 (54–99)	68 (45–115)	51 (38.5–70)	56.5 (35–77)	66 (49.5–80)	46.5 (35–53)
	n (missing)	37 (22)	46 (15)	48 (11)	50 (11)	4 (2)	6 (1)

n, number; SOC, standard of care; SOC-F, standard of care plus fosfomycin.

On day 2, mean (SD) potassium concentration was marginally (yet not clinically significantly) lower in SOC-F than SOC infants: 3.5 mmol/L (0.7) vs 3.9 mmol/L (0.7), difference −0.4 mmol/L (95% CI −0.7 to −0.1). There was no evidence of difference between arms in other laboratory parameters (table 2).

### Adverse events

We observed 35 AEs in 25 SOC-F participants and 50 AEs in 34 SOC participants; 2.2 events/100 infant-days and 3.2 events/100 infant-days, respectively: IRR 0.7 (95% CI 0.4 to 1.1), IRD −0.9 events/100 infant-days (95% CI −2.1 to +0.2, p=0.11).

Twelve SAEs occurred among 11 SOC-F participants and 14 SAEs among 12 SOC participants (0.8 events/100 infant-days in SOC vs 1.0 events/100 infant-days; IRR 0.8 (95% CI 0.4 to 1.8), IRD −0.2 events/100 infant-days (95% CI −0.9 to +0.5, p=0.59). Hypoglycaemia was the most common AE (five SOC-F and six SOC); four cases in each arm were grade 3 or 4 (online supplemental table S4). Three SOC-F and four SOC participants had moderate or severe thrombocytopenia and were well at day 28 without platelet transfusion. AEs classified as ‘anticipated’ occurred in 13 SOC-F and 13 SOC participants (online supplemental table S5). Three SOC participants were re-admitted to hospital (pneumonia (n=2) and febrile illness of unknown origin (n=1)); all were discharged home alive. One SOC-F participant had a mild perineal rash and another SOC-F participant experienced moderate diarrhoea 13 days postdischarge; both resolved without sequelae. Excluding mortality, 50 AEs resolved while 27 were either resolving, had not changed or had resolved with sequelae (online supplemental table S6). No AEs were related to study medication.

### Pharmacokinetics

Sixty participants had at least one intravenous PK sample collected. Fifty-five participants contributed complete sets of four samples, and five participants had partial sets. Six participants had a sample collected on day 7. Overall, 238 plasma (119 for intravenous and 119 for oral fosfomycin) and 15 CSF samples were analysed. No sample had fosfomycin levels below the limit of quantification.^32^

Population PK model development and simulation results are described in detail elsewhere.^32^ Briefly, a two-compartment PK disposition model with an additional CSF compartment provided a good fit to the data, with clearance and volume at steady-state for a typical participant (weight (WT) 2805 g, postnatal age (PNA) 1 day, postmenstrual age (PMA) 40 weeks) being 0.14 L/hour (0.05 L/hour/kg) and 1.07 L (0.38 L/kg), respectively. In addition to fixed allometric and expected PMA maturation based on renal function,^31^ PNA was associated with increasing clearance over the first week of life. The model-based population estimate of oral bioavailability was 0.48 (95% CI 0.35 to 0.78) and CSF/plasma ratio was 0.32 (95% CI 0.27 to 0.41).

**Figure 2:**
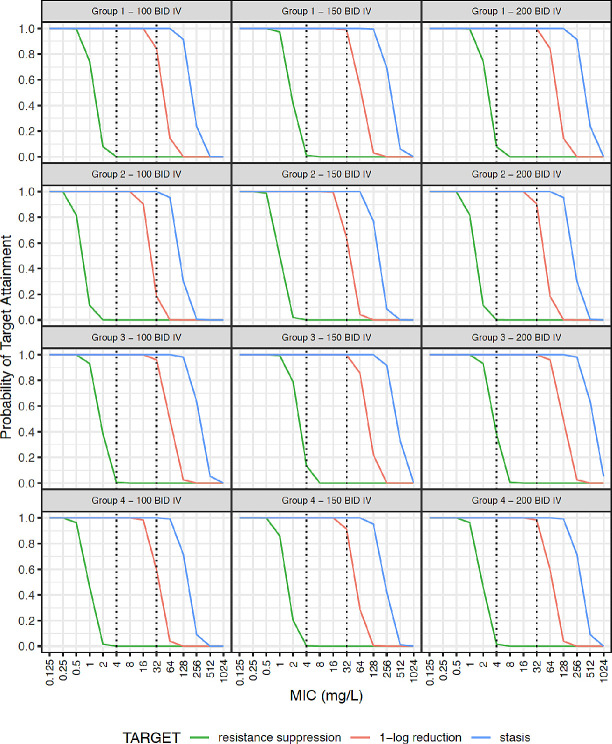
Probability target attainment for intravenous fosfomycin dosing. Neonatal subpopulations. Group 1: WT >1.5 kg +PNA ≤7 days (n=4391), group 2: WT >1.5 kg +PNA >7 days (n=2798), group 3: WT ≤1.5 kg +PNA ≤7 days (n=1534), group 4: WT ≤1.5 kg +PNA >7 days (n=1277). Groups 1 and 2 represent patients similar to those fitting our inclusion criteria. Groups 3 and 4 represent an extrapolation to preterm neonates that were not studied in our population. This original figure was created by ZK for this manuscript. BID, two times per day; IV, intravenous; MIC, minimum inhibitory concentration; PNA, postnatal age; WT, weight.

**Figure 3:**
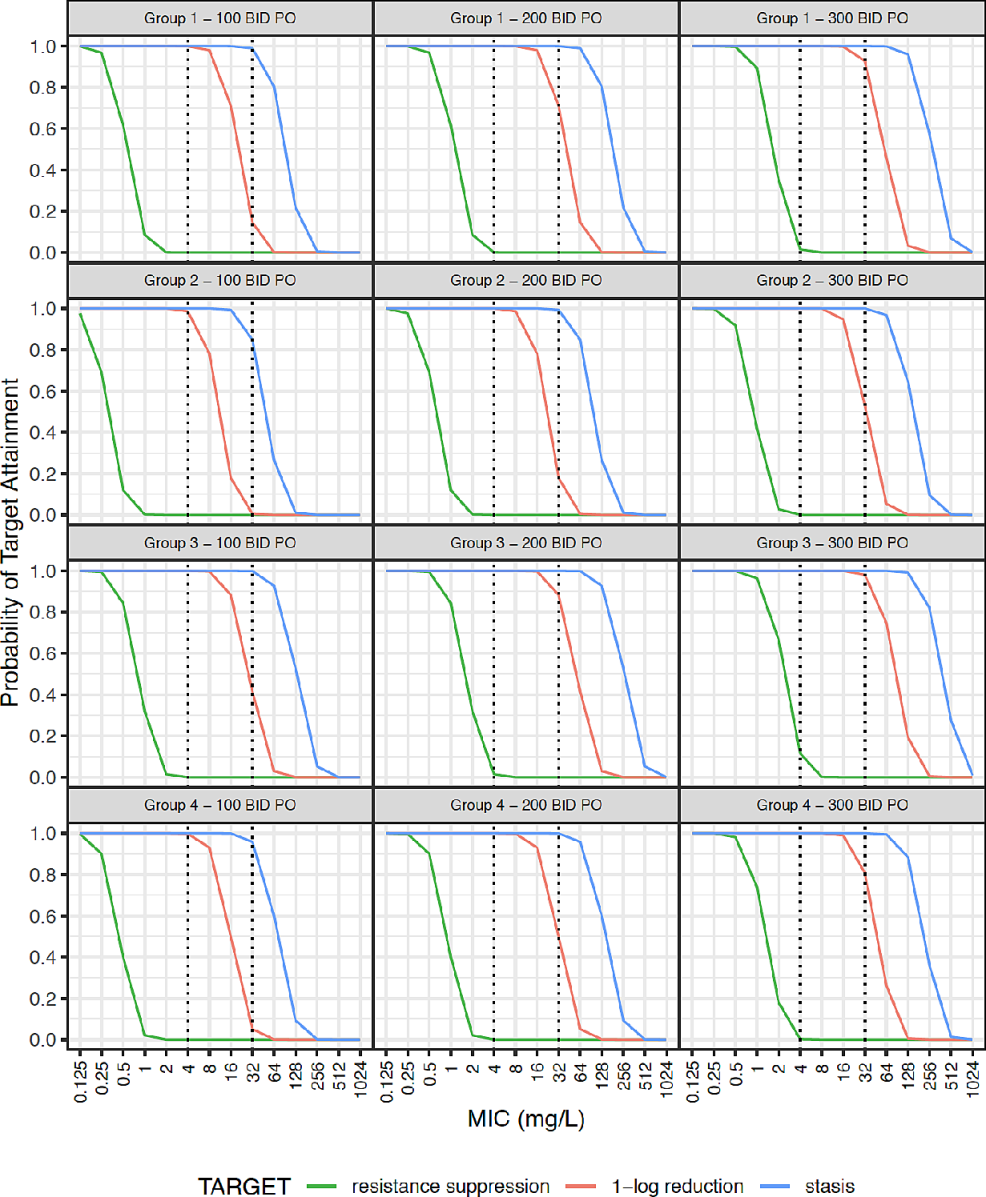
Probability target attainment for oral fosfomycin dosing. Neonatal subpopulations. Group 1: WT >1.5 kg +PNA ≤7 days (n=4391), group 2: WT >1.5 kg +PNA >7 days (n=2798), group 3: WT ≤1.5 kg +PNA ≤7 days (n=1534), group 4: WT ≤1.5 kg +PNA >7 days (n=1277). Groups 1 and 2 represent patients similar to those fitting our inclusion criteria. Groups 3 and 4 represent an extrapolation to preterm neonates using external data that were not studied in our population. This original figure was created by ZK for this manuscript. BID, two times per day; MIC, minimum inhibitory concentration; PNA, postnatal age; PO, oral; WT, weight.

Simulated steady-state plasma concentration-time curves are illustrated in online supplemental figure S2. Probability of target attainment (PTA) for AUC:MIC thresholds for bacteriostasis, 1-log kill and resistance suppression is given in figures 2 and 3 for the studied population (weight >1500 g), and extrapolated using data from smaller neonates. Given the rapid increase in clearance over the first week of life, simulations were further stratified by PNA (online supplemental table S7).

Resistance suppression could not be consistently achieved with any simulated dosing regimens for organisms with MIC >0.5 mg/L (figures 2 and 3). For 100 mg/kg two times per day intravenously, bacteriostasis could be achieved with 100% PTA for an MIC of 32 mg/L in all four simulated strata (figure 2). Regarding 1-log kill, PTA for 100 mg/kg two times per day intravenously for an MIC of 32 mg/L was 0.84 and 0.96 for groups 1 and 3 with PNA ≤7 days, but PTA was lower at 0.19 and 0.60 for groups 2 and 4 with PNA >7 days. At 150 and 200 mg/kg two times per day intravenously, PTA for 1-log kill was 0.64 and 0.90 in group 2, and 0.91 and 0.98 in group 4, respectively.

Oral dosing with 100 mg/kg two times per day in groups 2 and 4 yielded PTA values for bacteriostasis of 0.85 and 0.96, respectively (figure 3), and PTAs for groups 1–4 were 0.15, 0.004, 0.41 and 0.05, respectively for 1-log kill at an MIC of 32 mg/L.

## Discussion

We provide evidence for the use of fosfomycin in infants at 100 mg/kg/dose two times per day, without evidence of plasma sodium disturbance (intravenous) or osmotic diarrhoea (oral) when compared with SOC. Our primary safety objective, to detect differences in plasma sodium levels between the two treatment arms on day 2, was adequately powered. Although our sample size was too small to determine group differences for other safety events, all neonates were closely monitored, and events reported contribute towards evidence supporting the potential use of fosfomycin as an alternative empiric treatment for sepsis in this vulnerable group. However, confirmation of these results in larger and sicker cohorts will be important.

We aimed to enrol neonates aged ≤28 days and did not selectively include suspected early onset sepsis. However, 86% neonates were hospitalised within the first week of life, confirming the high burden of early neonatal morbidity reported in similar LMICs.^33–36^ High levels of resistance of pathogens causing early onset and late-onset sepsis (including ESBL *Escherichia coli* and *Klebsiella pneumoniae*) to empiric antimicrobials have been observed,^37–39^ potentially acquired in the maternity department. Broad-spectrum antimicrobial coverage that includes fosfomycin as first-line treatment in such settings may improve outcomes and spare the use of carbapenems.

In common with many antimicrobials,^40^ PNA was a key covariate in describing fosfomycin clearance. This effect was distinct from GA and weight and represents rapid glomerular filtration maturation postnatally. Locally, 90% of invasive Enterobacterales had fosfomycin MIC ≤32 µg/mL^15^ and for neonates aged >7 days it is likely that >100 mg/kg/dose intravenously is required for bactericidal activity (figure 2). For a 32 µg/mL target, 150 mg/kg two times per day is suggested for intravenous treatment if PNA >7 days. Once stabilised and if there is a requirement to move to oral fosfomycin, doses can be selected with consideration of a neonate’s WT, PMA, PNA and the likely pathogen MIC but should take into account the bioavailability reported here. Studies are needed to further assess the safety profile and efficacy of this higher dose recommended by our PK model.

Current guidance on neonatal parenteral fluid and electrolyte intake suggests limiting sodium supplementation to 2–3 mmol/kg/day with PNA >3 days, with preterm neonates requiring up to 5 mmol/kg/day.^41^ The studied fosfomycin intravenous formulation, at 100 mg/kg/dose two times per day, provides 2.8 mmol/kg/day sodium. SOC-F neonates achieved median sodium levels <140 mmol/L with only one neonate exceeding 145 mmol/L (149 mmol/L). Sodium intake using this fosfomycin formulation at 150 mg/kg two times per day is calculated at 4.2 mmol/kg/day. Thus, higher doses as per revised European Medicines Agency recommendations^42^ will require monitoring electrolytes to confirm safety. In addition, studies are needed in neonates with shock or renal failure who need close monitoring of electrolytes and fluid balance and will likely require dose adjustment.

Since resistance suppression could only be achieved for the most sensitive of organisms, and fosfomycin-inactivating enzymes may exist in transferrable plasmids,^43^ fosfomycin is recommended to be used in combination with another antibiotic. The potential utility of fosfomycin plus amikacin for neonatal sepsis was recently studied by assessing in vitro activity and pharmacodynamic interactions using checkerboard assays and a 16-arm dose-ranged hollow-fibre infection model.^44^ This combination had enhanced bactericidal activity, prevented the emergence of resistance, and achieved sterility with lower combination exposures, compared with monotherapy with either antibiotic. This study concluded that fosfomycin plus amikacin combination is suitable for further clinical assessment. Simulation-based PK/pharmacodynamic assessments of ampicillin and gentamicin on 373 residual samples collected from 59 SOC-F participants suggested good Gram-positive cover (MIC ≤0.25 mg/L) but poor coverage against Enterobacterales (MIC ≤2 mg/L), underscoring the need for alternative antibiotic combinations in settings with high resistant rates. Although analysis of fosfomycin interaction with ampicillin, gentamicin or ceftriaxone was not done in this study, previous studies have shown that it has synergistic activity with β-lactams, aminoglycosides and cephalosporins.^45^

Trials evaluating fosfomycin combinations in neonatal sepsis are urgently needed^46^ and our data provide the basis on which to evaluate efficacy within a combination in multiple settings compared with current SOC, either empirically or to treat microbiologically confirmed MDR infections. We are planning a multisite randomised clinical trial to assess novel antimicrobial combinations (including fosfomycin) for optimal treatment of sepsis in settings with high AMR rates and variable SOC antimicrobial choices.^47^ This trial will be preceded by a run-in confirmatory PK study of fosfomycin at the higher dose identified in the current study and will generate further data on fosfomycin safety in a large population of neonates at moderate to high risk of mortality across different LMIC settings. Robust evidence of sepsis epidemiology and management in infants aged <60 days from a recently concluded observational study (NCT03721302) is contributing towards the design of this trial.

Limitations include single-centre recruitment and exclusion of the sickest neonates at enrolment, which was judged important given the very limited prior information. Our narrow eligibility criteria excluded neonates at highest risk of poor outcomes, including very preterm neonates or those critically ill or with conditions likely to cause hypernatraemia such as severe hypoxic ischaemic encephalopathy. Future trials need to include these vulnerable groups that may benefit most from optimal antibiotic treatment.

Our sample size was not intended to determine antimicrobial efficacy or comprehensively establish safety. Enrolment rate increased (42 enrolled/519 screened vs 79/505) after extension of recruitment window from 4 to 24 hours, based on guidelines on clinical evaluation of antimicrobial agents for AMR.^48^ We believe that this did not impact our results. Our study highlights challenges faced by researchers conducting early phase clinical trials in resource-limited settings including difficulties in obtaining informed consent from parents/guardians of vulnerable neonates. We implemented strategies to optimise consent such as ensuring that key decision makers within each family were involved during the process. The small CSF dataset provides evidence of appreciable concentrations in CSF; however, further data are required for firm dosing recommendations for meningitis.

Strengths of our trial include a low loss to follow-up, standardised observational data, a high ascertainment of PK samples and robust timing and dosing information—a logistically challenging exercise in neonates in any setting.^49^ Total observation days for neonates in both treatment arms were similar and sufficient number of neonates with available day 2 plasma sodium samples and complete sets of four PK samples contributed to this analysis, despite unbalanced losses due to consent withdrawals, loss to follow-up or deaths.

Increasing AMR in a population who may die rapidly due to inadequate antimicrobial coverage is concerning given limited new antibiotics in the pipeline. Fosfomycin offers significant potential as part of a safe, easily administered and affordable regimen.

10.1136/archdischild-2021-322483.supp3 Supplementary data

## Data Availability

Data are available on reasonable request. Trial datasets are deposited at https://dataverse.harvard.edu/dataverse/kwtrp and are available on request through the KEMRI/Wellcome Trust Research Programme Data Governance Committee dgc@kemri-wellcome.org.
