# Supplementary material for: Randomised controlled trial of fosfomycin in neonatal sepsis: pharmacokinetics and safety in relation to sodium overload
Source: Arch Dis Child. Author manuscript; Available in PMC 2023 Feb 3. (PMC9411916; doi:10.1136/archdischild-2021-322483)
Supplement: Supplementary Information [file EMS152547-supplement-Supplementary_Information.pdf]

| 2 DNDi AFRICA Drugs for Neglected Diseases *initiative* | Statistical Analysis Plan (SAP) | NEO_FOSFO_SAP_V2 | 22-Feb-2019 |
|---|---|---|---|

# Intravenous and Oral Fosfomycin in Hospitalised Neonates with Clinical Sepsis: An Open-label, Safety and Pharmacokinetic study (neoFosfo)

## STATISTICAL ANALYSIS PLAN

## (SAP)



Obiero CW, *et al. Arch Dis Child* 2022;0:1–9. doi: 10.1136/archdischild-2021-322483

**SIGNATURES:**

**Signature** ____________________ **Date (dd/mmm/yyyy)** ____________

**Name** Dr. Raymond Omollo, Head of Data Centre & Statistician

**Institution** Drugs for Neglected Diseases *initiative*

**Address** P.O. Box 20778-00202 Nairobi, Kenya

**Signature** ____________________ **Date (dd/mmm/yyyy)** ____________

**Name** Prof. Sarah Walker, Statistician

**Institution** University College, London, UK

**Address**

**Signature** ____________________ **Date (dd/mmm/yyyy)** ____________

**Name** Erika Correia, Clinical Research Associate

**Institution** GARDP

**Address** Chemin Louis Dunant, 15, 1202 Geneva, Switzerland

**Signature** ____________________ **Date (dd/mmm/yyyy)** ____________

**Name** Dr. Borna Nyaoke, Clinical Trial Manager

**Institution** GARDP

**Address** P.O. Box 20778-00202 Nairobi, Kenya

**Signature** __________________ **Date (dd/mmm/yyyy)** ____________

**Name** Sally Ellis, Project Leader

**Institution** GARDP

**Address** Chemin Louis Dunant, 15, 1202 Geneva, Switzerland

**Signature** ____________________ **Date (dd/mmm/yyyy)** ____________

**Name** Prof James A Berkley, PI

**Institution** KEMRI/Wellcome Trust Research Programme

**Address** P.O. Box 230-80108 Kilifi, Kenya

# Table of CONTENTS

Table of CONTENTS .......... 3
List of Tables .......... 5
List of Figures .......... 6
Abbreviations .......... 7
1. Scope .......... 8
2. Protocol Summary .......... 9
3. Trial Objectives and endpoints .......... 10
3.1. Trial Objectives .......... 10
3.1.1. General Objective .......... 10
3.1.2. Specific Objectives .......... 10
3.2. Trial Endpoints .......... 10
3.2.1. Primary endpoint .......... 10
3.2.2. Secondary endpoints .......... 10
4. Trial Design .......... 10
4.1. Study design .......... 10
4.2. Study duration .......... 11
4.3. Study Population .......... 11
4.3.1. Selection of Subjects .......... 11
4.3.2. Inclusion criteria .......... 11
4.3.3. Exclusion criteria .......... 11
4.4. Sample Size Determination .......... 12
5. Study Assessments .......... 12
5.1. Schedule of events .......... 12
5.2. Assessment of Safety .......... 14
5.2.1. Safety Reporting .......... 14
5.2.2. Clinical Laboratory assessments .......... 14
5.2.3. Anticipated Events in neonatal setting .......... 14
6. Planned Analysis .......... 15
6.1. Interim Analysis .......... 15
6.2. Final Analysis .......... 15
7. Statistical Methods .......... 16
7.1 Data Transformation .......... 16

**7.2 Baseline Data** ... 16
**7.3 Analyses set** ... 16
***7.4 Safety Analysis*** ... 16
***7.5 Biological Parameters*** ... 17
*7.5.1 Shift tables* ... 17
*7.5.2 Graphical representations* ... 17
***7.6 Software*** ... 17
***8. Results*** ... 18
***8.1. Trial Profile*** ... 18
***8.2. Patient Characteristics and baseline comparisons*** ... 18
***8.3. Exploratory Analysis*** ... 23
***8.3.1. Graphical representations*** ... 23
***8.3.2. Change in Lab values*** ... 24
***8.5. Safety Analysis: Adverse Events*** ... 26
***References*** ... 39

## List of Tables

***Table 1: Anticipated Events in Neonatal Setting*** ..... 15
***Table 2: Baseline demographic characteristics by the treatment group*** ..... 19
***Table 3: Baseline anthropometric and vital signs by the treatment group*** ..... 19
***Table 4: Baseline clinical symptoms by the treatment group*** ..... 20
***Table 5: Past medical history and concomitant illness by the treatment group*** ..... 20
***Table 6: Baseline summary of haematological parameters by treatment group*** ..... 20
***Table 7: Baseline summary of blood chemistry parameters by treatment group*** ..... 21
***Table 8: Baseline summary of Blood Gas parameters by treatment group*** ..... 23
***Table 9: Plasma Sodium Concentrations*** ..... 25
***Table 10: Creatinine*** ..... 25
***Table 11: Potassium Concentrations*** ..... 25
***Table 12: ALT*** ..... 25
***Table 13: Number of patients experiencing adverse events*** ..... 26
***Table 14. Serious adverse events listing*** ..... 27
***Table 15. Treatment discontinuation: AE and other reasons*** ..... 27
***Table 16. Serious and non-serious AEs by relationship to study medication*** ..... 27
***Table 17. Serious and non-serious AEs by severity*** ..... 27
***Table 18: Anticipated events by the treatment group*** ..... 28
***Table 19: Shift table for Haemoglobin*** ..... 29
***Table 20: Shift table for White Blood Cells*** ..... 30
***Table 21: Shift table for Neutrophils*** ..... 31
***Table 22: Shift table for Lymphocytes*** ..... 32
***Table 23: Shift table for Platelets*** ..... 33
***Table 24: Shift table for Sodium*** ..... 34
***Table 25: Shift table for SGOT/AST*** ..... 35
***Table 26: Shift table for Alkaline Phosphatase*** ..... 36
***Table 27: Shift table for Creatinine*** ..... 37

# List of Figures

**Figure 1: Schedule of Events** ........ 13
**Figure 2: Participant Flow** ........ 18
**Figure 3:Time trajectories for heart rate (bpm)** ........ 23
**Figure 4: Box plot for heart rate (bpm)** ........ 23
**Figure 5:Time trajectories for respiratory rate (bpm)** ........ 23
**Figure 6: Box plot for respiratory rate (bpm)** ........ 23
**Figure 7:Time trajectories for temperature (°c)** ........ 23
**Figure 8: Box plot for temperature (°c)** ........ 24
**Figure 9:Time trajectories for $SaO_2$ (%)** ........ 24
**Figure 10: Box plot for $SaO_2$ (%)** ........ 24
**Figure 11:Time trajectories for White cell count ($x10^3$/μL)** ........ 24
**Figure 12: Box plot for White cell count ($x10^3$/μL)** ........ 24
**Figure 13: Scatter plot for White cell count ($x10^3$/μL)** ........ 24
**Figure 14: Time trajectories for SGPT/ALT (U/L)** ........ 24
**Figure 15: Box plot for SGPT/ALT (U/L)** ........ 24
**Figure 16: Scatter plot for SGPT/ALT (U/L)** ........ 24
**Figure 17: Time trajectories for Creatinine (μmol/L)** ........ 24
**Figure 18: Box plot for Creatinine (μmol/L)** ........ 24
**Figure 19: Scatter plot for Creatinine (μmol/L)** ........ 24
**Figure 20. Time trajectories for Sodium (mmol/L)** ........ 24
**Figure 21: Box plot for Sodium (mmol/L)** ........ 24
**Figure 22: Scatter plot for Sodium (mmol/L)** ........ 24
**Figure 23. Time trajectories for Potassium (mmol/L)** ........ 24
**Figure 24: Box plot for Potassium (mmol/L)** ........ 24
**Figure 25: Scatter plot for Potassium (mmol/L)** ........ 24



Obiero CW, *et al. Arch Dis Child* 2022;0:1–9. doi: 10.1136/archdischild-2021-322483

## Abbreviations

AE : Adverse Event
ALT : Alanine Aminotransferase
AMR : Antimicrobial Resistance
ANOVA : Analysis of Variance
AST : Aspartate Aminotransferase
BW : Birth Weight
CBC : Complete Blood Count
CGMR-C : Centre for Geographic Medicine Research-Coast
CI : Confidence Interval
CNS : Central Nervous System
CONSORT : Consolidated Standards of Reporting Trials
CRE : Creatinine
CRF : Case Report Form
DAIDS : Division of AIDS
EMLc : Essential Medicines List for Children
GNB : Gram Negative Bacteria
Hb : Haemoglobin
HIE : Hypoxic Ischaemic Encephalopathy
IQR : Inter-Quartile Range
IV : Intravenous injection
KEMRI : Kenya Medical Research Institute
LMICs : Low and Middle-Income Countries
MCV : Mean Corpuscular Volume
MDR : Multi Drug Resistant
MedDRa : Medical Dictionary for Regulatory Activities
$PCO_2$ : Partial Pressure of Carbon Dioxide
PK : Pharmacokinetic
$PO_2$ : Partial Pressure of Oxygen
SAE : Serious Adverse Event
$SaO_2$ : Oxygen Saturation
SAP : Statistical Analysis Plan
SAR : Statistical Analysis Report
SD : Standard Deviation
SOC : Standard of Care
TBIL : Total Bilirubin
WBC : White Blood Cells
WHO : World Health Organization

# 1. Scope

This Statistical Analysis Plan (SAP) provides a description of the planned analyses and reporting for an open-label trial on safety and Pharmacokinetics of fosfomycin in Hospitalised Neonates with Clinical Sepsis in Kilifi, Kenya (*protocol v2.0 dated 13$^{th}$ April 2018: Safety and Pharmacokinetics of Fosfomycin in Hospitalised Neonates with Clinical Sepsis*). The main changes described in protocol amendment version 2.0 include the following:

- Allow flexibility in the sample size i.e. approximately 120 babies will be enrolled
- Collect information on the tolerability of oral Fosfomycin
- Change entry criteria from 4 hours after initiating ampicillin and gentamicin to 24 hours after initiation of ampicillin and gentamicin

Other changes were procedural clarifications or corrections of inconsistencies identified in the first version of the protocol.

Since the primary objective of the trial is on the PK of fosfomycin, this SAP mainly focuses on the analyses of secondary objectives i.e.:

a) Difference in mean plasma sodium concentrations at 48 hours and at day 7 or discharge date between the two arms and
b) Difference in the rate of adverse events from enrolment to day 28 between the two arms.

The analysis plan for the PK and antimicrobial resistance (AMR) susceptibility sub-study of this trial will be reported separately.

Any additional or unplanned analyses not specified in this SAP will be clearly identified as such in the Statistical Analysis Report (SAR) and any other manuscripts for publication produced from the trial.

The following documents have also been considered in the process of developing this SAP:

- Clinical Research Protocol for the trial
- Case Report Forms (CRFs) for the trial

The details on the conduct of this trial, the operational aspects of clinical assessments and timing for patients in this trial can also be found in the Clinical Research Protocol.

Any changes to this SAP will be approved by those listed on the signature page.

## 2. Protocol Summary

Antimicrobial resistance (AMR) has become a major issue in global health. Despite progress in the reduction of under 5 mortality rates in recent decades, the proportion of neonatal deaths occurring within this age group has increased, with almost one quarter of all neonatal deaths occurring due to serious bacterial infections. Common bacteria causing neonatal sepsis are now exhibiting widespread resistance to several classes of antibiotics. There is an urgent need to discover new, effective treatments and re-evaluate existing therapeutic agents to treat infections potentially caused by multi-drug resistant (MDR) pathogens. Gram-negative bacteria (GNB) predominate as the cause of neonatal sepsis and are increasingly associated with high rates of resistance to the currently recommended WHO empirical therapy regimen of ampicillin/penicillin and gentamicin. There is therefore a need to develop an updated empiric regimen with improved efficacy in the context of increasing MDR sepsis in neonates. New antimicrobials under development will be expensive once licenced, and there are currently no planned trials to assess their efficacy in neonates in low- and middle-income countries (LMICs).

One potential strategy is utilising an existing off-patent (and therefore affordable) antibiotic available in intravenous and oral formulations – fosfomycin. Fosfomycin has a wide spectrum of activity against Gram-positive and Gram-negative bacteria causing neonatal sepsis. It is mainly used for resistant urinary tract infections in adults but has licenced neonatal and paediatric doses in Europe (though dosing regimens vary between countries). Both oral and IV formulations are available. A large clinical trial to assess the efficacy of a fosfomycin plus an aminoglycoside combination (compared to the current WHO recommended ampicillin and gentamicin) is anticipated, including sites in Kenya. The ultimate aim is for fosfomycin to be included in the WHO essential medicines list for children (EMLc) and be available for use in developing countries, where rates of resistance to ampicillin and gentamicin have been estimated at over 40%. The first steps before this trial are to clarify the pharmacokinetics (PK) and safety profile of fosfomycin in neonates, as well as generate further information regarding local patterns of bacterial susceptibility to fosfomycin. The aim of this study is to fulfil both these steps. Fosfomycin (IV and oral) PK will be investigated among 60 neonates admitted to hospital provided with standard of care and fosfomycin. They will also be monitored to compare adverse events with 60 other neonates receiving standard of care only (without PK sampling).

In addition to this trial, the laboratory at KEMRI CGMR-C, previously archived bacterial isolates have been tested for their sensitivity to fosfomycin; and are collecting data and samples from trial participants to determine the faecal carriage of antimicrobial resistance, including to fosfomycin, at admission and discharge.

# 3. Trial Objectives and endpoints

## 3.1. *Trial Objectives*

### 3.1.1. *General Objective*

To understand the fosfomycin pharmacokinetics, safety and antimicrobial susceptibility of local invasive bacterial species.

### 3.1.2. *Specific Objectives*

The specific objectives of the trial are to;

a) Estimate the PK disposition parameters of IV and PO fosfomycin in neonates
b) Assess the safety of fosfomycin, particularly with regards to possible elevation of sodium after 48 hours of IV fosfomycin administration in neonates
c) Estimate the oral bioavailability of fosfomycin in neonates
d) Generate preliminary data on the safety of oral fosfomycin in neonates
e) Generate a recommended dosing schedule for future IV and PO fosfomycin trials

**Note:**

Since the primary objective of the trial is on the PK of fosfomycin, this SAP focuses on the analyses of secondary objectives i.e.:

a) Difference in mean plasma sodium concentrations at 48 hours and at day 7 or discharge date between the two arms and
b) Difference in the rate of adverse events from enrolment to day 28 between the two arms

## 3.2. *Trial Endpoints*

### 3.2.1. *Primary endpoint*

Estimation of the pharmacokinetic disposition and absorption parameters of IV and oral fosfomycin in neonates with clinical sepsis with sufficient precision such that a dose schedule can be recommended for a future efficacy trial.

### 3.2.2. *Secondary endpoints*

a) Plasma sodium concentrations taken at 48 hours and at 7 days or at discharge.
b) Adverse events (any grade) experience during 28 days after enrolment in the study

# 4. Trial Design

## 4.1. *Study design*

This is an open label, safety and pharmacokinetic study among neonates admitted to a rural hospital in Kenya and eligible for IV antibiotics under current national guidelines. Approximately 120 patients will be randomized 1:1 to standard-of-care antibiotics plus a 7-day course of fosfomycin (up to n=60 to achieve at least 45

complete PK sample sets); or standard of care (n=60) antibiotics only (ampicillin 50mg/kg twice daily and gentamicin (3mg/kg for babies<2kg or 5mg/kg for babies>2kg) once daily for 7 days, as per Kenyan treatment guidelines).

## 4.2. Study duration

The study duration for each participant is 28 days from enrolment.

## 4.3. Study Population

### 4.3.1. Selection of Subjects

The following eligibility criteria were designed to select subjects for whom the protocol treatment is considered appropriate.

### 4.3.2. Inclusion criteria

Subjects must meet **all** the following three inclusion criteria to be eligible for enrolment into the study:

a) Patients aged 0 to 28 days inclusive.
b) Weight >1500g
c) Born (an estimated)>34 weeks gestation (calculated as per the Ballard Maturational Assessment)
d) Admitted to hospital and eligible to receive IV antibiotics, according to national guidelines

### 4.3.3. Exclusion criteria

The presence of **any** of the following will exclude a subject from study enrolment:

a) Baseline sodium level ≥150mmol/L
b) Baseline creatinine ≥150micromol/L
c) Presenting with severe (grade 3) Hypoxic Ischaemic Encephalopathy (HIE), defined as per Sarnat and Sarnat[44] as a stuporous, flaccid infant (with or without seizure activity) with suppressed brainstem and autonomic functions and absent reflexes
d) Requiring cardiopulmonary resuscitation on admission
e) Jaundice requiring exchange transfusion
f) Admitted as a transfer after an overnight inpatient stay at another hospital
g) Known allergy or contraindication to fosfomycin
h) A specific clinical indication for another class of antibiotic (other than the nationally recommended standard of care)
i) More than 24 hours after initiating ampicillin plus gentamicin (one dose of gentamicin), which allows for administration of these first line antibiotics not to be delayed by study procedures
j) Concurrent participation in another clinical trial
k) Attending clinician’s judgement that the child is so severely ill that adequate communication about the study with the parent or legal guardian is not possible
l) Not planning to remain resident in the county for the next 28 days
m) Lack of consent

### 4.4. Sample Size Determination

Sample size has been calculated to ensure that the following PK parameters can be estimated with sufficient precision such that a dose schedule can be recommended for a future efficacy trial:

- Clearance (CL)
- Central volume (V)
- Oral bioavailability (F)

Precision limits were set to 20%, and the power to estimate parameters with 95% confidence intervals within these limits was assessed by simulation-estimation. The simulation model consisted of an adult disposition model, with age and size scaling down to neonates, with added first-order absorption and assumed bioavailability. Six sampling time points were chosen to cover the dose intervals (3 early, 3 late) and the simulated population was randomly assigned age and weight combinations across the range expected for neonates. Parallel and cross over (IV/oral) designs were considered, with a range of 2-4 samples per patient. Power for sample size was greatest for the cross-over design. For the cross-over design, a minimum of 45 subjects contributing the complete set of 4 samples each (allocated early and late sample following the first IV and PO dose) are required to provide power of >85% to estimate all parameters within 20% precision limits. We estimate that up to 25% of subjects will not provide complete sample sets (either due to missing samples or withdrawal), so plan to recruit approximately 60 subjects to ensure 45 complete sample sets. If all 60 subjects provide complete sample sets, power would rise to 96%. Recruitment will continue until 45 patients in fosfomycin arm have a complete set of PK samples.

## 5. Study Assessments

### 5.1. Schedule of events

A schedule of events identifying the timing of required assessments and investigations (Figure 1).

*Figure 1: Schedule of Events*

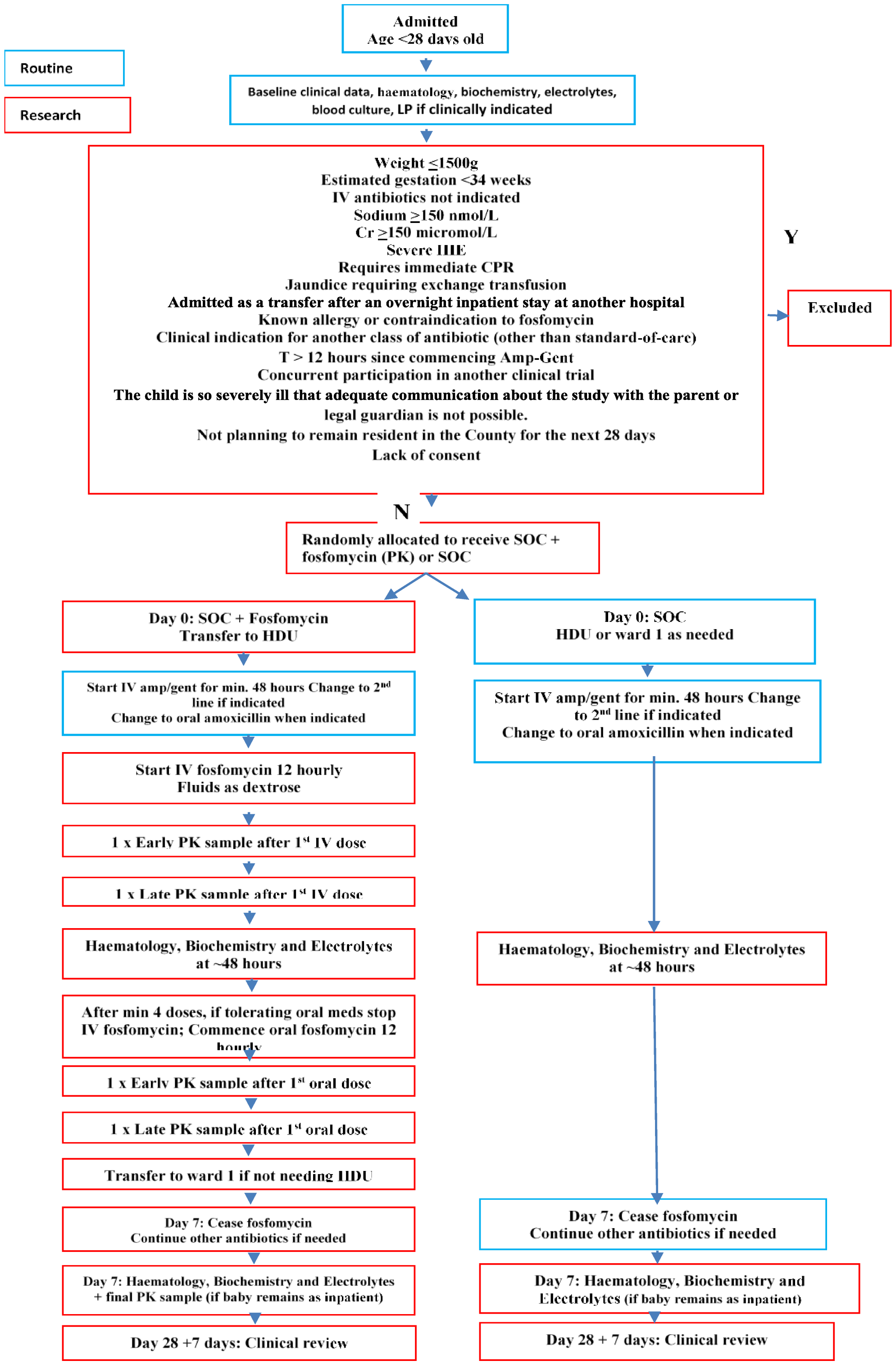

## *5.2. Assessment of Safety*

### *5.2.1. Safety Reporting*

Safety will be assessed through routine monitoring of adverse events. In addition, evaluation of hematology and blood chemistry parameters, regular measurements of vital signs and physical examinations will be conducted as per the protocol and clinical indication.

The frequency, severity, seriousness and causality assessments of AEs (for each study drug) will be described, as well as frequency of SAEs or AEs that lead to treatment discontinuation.

AEs will be collated for each treatment groups in the CRF. The adverse events reporting period begins upon subject enrolment in the trial (after signature of informed consent and ends 28 days + 7 days (4 weeks) after the first dose of study drug(s) is administered.

### 5.2.2. *Clinical Laboratory assessments*

Hematology parameters (CBC, WBC with differential and platelets) will be analyzed at screening, on days 2 and 7 (if baby remains inpatient) as is normally clinically indicated (plus any additional investigations as clinically indicated). Biochemistry and electrolytes parameters will be analysed at screening, on day 2 and day 7 (if baby remains as inpatient). Additional samples may be done if clinically indicated.

### 5.2.3. Anticipated Events in neonatal setting

Anticipated events are relatively common in this patient group due to low birth weight, possible birth asphyxia and concomitant disease process (*protocol page 28*).

- Anticipated events associated with neonatal setting, but which are **not assessed as "AEs** "(not an untoward medical occurrence taking into account the new born pre-exiting conditions and common neonatal setting/conditions**)** will be reported on the CRF **but not as AEs (or SAEs).**
- Anticipated events which are **more severe or more frequent than expected in this neonatal setting** will be reported as **AEs, and assessed for severity, causality and seriousness as any other AE**. If classified as AEs, they must be reported as AEs in the CRF and, if matching any seriousness criteria, on CRF AE and SAE form.

*Table 1: Anticipated Events in Neonatal Setting*

| ANTICIPATED EVENTS IN NEONATAL SETTING |
|---|
| Necrotising enterocolitis (diagnostic radiological/surgical changes) |
| Intracranial abnormality on cranial ultrasound scan (parenchymal haemorrhage or focal white matter injury) |
| Patent ductus arteriosus |
| Pulmonary haemorrhage |
| Severe anaemia requiring transfusion (due to ABO incompatability, prematurity or haemorrhage) |
| Jaundice requiring phototherapy or exchange transfusion |
| Congenital birth defect diagnosed during admission |
| Fracture secondary to birth trauma |
| Apnoea |
| Infection (positive blood culture with clinical signs)* |
| Persistent derangement of liver function tests (beyond 36 weeks CGA) |
| Abnormal muscle tone, posturing or convulsions (secondary to suspected birth asphyxia) |
| An episode of Hypoglycaemia (defined as per the World Health Organization, ≤2.6mmol/L)[47] |

** Both infections other than condition for inclusion in trial and, in case of infections linked to the sepsis episode that is the reason of the participant's inclusion in the trial, worsening (e.g. septic shock) and relapse.*

# 6. Planned Analysis

## *6.1. Interim Analysis*

There is no interim analysis planned for this trial

## *6.2. Final Analysis*

The planned final analyses will be performed only after the last patient has completed assessments scheduled for the day 28 and the database has been cleaned and locked.

Every effort will be made to collect all data as per the schedule of assessments. Where missing data occurs either because of withdrawal of consent, lost to follow-up or omitted due to investigator oversight, the analysis will capture the missing information and summaries will be provided based on available data set. Patients who withdraw consent will be excluded from trial analyses from the time they withdraw. It is assumed that when missing data occurs it will be at random.

Any additional analyses performed to provide support for planned analyses but not identified in this SAP will be documented and reported in the SAR and clearly identified as unplanned analyses in the report.

Results from the final analyses will be reviewed by the trial team prior to completion of the statistical analysis report (SAR).



Obiero CW, *et al. Arch Dis Child* 2022;0:1–9. doi: 10.1136/archdischild-2021-322483

# 7. Statistical Methods

## *7.1 Data Transformation*

There is no data transformation specified in the protocol. However, any data transformations or derived variables that become necessary during analysis will be documented in the STATA analysis programs and described in the SAR and manuscripts developed from the trial.

## *7.2 Baseline Data*

To present baseline data, summaries for continuous variables will include: n, mean, standard deviation (SD), median and interquartile range and other summaries (e.g. minimum, maximum) will be used as appropriate.

Binary and categorical data will be summarized using frequencies and proportions. Reported percentages will be rounded and reported to a single decimal place (xx.x%).

## 7.3 Analyses set

All patients enrolled in the trial and who have received at least one dose of the trial medication will be included in the safety analysis.

## *7.4 Safety Analysis*

The mean difference (95%CI) in plasma sodium, creatinine, potassium and ALT values between 48 hours and day 7 and baseline will be calculated, by treatment arm. Formal testing for a difference between arms will be done using ANOVA comparing the average 48 hour and day 7 absolute values, adjusting for baseline values.

Additionally, safety for each regimen will be based on the incidence of adverse events and laboratory test abnormalities by treatment group.

The following data summaries of adverse events will be documented for each treatment arm:

i. The number (%) of patients experiencing a SAE
ii. The number (%) of patients experiencing a serious adverse drug reaction (SADR), where an adverse drug reaction (ADR) means that the relation to a study drug was recorded by the investigator
iii. The number (%) of patients experiencing an AE (whether serious or not)
iv. The number (%) of patients experiencing an ADR (whether serious or not)
v. The median (range) number of ADR per patient (whether serious or not).
vi. The number of patients (%) whose treatment was stopped due to an AE or other pre-specified reason.

## 7.5 Biological Parameters

### 7.5.1 Shift tables

Shift tables will be used to present changes between baseline and D2 and baseline and D7 (if available). Categorizations will be made into normal, grade 1, grade 2, grade 3, grade 4 and grade 5 according to DAIDS toxicity tables. The number of patients in each category will be given.

### 7.5.2 Graphical representations

Descriptive analyses of the biological parameter data will include the following visual presentations:

i) Box and whisker plots will be created showing the distribution of biological parameters of interest at each measurement time point. The y-axis will show the values of each parameter on the original measurement scale. The x-axis will show the measurement times in terms of treatment day (i.e. day 0 (baseline), 2 and 7 (if available))

ii) Scatterplots of hematological parameters with baseline values on the x-axis and D2, D7 (if available) values on the y-axis for.

iii) Scatterplots for sodium with baseline values on the x-axis and D2, D7 if available on the y-axis.

iv) Line graphs showing values over time as individual lines for each patient connecting measurements at baseline and all subsequent measurements up to and D7 (if available). There will be one graph per parameter.

## 7.6 Software

Analysis for this SAP will be done using STATA version 15.1

# 8. Results

## 8.1. Trial Profile

All patients who provide informed consent will be accounted for in the SAR trial from initial screening for eligibility to completion of the final analysis through a CONSORT flow diagram (*Figure 2*). The summary will include number by treatment group for patient's population and reasons for trial withdrawal.

*Figure 2: Participant Flow*

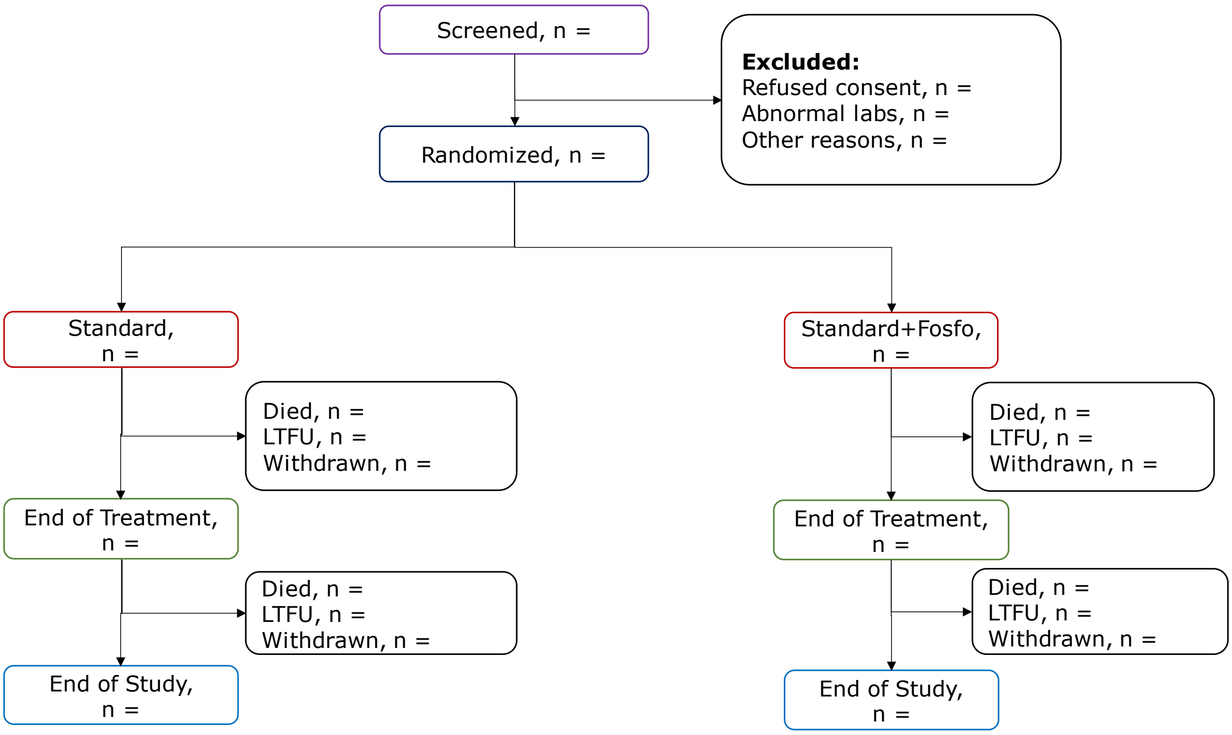


## 8.2. Patient Characteristics and baseline comparisons

A summary of enrolled patients based on eligibility criteria at the two timepoints (*before and after protocol amendment*) will be provided.

Demographic and other baseline characteristics will be summarized by treatment group. Categorical variables will be summarized by frequencies and percentages. Percentages will be calculated according to the number of patients for whom data are available. Where values are missing, the denominator, which will be less than the number of patients assigned to the treatment group, will be reported either in the body or a footnote in the summary table. Continuous variables will be summarized in terms of mean and standard deviation as well as quartiles.

Additionally, examination (*protocol section 6*) feeding practices (*protocol section 12*), Birth history (*protocol section 11*), clinicians' impression of risk (*protocol section 9*) and suspected

initial diagnosis (*protocol section 8*) will be reported as part of list of listings in the appendices.

*Table 2: Baseline demographic characteristics by the treatment group*

| Parameter | Statistic | SOC (n=) | SOC + Fosfo (n=) | Overall (n=) |
|---|---|---|---|---|
| Age (days) | N | | | |
| | Mean (SD) | | | |
| | Median (IQR) | | | |
| Sex, n (%) | Female | | | |
| | Male | | | |
| Estimated gestational age | Range (min-max) | | | |

SOC=standard of care

*Table 3: Baseline anthropometric and vital signs by the treatment group*

| Parameter | Statistic | SOC (n=) | SOC + Fosfo (n=) | Overall (n=) |
|---|---|---|---|---|
| Weight (grams) | Mean (SD) | | | |
| | Median (IQR) | | | |
| | Median (IQR) | | | |
| Head circumference (cm) | Range (min-max) | | | |
| | Mean (SD) | | | |
| | Median (IQR) | | | |
| Length (cm) | Range (min-max) | | | |
| | Mean (SD) | | | |
| | Median (IQR) | | | |
| Axillary Temperature (°C) | Range (min-max) | | | |
| | Mean (SD) | | | |
| | Median (IQR) | | | |
| Heart rate (bpm) | Range (min-max) | | | |
| | Mean (SD) | | | |
| | Median (IQR) | | | |
| Respiratory rate (bpm) | Range (min-max) | | | |
| | Mean (SD) | | | |
| | Median (IQR) | | | |

SOC=standard of care; SD=standard deviation; IQR=inter quartile range



Obiero CW, *et al. Arch Dis Child* 2022;0:1–9. doi: 10.1136/archdischild-2021-322483

*Table 4: Baseline clinical symptoms by the treatment group*

| | SOC (n=) | SOC + Fosfo (n=) | Overall (n=) |
|---|---|---|---|
| Clinical Symptoms and signs | | | |
| Fever | | | |
| Difficulty breathing | | | |
| Rash | | | |
| Vomiting | | | |
| Altered consciousness | | | |
| Not feeding | | | |
| Lethargy | | | |
| Seizures | | | |
| Other signs | | | |

SOC=standard of care

*Table 5: Past medical history and concomitant illness by the treatment group*

| | SOC (n=) | SOC + Fosfo (n=) | Overall (n=) |
|---|---|---|---|
| Past medical history | | | |
| Respiratory | | | |
| General | | | |
| Infection | | | |
| CNS e.g. seizures | | | |

SOC=standard of care

*Table 6: Baseline summary of haematological parameters by treatment group*

| Parameter | Statistic | SOC (n=) | SOC + Fosfo (n=) | Overall (n=) |
|---|---|---|---|---|
| Hb (g/dL) | Range (min-max) | | | |
| | Mean (SD) | | | |
| | Median (IQR) | | | |
| MCV (fl) | Range (min-max) | | | |
| | Mean (SD) | | | |
| | Median (IQR) | | | |
| WBC ($x10^3/\mu L$) | Range (min-max) | | | |
| | Mean (SD) | | | |
| | Median (IQR) | | | |
| Neutrophils ($x10^3/\mu L$) | Range (min-max) | | | |
| | Mean (SD) | | | |
| | Median (IQR) | | | |
| Lymphocytes ($x10^3/\mu L$) | Range (min-max) | | | |
| | Mean (SD) | | | |
| | Median (IQR) | | | |

| | | | | |
|---|---|---|---|---|
| Monocytes ($x10^3$/μL) | Range (min-max) | | | |
| | Mean (SD) | | | |
| | Median (IQR) | | | |
| Eosinophils ($x10^3$/μL) | Range (min-max) | | | |
| | Mean (SD) | | | |
| | Median (IQR) | | | |
| Basophils ($x10^3$/μL) | Range (min-max) | | | |
| | Mean (SD) | | | |
| | Median (IQR) | | | |
| Platelets ($x10^3$/μL) | Range (min-max) | | | |
| | Mean (SD) | | | |
| | Median (IQR) | | | |

SOC=standard of care; SD=standard deviation; IQR=inter quartile range

*Table 7: Baseline summary of blood chemistry parameters by treatment group*

| Parameter | Statistic | SOC (n=) | SOC + Fosfo (n=) | Overall (n=) |
|---|---|---|---|---|
| Creatinine (CRE) (μmol/L) | Range (min-max) | | | |
| | Mean (SD) | | | |
| | Median (IQR) | | | |
| Sodium (mmol/L) | Range (min-max) | | | |
| | Mean (SD) | | | |
| | Median (IQR) | | | |
| Potassium (mmol/L) | Range (min-max) | | | |
| | Mean (SD) | | | |
| | Median (IQR) | | | |
| Alanine transaminase (ALT) (U/L) | Range (min-max) | | | |
| | Mean (SD) | | | |
| | Median (IQR) | | | |
| Aspartate aminotransferase (AST) (U/L) | Range (min-max) | | | |
| | Mean (SD) | | | |
| | Median (IQR) | | | |
| Alkaline Phosphatase (μmol/L) | Range (min-max) | | | |
| | Mean (SD) | | | |
| | Median (IQR) | | | |
| Albumin (g/dL) | Range (min-max) | | | |
| | Mean (SD) | | | |
| | Median (IQR) | | | |
| Total bilirubin (TBIL) (μmol/L) | Range (min-max) | | | |
| | Mean (SD) | | | |
| | Median (IQR) | | | |
| Calcium (mmol/L) | Range (min-max) | | | |
| | Mean (SD) | | | |
| | Median (IQR) | | | |
| Magnesium (mmol/L) | Range (min-max) | | | |
| | Mean (SD) | | | |

| | | |
|---|---|---|
| | Median (IQR) | |
| Phosphate (U/L) | Range (min-max) | |
| | Mean (SD) | |
| | Median (IQR) | |

SOC=standard of care; SD=standard deviation; IQR=inter quartile range

A summary on positive blood or CSF culture at admission will be reported here.

A summary of the relevant clinical events will be reported here.

*Table 8: Baseline summary of Blood Gas parameters by treatment group*

| Parameter | Statistic | SOC (n=) | SOC + Fosfo (n=) | Overall (n=) |
|---|---|---|---|---|
| PH | Range (min-max) | | | |
| | Mean (SD) | | | |
| | Median (IQR) | | | |
| $PO_2$ (mmHg/kpa) | Range (min-max) | | | |
| | Mean (SD) | | | |
| | Median (IQR) | | | |
| $PC0_2$ (mmHg/kpa) | Range (min-max) | | | |
| | Mean (SD) | | | |
| | Median (IQR) | | | |
| Bicarb (mmol/L) | Range (min-max) | | | |
| | Mean (SD) | | | |
| | Median (IQR) | | | |
| Chloride (mmol/L) | Range (min-max) | | | |
| | Mean (SD) | | | |
| | Median (IQR) | | | |
| Lactate (mmol/L) | Range (min-max) | | | |
| | Mean (SD) | | | |
| | Median (IQR) | | | |

SOC=standard of care; SD=standard deviation; IQR=inter quartile range

A summary of the results of CSF/Blood culture (including sensitivity) will be captured here.

## *8.3. Exploratory Analysis*

Box plot and profile plots will be used in exploring data distributions as well as identifying possible outliers. Exploratory analysis will also involve subgroup and subset analysis. The shift tables will be used in exploring the change in lab values at during and post-treatment visits from the baseline.

### *8.3.1. Graphical representations*

Descriptive analyses of the biological parameter data will include box plots, scatter plots and line graphs where appropriate. Box plots will be created showing the distribution of biological parameters of interest at each measurement time point for two regimens side-by-side. Line graphs showing values over time as individual lines for each patient connecting measurements at baseline and all subsequent measurements. The following are the planned graphs by treatment group;

*Figure 3:Time trajectories for heart rate (bpm)*

*Figure 4: Box plot for heart rate (bpm)*

*Figure 5:Time trajectories for respiratory rate (bpm)*

*Figure 6: Box plot for respiratory rate (bpm)*

*Figure 7:Time trajectories for temperature (°c)*

*Figure 8: Box plot for temperature (°c)*
*Figure 9:Time trajectories for $SaO_2$ (%)*
*Figure 10: Box plot for $SaO_2$ (%)*
*Figure 11:Time trajectories for White cell count ($x10^3$/μL)*
*Figure 12: Box plot for White cell count ($x10^3$/μL)*
*Figure 13: Scatter plot for White cell count ($x10^3$/μL)*
*Figure 14: Time trajectories for SGPT/ALT (U/L)*
*Figure 15: Box plot for SGPT/ALT (U/L)*
*Figure 16: Scatter plot for SGPT/ALT (U/L)*
*Figure 17: Time trajectories for Creatinine (μmol/L)*
*Figure 18: Box plot for Creatinine (μmol/L)*
*Figure 19: Scatter plot for Creatinine (μmol/L)*
*Figure 20. Time trajectories for Sodium (mmol/L)*
*Figure 21: Box plot for Sodium (mmol/L)*
*Figure 22: Scatter plot for Sodium (mmol/L)*
*Figure 23. Time trajectories for Potassium (mmol/L)*
*Figure 24: Box plot for Potassium (mmol/L)*
*Figure 25: Scatter plot for Potassium (mmol/L)*

### *8.3.2. Change in Lab values*

Lab shift tables (*See Tables in appendix*) will be used to present changes in lab values between baseline and subsequent visits. Categorizations will be made into grade 1, grade 2, grade 3 and grade 4 according to DAIDS toxicity tables. The number of patients in each category and percentage change from baseline will be given.



Obiero CW, *et al. Arch Dis Child* 2022;0:1–9. doi: 10.1136/archdischild-2021-322483

### 8.4. *Safety Analyses: Sodium, Creatinine & Potassium*

*Table 9: Plasma Sodium Concentrations*

| 48 hours | Sodium plasma concentration | Mean Difference | p-value* |
|---|---|---|---|
| | Mean (SD) | | |
| Std, n= | | | |
| Std+Fosfo, n= | | | |
| | | | |
| Day 7 | Mean (SD) | | |
| Std, n= | | | |
| Std+Fosfo, n= | | | |

* p-value from ANOVA test of difference at (48hr or day 7) between arms, adjusting for baseline values

*Table 10: Creatinine*

| 48 hours | Creatinine | Mean Difference | p-value* |
|---|---|---|---|
| | Mean (SD) | | |
| Std, n= | | | |
| Std+Fosfo, n= | | | |
| | | | |
| Day 7 | Mean (SD) | | |
| Std, n= | | | |
| Std+Fosfo, n= | | | |

* p-value from ANOVA test of difference at (48hr or day 7) between arms, adjusting for baseline values

*Table 11: Potassium Concentrations*

| 48 hours | Potassium | Mean Difference | p-value* |
|---|---|---|---|
| | Mean (SD) | | |
| Std, n= | | | |
| Std+Fosfo, n= | | | |
| | | | |
| Day 7 | Mean (SD) | | |
| Std, n= | | | |
| Std+Fosfo, n= | | | |

* p-value from ANOVA test of difference at (48hr or day 7) between arms, adjusting for baseline values

*Table 12: ALT*

| 48 hours | ALT | Mean Difference | p-value* |
|---|---|---|---|
| | Mean (SD) | | |
| Std, n= | | | |
| Std+Fosfo, n= | | | |
| | | | |
| Day 7 | Mean (SD) | | |
| Std, n= | | | |
| Std+Fosfo, n= | | | |

* p-value from ANOVA test of difference at (48hr or day 7) between arms, adjusting for baseline values

### *8.5. Safety Analysis: Adverse Events*

*Table 13: Number of patients experiencing adverse events*

| | SOC | SOC + Fosfo | Overall |
|---|---|---|---|
| Number enrolled and receiving at least one dose | | | |
| **Number of patients with at least one SAE: n (%)** | | | |
| Total* | | | |
| Adverse drug reaction (ADR) related to std or fosfomycin | | | |
| Not related to study drug | | | |
| **Patients with at least one AE (serious or not): n (%)** | | | |
| Total* | | | |
| Adverse drug reaction (ADR) related to std or fosfomycin | | | |
| Not related to study drug | | | |
| **Patients with at least one ADR (serious or not) by intensity: n (%)** | | | |
| Mild | | | |
| Moderate | | | |
| Severe | | | |
| Life threatening | | | |
| Death | | | |
| **Patients whose treatment was stopped due to an AE (serious or not): n (%)** | | | |
| Total | | | |
| Adverse drug reaction (ADR) related to std or fosfomycin | | | |
| Not related to study drug | | | |
| **Patients with anticipated AE's (related and unrelated)** | | | |
| Median | | | |
| Range | | | |
| **Number of non-ADR per patient** | | | |
| Median | | | |
| Range | | | |
| **Number of ADR per patient** | | | |
| Median | | | |
| Range | | | |
| | | | |
| Total person days at risk† | | | |
| Unadjusted rate per day (95% CI) | | | |
| | | | |
| **Patients experiencing ≥1 DAIDS grade 3 or 4 ADR, n (%)** | | | |

AE=Adverse Event; ADR=Adverse Drug Reaction; SAE=Serious Adverse Event; SOC=Standard of care
*rows do not necessarily add to the total number of patients as a single patient may have AE's in multiple rows.
†time at risk is from day 1 to 28 or day of death if before D28.

Additional summary detail on ADR if on oral or IV fosfomycin will be provided here.

*Table 14. Serious adverse events listing*

| ID | Onset day (Study Day) | System Organ Class | MedDRa Preferred Term | Relation to study drug* | Intensity | Outcome |
|---|---|---|---|---|---|---|
| | | | | | | |

**indicate which drug if available*

*Table 15. Treatment discontinuation: AE and other reasons*

| ID | Days administered (total dose) | System Organ Class | MedDRa Preferred Term | Corresponding laboratory parameter value | Relation to study Drug* | Intensity | Rescue medication administered (Yes/No) |
|---|---|---|---|---|---|---|---|
| | | | | | | | |

**indicate which drug if available*

*Table 16. Serious and non-serious AEs by relationship to study medication*

| System Organ Class | Preferred MedDRa Term | NR | ADR |
|---|---|---|---|
| | | | |

*Note: Data are numbers of AE rather than number of patients with an AE of each type*

*Table 17. Serious and non-serious AEs by severity*

| System Organ Class | Preferred MedDRa Term | Grade 1 n (%) [] | Grade 2 n (%) [] | Grade 3 n (%) [] | Grade 4 n (%) [] | Grade 5 n (%) [] |
|---|---|---|---|---|---|---|
| | | | | | | |

*Note: Data are number of patients (percent of patients) [number of events].*

*Table 18: Anticipated events by the treatment group*

| | SOC (n=) | SOC + Fosfo (n=) | Overall (n=) |
|---|---|---|---|
| Necrotising enterocolitis (diagnostic radiological/surgical changes) | | | |
| Intracranial abnormality on cranial ultrasound scan (parenchymal haemorrhage or focal white matter injury) | | | |
| Patent ductus arteriosus | | | |
| Pulmonary haemorrhage | | | |
| Severe anaemia requiring transfusion (due to ABO incompatibility, prematurity or haemorrhage) | | | |
| Jaundice requiring phototherapy or exchange transfusion | | | |
| Congenital birth defect diagnosed during admission | | | |
| Fracture secondary to birth trauma | | | |
| Apnoea | | | |
| Infection (positive blood culture with clinical signs)* | | | |
| Persistent derangement of liver function tests (beyond 36 weeks CGA) | | | |
| Abnormal muscle tone, posturing or convulsions (secondary to suspected birth asphyxia) | | | |
| An episode of Hypoglycaemia (defined as per the World Health Organization, ≤2.6mmol/L) | | | |

**SOC=standard of care**
*** Both infections other than condition for inclusion in trial and, in case of infections linked to the sepsis episode that is the reason of the participant's inclusion in the trial, worsening (e.g. septic shock) and relapse.**

*Table 19: Shift table for Haemoglobin*

| Baseline (Day 0) n = | | Day 2 n = | | | | | | Day 7 n = | | | | | |
|---|---|---|---|---|---|---|---|---|---|---|---|---|---|
| Grade | | Normal | 1 | 2 | 3 | 4 | 5 | Normal | 1 | 2 | 3 | 4 | 5 |
| SOC | Normal | *n (%)* | *n (%)* | *n (%)* | *n (%)* | *n (%)* | *n (%)* | *n (%)* | *n (%)* | *n (%)* | *n (%)* | *n (%)* | *n (%)* |
| | 1 | *n (%)* | *n (%)* | *n (%)* | *n (%)* | *n (%)* | *n (%)* | *n (%)* | *n (%)* | *n (%)* | *n (%)* | *n (%)* | *n (%)* |
| | 2 | *n (%)* | *n (%)* | *n (%)* | *n (%)* | *n (%)* | *n (%)* | *n (%)* | *n (%)* | *n (%)* | *n (%)* | *n (%)* | *n (%)* |
| | 3 | *n (%)* | *n (%)* | *n (%)* | *n (%)* | *n (%)* | *n (%)* | *n (%)* | *n (%)* | *n (%)* | *n (%)* | *n (%)* | *n (%)* |
| | 4 | *n (%)* | *n (%)* | *n (%)* | *n (%)* | *n (%)* | *n (%)* | *n (%)* | *n (%)* | *n (%)* | *n (%)* | *n (%)* | *n (%)* |
| | 5 | *n (%)* | *n (%)* | *n (%)* | *n (%)* | *n (%)* | *n (%)* | *n (%)* | *n (%)* | *n (%)* | *n (%)* | *n (%)* | *n (%)* |
| SOC + Fosfo | Normal | *n (%)* | *n (%)* | *n (%)* | *n (%)* | *n (%)* | *n (%)* | *n (%)* | *n (%)* | *n (%)* | *n (%)* | *n (%)* | *n (%)* |
| | 1 | *n (%)* | *n (%)* | *n (%)* | *n (%)* | *n (%)* | *n (%)* | *n (%)* | *n (%)* | *n (%)* | *n (%)* | *n (%)* | *n (%)* |
| | 2 | *n (%)* | *n (%)* | *n (%)* | *n (%)* | *n (%)* | *n (%)* | *n (%)* | *n (%)* | *n (%)* | *n (%)* | *n (%)* | *n (%)* |
| | 3 | *n (%)* | *n (%)* | *n (%)* | *n (%)* | *n (%)* | *n (%)* | *n (%)* | *n (%)* | *n (%)* | *n (%)* | *n (%)* | *n (%)* |
| | 4 | *n (%)* | *n (%)* | *n (%)* | *n (%)* | *n (%)* | *n (%)* | *n (%)* | *n (%)* | *n (%)* | *n (%)* | *n (%)* | *n (%)* |
| | 5 | *n (%)* | *n (%)* | *n (%)* | *n (%)* | *n (%)* | *n (%)* | *n (%)* | *n (%)* | *n (%)* | *n (%)* | *n (%)* | *n (%)* |

SOC=standard of care

| DNDi AFRICA Drugs for Neglected Diseases initiative | Statistical Analysis Plan (SAP) | NEO_FOSFO_SAP_V0.1 | 18-Mar-19 |
|---|---|---|---|

*Table 20: Shift table for White Blood Cells*

| Baseline (Day 0) | | Day 2 | | | | | | Day 7 | | | | | |
|---|---|---|---|---|---|---|---|---|---|---|---|---|---|
| Grade | | Normal | 1 | 2 | 3 | 4 | 5 | Normal | 1 | 2 | 3 | 4 | 5 |
| SOC | Normal | *n (%)* | *n (%)* | *n (%)* | *n (%)* | *n (%)* | *n (%)* | *n (%)* | *n (%)* | *n (%)* | *n (%)* | *n (%)* | *n (%)* |
| | 1 | *n (%)* | *n (%)* | *n (%)* | *n (%)* | *n (%)* | *n (%)* | *n (%)* | *n (%)* | *n (%)* | *n (%)* | *n (%)* | *n (%)* |
| | 2 | *n (%)* | *n (%)* | *n (%)* | *n (%)* | *n (%)* | *n (%)* | *n (%)* | *n (%)* | *n (%)* | *n (%)* | *n (%)* | *n (%)* |
| | 3 | *n (%)* | *n (%)* | *n (%)* | *n (%)* | *n (%)* | *n (%)* | *n (%)* | *n (%)* | *n (%)* | *n (%)* | *n (%)* | *n (%)* |
| | 4 | *n (%)* | *n (%)* | *n (%)* | *n (%)* | *n (%)* | *n (%)* | *n (%)* | *n (%)* | *n (%)* | *n (%)* | *n (%)* | *n (%)* |
| | 5 | *n (%)* | *n (%)* | *n (%)* | *n (%)* | *n (%)* | *n (%)* | *n (%)* | *n (%)* | *n (%)* | *n (%)* | *n (%)* | *n (%)* |
| SOC + Fosfo | Normal | *n (%)* | *n (%)* | *n (%)* | *n (%)* | *n (%)* | *n (%)* | *n (%)* | *n (%)* | *n (%)* | *n (%)* | *n (%)* | *n (%)* |
| | 1 | *n (%)* | *n (%)* | *n (%)* | *n (%)* | *n (%)* | *n (%)* | *n (%)* | *n (%)* | *n (%)* | *n (%)* | *n (%)* | *n (%)* |
| | 2 | *n (%)* | *n (%)* | *n (%)* | *n (%)* | *n (%)* | *n (%)* | *n (%)* | *n (%)* | *n (%)* | *n (%)* | *n (%)* | *n (%)* |
| | 3 | *n (%)* | *n (%)* | *n (%)* | *n (%)* | *n (%)* | *n (%)* | *n (%)* | *n (%)* | *n (%)* | *n (%)* | *n (%)* | *n (%)* |
| | 4 | *n (%)* | *n (%)* | *n (%)* | *n (%)* | *n (%)* | *n (%)* | *n (%)* | *n (%)* | *n (%)* | *n (%)* | *n (%)* | *n (%)* |
| | 5 | *n (%)* | *n (%)* | *n (%)* | *n (%)* | *n (%)* | *n (%)* | *n (%)* | *n (%)* | *n (%)* | *n (%)* | *n (%)* | *n (%)* |

SOC=standard of care

*Table 21: Shift table for Neutrophils*

| Baseline (Day 0) | | Day 2 | | | | | | Day 7 | | | | | |
|---|---|---|---|---|---|---|---|---|---|---|---|---|---|
| Grade | | Normal | 1 | 2 | 3 | 4 | 5 | Normal | 1 | 2 | 3 | 4 | 5 |
| SOC | Normal | *n (%)* | *n (%)* | *n (%)* | *n (%)* | *n (%)* | *n (%)* | *n (%)* | *n (%)* | *n (%)* | *n (%)* | *n (%)* | *n (%)* |
| | 1 | *n (%)* | *n (%)* | *n (%)* | *n (%)* | *n (%)* | *n (%)* | *n (%)* | *n (%)* | *n (%)* | *n (%)* | *n (%)* | *n (%)* |
| | 2 | *n (%)* | *n (%)* | *n (%)* | *n (%)* | *n (%)* | *n (%)* | *n (%)* | *n (%)* | *n (%)* | *n (%)* | *n (%)* | *n (%)* |
| | 3 | *n (%)* | *n (%)* | *n (%)* | *n (%)* | *n (%)* | *n (%)* | *n (%)* | *n (%)* | *n (%)* | *n (%)* | *n (%)* | *n (%)* |
| | 4 | *n (%)* | *n (%)* | *n (%)* | *n (%)* | *n (%)* | *n (%)* | *n (%)* | *n (%)* | *n (%)* | *n (%)* | *n (%)* | *n (%)* |
| | 5 | *n (%)* | *n (%)* | *n (%)* | *n (%)* | *n (%)* | *n (%)* | *n (%)* | *n (%)* | *n (%)* | *n (%)* | *n (%)* | *n (%)* |
| SOC + Fosfo | Normal | *n (%)* | *n (%)* | *n (%)* | *n (%)* | *n (%)* | *n (%)* | *n (%)* | *n (%)* | *n (%)* | *n (%)* | *n (%)* | *n (%)* |
| | 1 | *n (%)* | *n (%)* | *n (%)* | *n (%)* | *n (%)* | *n (%)* | *n (%)* | *n (%)* | *n (%)* | *n (%)* | *n (%)* | *n (%)* |
| | 2 | *n (%)* | *n (%)* | *n (%)* | *n (%)* | *n (%)* | *n (%)* | *n (%)* | *n (%)* | *n (%)* | *n (%)* | *n (%)* | *n (%)* |
| | 3 | *n (%)* | *n (%)* | *n (%)* | *n (%)* | *n (%)* | *n (%)* | *n (%)* | *n (%)* | *n (%)* | *n (%)* | *n (%)* | *n (%)* |
| | 4 | *n (%)* | *n (%)* | *n (%)* | *n (%)* | *n (%)* | *n (%)* | *n (%)* | *n (%)* | *n (%)* | *n (%)* | *n (%)* | *n (%)* |
| | 5 | *n (%)* | *n (%)* | *n (%)* | *n (%)* | *n (%)* | *n (%)* | *n (%)* | *n (%)* | *n (%)* | *n (%)* | *n (%)* | *n (%)* |

SOC=standard of care



Obiero CW, *et al. Arch Dis Child* 2022;0:1–9. doi: 10.1136/archdischild-2021-322483

*Table 22: Shift table for Lymphocytes*

| Baseline (Day 0) | | Day 2 | | | | | | Day 7 | | | | | |
|---|---|---|---|---|---|---|---|---|---|---|---|---|---|
| Grade | | Normal | 1 | 2 | 3 | 4 | 5 | Normal | 1 | 2 | 3 | 4 | 5 |
| SOC | Normal | *n (%)* | *n (%)* | *n (%)* | *n (%)* | *n (%)* | *n (%)* | *n (%)* | *n (%)* | *n (%)* | *n (%)* | *n (%)* | *n (%)* |
| | 1 | *n (%)* | *n (%)* | *n (%)* | *n (%)* | *n (%)* | *n (%)* | *n (%)* | *n (%)* | *n (%)* | *n (%)* | *n (%)* | *n (%)* |
| | 2 | *n (%)* | *n (%)* | *n (%)* | *n (%)* | *n (%)* | *n (%)* | *n (%)* | *n (%)* | *n (%)* | *n (%)* | *n (%)* | *n (%)* |
| | 3 | *n (%)* | *n (%)* | *n (%)* | *n (%)* | *n (%)* | *n (%)* | *n (%)* | *n (%)* | *n (%)* | *n (%)* | *n (%)* | *n (%)* |
| | 4 | *n (%)* | *n (%)* | *n (%)* | *n (%)* | *n (%)* | *n (%)* | *n (%)* | *n (%)* | *n (%)* | *n (%)* | *n (%)* | *n (%)* |
| | 5 | *n (%)* | *n (%)* | *n (%)* | *n (%)* | *n (%)* | *n (%)* | *n (%)* | *n (%)* | *n (%)* | *n (%)* | *n (%)* | *n (%)* |
| SOC + Fosfo | Normal | *n (%)* | *n (%)* | *n (%)* | *n (%)* | *n (%)* | *n (%)* | *n (%)* | *n (%)* | *n (%)* | *n (%)* | *n (%)* | *n (%)* |
| | 1 | *n (%)* | *n (%)* | *n (%)* | *n (%)* | *n (%)* | *n (%)* | *n (%)* | *n (%)* | *n (%)* | *n (%)* | *n (%)* | *n (%)* |
| | 2 | *n (%)* | *n (%)* | *n (%)* | *n (%)* | *n (%)* | *n (%)* | *n (%)* | *n (%)* | *n (%)* | *n (%)* | *n (%)* | *n (%)* |
| | 3 | *n (%)* | *n (%)* | *n (%)* | *n (%)* | *n (%)* | *n (%)* | *n (%)* | *n (%)* | *n (%)* | *n (%)* | *n (%)* | *n (%)* |
| | 4 | *n (%)* | *n (%)* | *n (%)* | *n (%)* | *n (%)* | *n (%)* | *n (%)* | *n (%)* | *n (%)* | *n (%)* | *n (%)* | *n (%)* |
| | 5 | *n (%)* | *n (%)* | *n (%)* | *n (%)* | *n (%)* | *n (%)* | *n (%)* | *n (%)* | *n (%)* | *n (%)* | *n (%)* | *n (%)* |

SOC=standard of care

*Table 23: Shift table for Platelets*

| Baseline (Day 0) | | Day 2 | | | | | | Day 7 | | | | | |
|---|---|---|---|---|---|---|---|---|---|---|---|---|---|
| Grade | | Normal | 1 | 2 | 3 | 4 | 5 | Normal | 1 | 2 | 3 | 4 | 5 |
| SOC | Normal | *n (%)* | *n (%)* | *n (%)* | *n (%)* | *n (%)* | *n (%)* | *n (%)* | *n (%)* | *n (%)* | *n (%)* | *n (%)* | *n (%)* |
| | 1 | *n (%)* | *n (%)* | *n (%)* | *n (%)* | *n (%)* | *n (%)* | *n (%)* | *n (%)* | *n (%)* | *n (%)* | *n (%)* | *n (%)* |
| | 2 | *n (%)* | *n (%)* | *n (%)* | *n (%)* | *n (%)* | *n (%)* | *n (%)* | *n (%)* | *n (%)* | *n (%)* | *n (%)* | *n (%)* |
| | 3 | *n (%)* | *n (%)* | *n (%)* | *n (%)* | *n (%)* | *n (%)* | *n (%)* | *n (%)* | *n (%)* | *n (%)* | *n (%)* | *n (%)* |
| | 4 | *n (%)* | *n (%)* | *n (%)* | *n (%)* | *n (%)* | *n (%)* | *n (%)* | *n (%)* | *n (%)* | *n (%)* | *n (%)* | *n (%)* |
| | 5 | *n (%)* | *n (%)* | *n (%)* | *n (%)* | *n (%)* | *n (%)* | *n (%)* | *n (%)* | *n (%)* | *n (%)* | *n (%)* | *n (%)* |
| SOC + Fosfo | Normal | *n (%)* | *n (%)* | *n (%)* | *n (%)* | *n (%)* | *n (%)* | *n (%)* | *n (%)* | *n (%)* | *n (%)* | *n (%)* | *n (%)* |
| | 1 | *n (%)* | *n (%)* | *n (%)* | *n (%)* | *n (%)* | *n (%)* | *n (%)* | *n (%)* | *n (%)* | *n (%)* | *n (%)* | *n (%)* |
| | 2 | *n (%)* | *n (%)* | *n (%)* | *n (%)* | *n (%)* | *n (%)* | *n (%)* | *n (%)* | *n (%)* | *n (%)* | *n (%)* | *n (%)* |
| | 3 | *n (%)* | *n (%)* | *n (%)* | *n (%)* | *n (%)* | *n (%)* | *n (%)* | *n (%)* | *n (%)* | *n (%)* | *n (%)* | *n (%)* |
| | 4 | *n (%)* | *n (%)* | *n (%)* | *n (%)* | *n (%)* | *n (%)* | *n (%)* | *n (%)* | *n (%)* | *n (%)* | *n (%)* | *n (%)* |
| | 5 | *n (%)* | *n (%)* | *n (%)* | *n (%)* | *n (%)* | *n (%)* | *n (%)* | *n (%)* | *n (%)* | *n (%)* | *n (%)* | *n (%)* |

SOC=standard of care

| DNDi AFRICA Drugs for Neglected Diseases *initiative* | **Statistical Analysis Plan (SAP)** | **NEO_FOSFO_SAP_V0.1** | **18-Mar-19** |
|---|---|---|---|

*Table 24: Shift table for Sodium*

| Baseline (Day 0) | | Day 2 | | | | | | Day 7 | | | | | |
|---|---|---|---|---|---|---|---|---|---|---|---|---|---|
| Grade | | Normal | 1 | 2 | 3 | 4 | 5 | Normal | 1 | 2 | 3 | 4 | 5 |
| SOC | Normal | *n (%)* | *n (%)* | *n (%)* | *n (%)* | *n (%)* | *n (%)* | *n (%)* | *n (%)* | *n (%)* | *n (%)* | *n (%)* | *n (%)* |
| | 1 | *n (%)* | *n (%)* | *n (%)* | *n (%)* | *n (%)* | *n (%)* | *n (%)* | *n (%)* | *n (%)* | *n (%)* | *n (%)* | *n (%)* |
| | 2 | *n (%)* | *n (%)* | *n (%)* | *n (%)* | *n (%)* | *n (%)* | *n (%)* | *n (%)* | *n (%)* | *n (%)* | *n (%)* | *n (%)* |
| | 3 | *n (%)* | *n (%)* | *n (%)* | *n (%)* | *n (%)* | *n (%)* | *n (%)* | *n (%)* | *n (%)* | *n (%)* | *n (%)* | *n (%)* |
| | 4 | *n (%)* | *n (%)* | *n (%)* | *n (%)* | *n (%)* | *n (%)* | *n (%)* | *n (%)* | *n (%)* | *n (%)* | *n (%)* | *n (%)* |
| | 5 | *n (%)* | *n (%)* | *n (%)* | *n (%)* | *n (%)* | *n (%)* | *n (%)* | *n (%)* | *n (%)* | *n (%)* | *n (%)* | *n (%)* |
| SOC + Fosfo | Normal | *n (%)* | *n (%)* | *n (%)* | *n (%)* | *n (%)* | *n (%)* | *n (%)* | *n (%)* | *n (%)* | *n (%)* | *n (%)* | *n (%)* |
| | 1 | *n (%)* | *n (%)* | *n (%)* | *n (%)* | *n (%)* | *n (%)* | *n (%)* | *n (%)* | *n (%)* | *n (%)* | *n (%)* | *n (%)* |
| | 2 | *n (%)* | *n (%)* | *n (%)* | *n (%)* | *n (%)* | *n (%)* | *n (%)* | *n (%)* | *n (%)* | *n (%)* | *n (%)* | *n (%)* |
| | 3 | *n (%)* | *n (%)* | *n (%)* | *n (%)* | *n (%)* | *n (%)* | *n (%)* | *n (%)* | *n (%)* | *n (%)* | *n (%)* | *n (%)* |
| | 4 | *n (%)* | *n (%)* | *n (%)* | *n (%)* | *n (%)* | *n (%)* | *n (%)* | *n (%)* | *n (%)* | *n (%)* | *n (%)* | *n (%)* |
| | 5 | *n (%)* | *n (%)* | *n (%)* | *n (%)* | *n (%)* | *n (%)* | *n (%)* | *n (%)* | *n (%)* | *n (%)* | *n (%)* | *n (%)* |

SOC=standard of care



Obiero CW, *et al. Arch Dis Child* 2022;0:1–9. doi: 10.1136/archdischild-2021-322483

| DNDi AFRICA Drugs for Neglected Diseases *initiative* | **Statistical Analysis Plan (SAP)** | **NEO_FOSFO_SAP_V0.1** | **18-Mar-19** |
|---|---|---|---|

*Table 25: Shift table for SGOT/AST*

| Baseline (Day 0) | | Day 2 | | | | | | Day 7 | | | | | |
|---|---|---|---|---|---|---|---|---|---|---|---|---|---|
| | Grade | Normal | 1 | 2 | 3 | 4 | 5 | Normal | 1 | 2 | 3 | 4 | 5 |
| SOC | Normal | *n (%)* | *n (%)* | *n (%)* | *n (%)* | *n (%)* | *n (%)* | *n (%)* | *n (%)* | *n (%)* | *n (%)* | *n (%)* | *n (%)* |
| | 1 | *n (%)* | *n (%)* | *n (%)* | *n (%)* | *n (%)* | *n (%)* | *n (%)* | *n (%)* | *n (%)* | *n (%)* | *n (%)* | *n (%)* |
| | 2 | *n (%)* | *n (%)* | *n (%)* | *n (%)* | *n (%)* | *n (%)* | *n (%)* | *n (%)* | *n (%)* | *n (%)* | *n (%)* | *n (%)* |
| | 3 | *n (%)* | *n (%)* | *n (%)* | *n (%)* | *n (%)* | *n (%)* | *n (%)* | *n (%)* | *n (%)* | *n (%)* | *n (%)* | *n (%)* |
| | 4 | *n (%)* | *n (%)* | *n (%)* | *n (%)* | *n (%)* | *n (%)* | *n (%)* | *n (%)* | *n (%)* | *n (%)* | *n (%)* | *n (%)* |
| | 5 | *n (%)* | *n (%)* | *n (%)* | *n (%)* | *n (%)* | *n (%)* | *n (%)* | *n (%)* | *n (%)* | *n (%)* | *n (%)* | *n (%)* |
| SOC + Fosfo | Normal | *n (%)* | *n (%)* | *n (%)* | *n (%)* | *n (%)* | *n (%)* | *n (%)* | *n (%)* | *n (%)* | *n (%)* | *n (%)* | *n (%)* |
| | 1 | *n (%)* | *n (%)* | *n (%)* | *n (%)* | *n (%)* | *n (%)* | *n (%)* | *n (%)* | *n (%)* | *n (%)* | *n (%)* | *n (%)* |
| | 2 | *n (%)* | *n (%)* | *n (%)* | *n (%)* | *n (%)* | *n (%)* | *n (%)* | *n (%)* | *n (%)* | *n (%)* | *n (%)* | *n (%)* |
| | 3 | *n (%)* | *n (%)* | *n (%)* | *n (%)* | *n (%)* | *n (%)* | *n (%)* | *n (%)* | *n (%)* | *n (%)* | *n (%)* | *n (%)* |
| | 4 | *n (%)* | *n (%)* | *n (%)* | *n (%)* | *n (%)* | *n (%)* | *n (%)* | *n (%)* | *n (%)* | *n (%)* | *n (%)* | *n (%)* |
| | 5 | *n (%)* | *n (%)* | *n (%)* | *n (%)* | *n (%)* | *n (%)* | *n (%)* | *n (%)* | *n (%)* | *n (%)* | *n (%)* | *n (%)* |

SOC=standard of care



Obiero CW, *et al. Arch Dis Child* 2022;0:1–9. doi: 10.1136/archdischild-2021-322483

*Table 26: Shift table for Alkaline Phosphatase*

| Baseline (Day 0) | | Day 2 | | | | | | Day 7 | | | | | |
|---|---|---|---|---|---|---|---|---|---|---|---|---|---|
| Grade | | Normal | 1 | 2 | 3 | 4 | 5 | Normal | 1 | 2 | 3 | 4 | 5 |
| SOC | Normal | *n (%)* | *n (%)* | *n (%)* | *n (%)* | *n (%)* | *n (%)* | *n (%)* | *n (%)* | *n (%)* | *n (%)* | *n (%)* | *n (%)* |
| | 1 | *n (%)* | *n (%)* | *n (%)* | *n (%)* | *n (%)* | *n (%)* | *n (%)* | *n (%)* | *n (%)* | *n (%)* | *n (%)* | *n (%)* |
| | 2 | *n (%)* | *n (%)* | *n (%)* | *n (%)* | *n (%)* | *n (%)* | *n (%)* | *n (%)* | *n (%)* | *n (%)* | *n (%)* | *n (%)* |
| | 3 | *n (%)* | *n (%)* | *n (%)* | *n (%)* | *n (%)* | *n (%)* | *n (%)* | *n (%)* | *n (%)* | *n (%)* | *n (%)* | *n (%)* |
| | 4 | *n (%)* | *n (%)* | *n (%)* | *n (%)* | *n (%)* | *n (%)* | *n (%)* | *n (%)* | *n (%)* | *n (%)* | *n (%)* | *n (%)* |
| | 5 | *n (%)* | *n (%)* | *n (%)* | *n (%)* | *n (%)* | *n (%)* | *n (%)* | *n (%)* | *n (%)* | *n (%)* | *n (%)* | *n (%)* |
| SOC + Fosfo | Normal | *n (%)* | *n (%)* | *n (%)* | *n (%)* | *n (%)* | *n (%)* | *n (%)* | *n (%)* | *n (%)* | *n (%)* | *n (%)* | *n (%)* |
| | 1 | *n (%)* | *n (%)* | *n (%)* | *n (%)* | *n (%)* | *n (%)* | *n (%)* | *n (%)* | *n (%)* | *n (%)* | *n (%)* | *n (%)* |
| | 2 | *n (%)* | *n (%)* | *n (%)* | *n (%)* | *n (%)* | *n (%)* | *n (%)* | *n (%)* | *n (%)* | *n (%)* | *n (%)* | *n (%)* |
| | 3 | *n (%)* | *n (%)* | *n (%)* | *n (%)* | *n (%)* | *n (%)* | *n (%)* | *n (%)* | *n (%)* | *n (%)* | *n (%)* | *n (%)* |
| | 4 | *n (%)* | *n (%)* | *n (%)* | *n (%)* | *n (%)* | *n (%)* | *n (%)* | *n (%)* | *n (%)* | *n (%)* | *n (%)* | *n (%)* |
| | 5 | *n (%)* | *n (%)* | *n (%)* | *n (%)* | *n (%)* | *n (%)* | *n (%)* | *n (%)* | *n (%)* | *n (%)* | *n (%)* | *n (%)* |

SOC=standard of care



Obiero CW, *et al. Arch Dis Child* 2022;0:1–9. doi: 10.1136/archdischild-2021-322483

| DNDi AFRICA Drugs for Neglected Diseases *initiative* | **Statistical Analysis Plan (SAP)** | **NEO_FOSFO_SAP_V0.1** | **18-Mar-19** |
|---|---|---|---|

*Table 27: Shift table for Creatinine*

| Baseline (Day 0) | | Day 2 | | | | | | Day 7 | | | | | |
|---|---|---|---|---|---|---|---|---|---|---|---|---|---|
| Grade | | Normal | 1 | 2 | 3 | 4 | 5 | Normal | 1 | 2 | 3 | 4 | 5 |
| SOC | Normal | *n (%)* | *n (%)* | *n (%)* | *n (%)* | *n (%)* | *n (%)* | *n (%)* | *n (%)* | *n (%)* | *n (%)* | *n (%)* | *n (%)* |
| | 1 | *n (%)* | *n (%)* | *n (%)* | *n (%)* | *n (%)* | *n (%)* | *n (%)* | *n (%)* | *n (%)* | *n (%)* | *n (%)* | *n (%)* |
| | 2 | *n (%)* | *n (%)* | *n (%)* | *n (%)* | *n (%)* | *n (%)* | *n (%)* | *n (%)* | *n (%)* | *n (%)* | *n (%)* | *n (%)* |
| | 3 | *n (%)* | *n (%)* | *n (%)* | *n (%)* | *n (%)* | *n (%)* | *n (%)* | *n (%)* | *n (%)* | *n (%)* | *n (%)* | *n (%)* |
| | 4 | *n (%)* | *n (%)* | *n (%)* | *n (%)* | *n (%)* | *n (%)* | *n (%)* | *n (%)* | *n (%)* | *n (%)* | *n (%)* | *n (%)* |
| | 5 | *n (%)* | *n (%)* | *n (%)* | *n (%)* | *n (%)* | *n (%)* | *n (%)* | *n (%)* | *n (%)* | *n (%)* | *n (%)* | *n (%)* |
| SOC + Fosfo | Normal | *n (%)* | *n (%)* | *n (%)* | *n (%)* | *n (%)* | *n (%)* | *n (%)* | *n (%)* | *n (%)* | *n (%)* | *n (%)* | *n (%)* |
| | 1 | *n (%)* | *n (%)* | *n (%)* | *n (%)* | *n (%)* | *n (%)* | *n (%)* | *n (%)* | *n (%)* | *n (%)* | *n (%)* | *n (%)* |
| | 2 | *n (%)* | *n (%)* | *n (%)* | *n (%)* | *n (%)* | *n (%)* | *n (%)* | *n (%)* | *n (%)* | *n (%)* | *n (%)* | *n (%)* |
| | 3 | *n (%)* | *n (%)* | *n (%)* | *n (%)* | *n (%)* | *n (%)* | *n (%)* | *n (%)* | *n (%)* | *n (%)* | *n (%)* | *n (%)* |
| | 4 | *n (%)* | *n (%)* | *n (%)* | *n (%)* | *n (%)* | *n (%)* | *n (%)* | *n (%)* | *n (%)* | *n (%)* | *n (%)* | *n (%)* |
| | 5 | *n (%)* | *n (%)* | *n (%)* | *n (%)* | *n (%)* | *n (%)* | *n (%)* | *n (%)* | *n (%)* | *n (%)* | *n (%)* | *n (%)* |

SOC=standard of care



Obiero CW, *et al. Arch Dis Child* 2022;0:1–9. doi: 10.1136/archdischild-2021-322483

A list of listings for additional assessments will be provided here including concomitant medication as well as individual patient data listings on;

i. adverse events
ii. Laboratories (Hematology, Biochemistry)
iii. Vital signs
iv. Physical exams
v. Demographics
vi. Protocol deviation

## References


1. Protocol: Safety and Pharmacokinetics of Fosfomycin in Hospitalised Neonates with Clinical Sepsis v2.0 dated 13th April 2018
