# Supplementary material for: Randomised controlled trial of fosfomycin in neonatal sepsis: pharmacokinetics and safety in relation to sodium overload
Source: Arch Dis Child. 2022 Jan 25;107(9):802–10. doi: 10.1136/archdischild-2021-322483 (PMC9411916; doi:10.1136/archdischild-2021-322483)
Supplement: Supplementary file 1 [file archdischild-2021-322483supp001.pdf]

Randomised Controlled Trial of Fosfomycin Safety and Pharmacokinetics in Neonatal Sepsis

Supplement 1

This supplement contains the following items:

1. Original protocol and final protocol
2. Informed consent forms

Intravenous and Oral Fosfomycin in Hospitalized Neonates with Clinical Sepsis: an open-label safety and pharmacokinetics study (neoFosfo)

Short title	Neo-Fosfo
Name of product(s)	Fosfomycin oral and IV formulations
Drug Class	Antibiotic
Indication	
Protocol Number	Neo-Fos-001
EudraCT	Not applicable
Study Sponsor	DND*i*<br>Tetezi Towers, 3rd Floor<br>George Padmore Rd<br>Kilimani<br>PO BOX 21936-00505<br>Nairobi<br>KENYA<br>+254 20 3995 000<br>Head Office: Chemin Louis Dunant, 15, 1202 GENEVA Switzerland Phone: +41 22 906 9230
Principal Investigator<br><br>Coordinating Institutions	Professor James A Berkley<br><br>KEMRI/Wellcome Trust Research Programme<br>PO Box 230, Kilifi, 80108, Kenya<br><br>University of Oxford, University Offices, Wellington Square, Oxford, OX1 2JD, UK

Clinical Trial Protocol Version / Date	Version 1.1 dated 23rd August 2017
Protocol Amendment Number / Date	Not applicable

The information contained in this document is confidential. It is to be used by investigators, potential investigators, consultants, or applicable independent ethics committees. It is understood that this information will not be disclosed to others without written authorization from DNDi, except where required by applicable local laws

CLINICAL TRIAL PROTOCOL SIGNATURE PAGE

Statistician

Signature	______________________	______________
		Date of Signature (DD/MMM/YY)
Name	Sarah Walker	
Title	Professor	
Institution	MRC Clinical Trials Unit	
Address	University College London, London, UK	

Principal Investigator

Investigator Signature	______________________	______________
		Date of Signature (DD/MMM/YY)
Name	Prof James A Berkely	
Title	Professor	
Institution	KEMRI/Wellcome Trust Research Programme	
Address	PO Box 230, Kilifi, 80108, Kenya	

Investigators Signature Page

I have read this protocol and agree that it contains all necessary details for carrying out this trial. I will conduct the trial as outlined herein and will complete the trial within the time designated.
I will provide copies of the protocol and all pertinent information to all individuals responsible to me who assist in the conduct of this trial. I will discuss this material with them to ensure they are fully informed regarding the drug and the conduct of the trial.
I will use only the informed consent form approved by the sponsor or its representative and will fulfil all responsibilities for submitting pertinent information to the Institutional Review Board/Independent Ethics Committee (IRB/IEC) responsible for this trial if required by national law.
I agree that the sponsor or its representatives shall have access to any source documents from which case report form information may have been generated.

Table of Contents

Title of the Project 7
Investigators and Institutional Affiliations 7
Lay summary 8
Abstract 10
Abbreviations 11
Introduction / Background 13
Justification for the study 17
State the Null Hypotheses 17
Objectives 17
a) General Objectives 17
b) Specific Objectives 17
Study Design 18
Study site: 18
Definition of Study Population: 18
Rationale for animal use and justification for animal species chosen 19
Sampling 19
i. Sample size determination: 19
i. For Pharmacokinetics: 19
Procedures: 20
Clinical Trial Governance: 25
Safety reporting 25
i) Assessment of Safety: 25
ii) Laboratory examinations 26
iii) Adverse event definitions and reporting 26
Withdrawal criteria: 30
Data Safety Monitoring Board: 30
Quality Assurance and Quality Control Procedures 31
A) Investigator's file: 31
B) Case report forms (CRFs): 31
C) Source documents: 31
E) Monitoring, audits and inspections: 31
F) Protocol Amendments: 32
Data Management and Analysis: .... **Error! Bookmark not defined.**
Data Sharing 32
Intellectual property 32
Time Frame/Duration of the Project 32
Ethical Considerations 33
Expected Application of the Results 35
References 36
Budget .... **Error! Bookmark not defined.**

Justification of the Budget..**Error! Bookmark not defined.**

Appendices ....**Error! Bookmark not defined.**

Appendix D: Further Information Regarding Study Treatment (Fosfomycin):Error! Bookmark not defined.

1. Mechanism of Action of IMP:..**Error! Bookmark not defined.**
2. Standard of Care treatment..**Error! Bookmark not defined.**
3. IMP dosing and treatment regimens:...**Error! Bookmark not defined.**
5. Storage..**Error! Bookmark not defined.**

Title of the Project

Intravenous and oral fosfomycin in hospitalized neonates with clinical sepsis: An open-label safety and pharmacokinetics study.

Investigators and Institutional Affiliations

KEMRI CGMR-C
James Berkley (PI)
Phoebe Williams
Christina Obiero
Neema Mturi
Claire Gordon
Joseph Waichungo
Johnstone Thitiri
Mwanamvua Boga
Sheila Murunga

KEMRI CCR
Martin Ongas

Non-KEMRI*:
Mike Sharland: St George’s Hospital, University of London, UK
Julia Anna Bielicki: St George’s Hospital, University of London, UK
Karin Kipper: St George’s Hospital, University of London, UK
Joseph Standing: University College, London, UK
Sarah Walker: Medical Research Council Clinical Trials Unit at University College London, UK

*Curriculum Vitae of non-KEMRI investigators attached, see appendix E.

<u>*Lay summary*</u>

A study measuring the levels of an additional antibiotic (fosfomycin) to treat severe infections in newborns.

What is the problem?
Among babies presenting with signs of serious infection, or who develop these signs whilst in hospital, bacterial resistance to the antibiotics that are normally used is increasing. This means that babies with infections may be at a higher risk of dying. In Africa, alternative antibiotics are often expensive and may themselves cause the bacteria to become resistant. Therefore, new treatment strategies are needed. Fosfomycin is a potentially inexpensive antibiotic that is licensed for use in children in Europe and may be useful to resource-poor countries. It has a good safety profile in children and is expected to be effective against infections that do not respond to the currently used antibiotics. However, more information is needed to inform how fosfomycin should be used in babies in Kenya.

What questions are we trying to answer?
We want to find out what doses of fosfomycin would be most suitable for sick newborns in Kenya in order to optimize its use in an effective and safe way. We also want to find out how well local bacteria that have been previously found (and stored) from patients at Kilifi County Hospital are able to be killed by fosfomycin.

Where is the study taking place?
The study will take place in Kilifi County Hospital, Kilifi, Kenya.

How many people does it involve?
For the study measuring the levels of antibiotics in newborn babies, we will include 120 babies admitted to the hospital with presumed infection. 60 of these babies will be given fosfomycin in addition to standard treatment and drug levels measured; the other 60 will receive standard treatment.

How are these people selected?
We will ask parents and guardians of all babies aged 28 days or less who are admitted to Kilifi County Hospital with signs of infection to participate, unless they are being transferred from another hospital, already received other antibiotics by injection, are very sick or premature, or have abnormally high salt (sodium) levels in their blood.

What does the study involve for those who are in it?
After providing information and answering any questions, informed consent will be requested from the child's parent or guardian. A doctor or study clinical officer will examine the baby and take the usual admission investigations, then prescribe the two antibiotics that are currently recommended by the WHO for the treatment of presumed infection in babies (ampicillin and gentamicin).

Half of the babies will be selected randomly to receive intravenous fosfomycin *as well as* the standard antibiotics. The nurse or clinical officer will then take two blood samples to check fosfomycin levels. After a minimum of four doses of intravenous (IV) fosfomycin (over 48 hours), when their condition is improved and they are tolerating feeds by mouth, the baby will then be changed on to oral fosfomycin. A further two blood samples will be collected after the first oral dose of fosfomycin, including one to check the kidney and liver function and level of salts in the blood (which is currently a routine investigation). Each of these blood samples is 0.5ml, giving a total for this research of 2.5ml (half a teaspoon) for checking the drug levels, and a further 1ml (a quarter of a teaspoon) for checking the level of salts in their blood.

For all babies, a blood test will be taken (which is normally part of routine care) at around 48 hours to check the blood count, kidney and liver function and level of salts in the blood. If a baby has a lumbar puncture as part of their normal treatment (if their doctor is concerned about an infection in their brain), the fosfomycin level in the fluid surrounding their brain will also be checked.

The babies will be closely followed by the study team, working together with the hospital staff to provide the best care available in the hospital. On day 7, any babies who remain as inpatients will have a blood test to check their kidney and liver function and level of salts in the blood (and 0.5ml drawn for fosfomycin levels in the group receiving this antibiotic). Breastfeeding and health counselling will be given according to national guidelines. All

babies will be followed up in our outpatients' clinic 28 days after their presentation to hospital and parents/guardians may also phone the study team directly on a study-specific mobile phone or bring the baby to the ward prior to that review, in case of health concerns.

What are the benefits and risks/costs of the study for those who are involved?

Additional staff (clinical officers and nurses) will be recruited to undertake study duties and assist in general care on the ward, adding to the staff available. Training will be enhanced for all paediatric ward staff on the treatment of babies presenting with infections, and on the prevention of infections within the hospitals. We will also make available additional antibiotics as needed, should a baby continue to have signs of infection despite treatment or remain unwell. Drawing a blood sample carries the potential risks of bruising to the vein or infection, and careful training on procedures will help to prevent these. There may be a small risk of the baby having high levels of salt (sodium) in their blood due to the salt content of the fosfomycin injection. Improved monitoring of kidney function and blood salt levels will offset these risks.

How will the study benefit society?

This study is leading up to a large clinical trial assessing how effective fosfomycin is to treat babies with infections, and if it is effective, will support efforts to make fosfomycin available at low cost for Kenya and other countries. This will help babies to be more effectively treated when bacteria are resistant to the currently used antibiotics.

When does the study start and finish?

The study aims to start as soon as scientific and ethical approval is granted and is expected to continue for 18 months (including analysis and write-up).

Abstract

Antimicrobial resistance (AMR) has become a major issue in global health. Despite progress in the reduction of under 5 mortality rates in recent decades, the proportion of neonatal deaths occurring within this age group has increased, with almost one quarter of all neonatal deaths occurring due to serious bacterial infection. Common bacteria causing neonatal sepsis are now exhibiting widespread resistance to several classes of antibiotics. There is an urgent need to discover new, effective treatments and re-evaluate existing therapeutic agents to treat infections potentially caused by multi-drug resistant (MDR) pathogens. Gram-negative bacteria (GNB) predominate as the cause of neonatal sepsis and are increasingly associated with high rates of resistance to the currently recommended WHO empirical therapy regimen of ampicillin/penicillin and gentamicin. There is therefore a need to develop an updated empiric regimen with improved efficacy in the context of increasing MDR sepsis in neonates. New antimicrobials under development will be expensive once licensed, and there are currently virtually no planned trials to assess their efficacy in neonates in low- and middle-income countries (LMICs).

One potential strategy is utilizing an existing off-patent (and therefore affordable) antibiotic available in intravenous and oral formulations – fosfomycin. Fosfomycin has a wide spectrum of activity against Gram-positive and Gram-negative bacteria causing neonatal sepsis. It is mainly used for resistant urinary tract infections in adults but has licensed neonatal and paediatric doses in Europe (though dosing regimens vary between countries). Both oral and IV formulations are available. A large clinical trial to assess the efficacy of a fosfomycin plus an aminoglycoside combination (compared to the current WHO recommended ampicillin and gentamicin) is anticipated, including sites in Kenya. The ultimate aim is for fosfomycin to be included in the WHO Essential Medicines List for children (EMLc) and be available for use in developing countries, where rates of resistance to ampicillin and gentamicin have been estimated at over 40%. The first steps before this trial are to clarify the pharmacokinetics (PK) and safety profile of fosfomycin in neonates, as well as generating further information regarding local patterns of bacterial susceptibility to fosfomycin. The aim of this study is to fulfil both these steps. Fosfomycin (IV and oral) PK will be investigated among 60 babies admitted to hospital and being treated for presumed sepsis; administered alongside the standard antibiotics. Another 60 babies receiving standard treatment only (without PK sampling) will be monitored in the same way to compare adverse events. In the laboratory at CGMR-C, previously archived bacterial isolates will be tested for their sensitivity to fosfomycin.

Abbreviations

AE	Adverse Event
AGISAR	WHO Advisory Group on Integrated Surveillance of Antimicrobial Resistance
AMR	Antimicrobial Resistance
BSAC	British Society of Antimicrobial Chemotherapy
BW	Birth weight
C_{max}	Peak serum concentration of a therapeutic drug
CBC	Complete blood count
CGMR-C	Centre for geographic medicine research, Coast (Kenya)
CI	Confidence Interval
CLSI	Clinical and Laboratory Standards Institute
CMP	Calcium, Magnesium and Phosphate
CNS	Central Nervous System
CR	Carbapenem resistance/resistant
CRF	Case report form
CRO	Carbapenem resistant organisms
CSF	Cerebrospinal Fluid
DSMB	Drug Safety Monitoring Board
ECCMID	European Congress of Clinical Microbiology and Infectious Diseases
EMLc	Essential Medicines List for children
ESBL	Extended Spectrum Beta-Lactamase
ESPGHAN	European Society for Paediatric Gastroenterology, Hepatology and Nutrition
EUCAST	European Committee on Antimicrobial Susceptibility Testing
FBC	Full blood count
GNB	Gram-negative bacteria
HIE	Hypoxic Ischaemic Encephalopathy
ICF	Informed consent form
IMP	Investigational Medicinal Product
IV	Intravenous
IP	Intellectual Property
LBW	Low birth weight
LMICs	Low- and middle-income countries
LC-MS	Liquid chromatography mass spectrometry
LSM	Local Safety Monitor

MDR	Multi-drug resistant
MIC	Minimum inhibitory concentration
MRSA	Multi-resistant *Staphylococcus Aureus*
OxTREC	Oxford University Tropical Research Ethics Committee
PK	Pharmacokinetic
PO	Per os (oral)
PPB	Republic of Kenya Ministry of Health Poisons and Pharmacy Board
SAE	Serious Adverse Event
SBI	Serious bacterial infection
SERU	Scientific and Ethics Review Committee (Kenya)
SIADH	Syndrome of inappropriate antidiuretic hormone secretion
SOC	Standard-of-care
SOP	Standard operating procedure
SPC	Summary of Product Characteristics
SUSAR	Suspected Unexpected Serious Adverse Reaction
UTI	Urinary tract infection
VRE	Vancomycin-resistant enterococci
WHO	World Health Organization

Introduction / Background

The purpose of this study is to support the design of an international multi-centre randomized trial of fosfomycin to treat neonates with presumed sepsis, by providing an improved understanding of fosfomycin pharmacokinetics, safety and antimicrobial susceptibility of local invasive bacterial species.

Maternal and child deaths have halved over the past two decades;[1] however neonatal mortality has remained unacceptably high, with an estimated 2.9 million deaths in newborns every year.[2] The proportion of deaths (in children under 5 years) occurring in the neonatal period has increased from 38% to 44% between 2000 and 2013,[2] and 23% of neonatal deaths are due to presumed serious bacterial infections (SBI).[1] Aside from this mortality burden, sepsis in the neonatal period is also associated with significant morbidity secondary to an increased risk of adverse neurodevelopmental outcomes.[3]

The WHO and Kenyan guidelines currently recommend ampicillin (or penicillin) plus gentamicin for the treatment of sepsis in neonates and infants <2 months of old, with third-generation cephalosporins listed as second-line therapy.[4] However, two recent systematic reviews have documented increasing rates of AMR to this regimen.[5,6] Downie *et al*. (2013) examined 19 studies from 13 LMICs across Asia and Africa, revealing non-susceptibility to penicillin/gentamicin and third-generation cephalosporins of 44% and 43% respectively.[5] Le Doare *et al.* (2015) identified 15 studies investigating non-susceptibility among Gram-negative pathogens across SE Asia, Africa and the Middle East which revealed *Enterobacteriaceae* exhibit high rates of non-susceptibility to ampicillin (80%), gentamicin, (22%) and ceftriaxone (74%).[6]

Challenges in interpreting this literature include the limited data available being mostly from urban tertiary hospital settings (rather than district- or community-level facilities), failure to account for prior treatment, not distinguishing community- from hospital-acquired infections, and inconsistent laboratory facilities. Nevertheless a consensus is emerging that AMR to recommended first-line antibiotics in LMICs is associated with significant morbidity and mortality.[7,8] A recent study of neonatal deaths attributable to MDR sepsis (in 5 countries accounting for half the global neonatal sepsis death rates - India, Pakistan, Nigeria, DR Congo and China) identified 214,000 neonatal deaths occurring each year due to resistant bacterial infections.[9] Notable is the emergence and spread of extended spectrum beta-lactamase (ESBL)-producing *Enterobacteriaceae*, which render many commonly used (and cheaply available) antimicrobials ineffective. Carbapenems are increasingly being used as second-line therapy in neonatal sepsis, but they are expensive, and their use is associated with increasing AMR due to the dissemination of infections with carbapenem-resistant organisms (CRO). There is therefore a need to clarify an empiric regimen with improved for use in LMICs.

The repurposing of older antimicrobials for current treatment regimens has recently received increasing international attention. The WHO Advisory Group on Integrated Surveillance of Antimicrobial Resistance (AGISAR) included fosfomycin in the current revision of critically important antimicrobials for human medicine.[10] Fosfomycin is a bactericidal peptidoglycan antibiotic that was first produced in the 1970s[11] though its marketing was largely replaced in the 1980s by oral cephalosporins. Its infrequent international use over the past 30 years has resulted in low global resistance rates.

Fosfomycin is orally absorbed, crosses the blood brain barrier and is renally excreted. It exhibits minimal toxicity, low levels of cross-resistance, and provides synergistic effects with other antibiotics (including beta-lactams, aminoglycosides and fluoroquinolones).[12] IV fosfomycin is licensed in Europe and the USA as a second-line treatment in adults and children with osteomyelitis, complicated UTI, nosocomial lower respiratory tract infections, bacterial meningitis, or bacteraemia associated with any of these causes. Oral fosfomycin is used for treating UTI caused by *Escherichia coli* and *Enterococcus faecalis*.

Fosfomycin has a broad-spectrum of activity against both Gram-negative and Gram-positive organisms, including MRSA and ESBL infections.[13] A recent systematic review evaluated the susceptibility of contemporary bacteria to fosfomycin, revealing 84 studies which documented susceptibility to *Staphylococcus aureus* (range 33% to 100%); ESBL-producing *Escherichia coli* (range 81% to 100%); ESBL-producing *Klebsiella pneumoniae* (range 15% to 100%); and carbapenem-resistant *Klebsiella pneumoniae* (range 39% to 100%).[14] Thus, fosfomycin currently exhibits high levels of antimicrobial activity against common causes of neonatal sepsis.

The Summary of Product Characteristics (SPC) gives a neonatal intravenous dosing, including for preterm and term infants by age and body weight (Table 1). However, parenteral dosing recommendations for neonates and children patients vary widely between countries in Europe (Table 2), and there are currently no PO dosing recommendations for neonates.

Age/weight	Daily dose
Premature neonates (age [a] < 40 weeks)	100 mg/kg BW in 2 divided doses
Neonates (age [a] 40-44 weeks)	200 mg/kg BWin 3 divided doses
Infants 1-12 months (up to 10 kg BW)	200-300 [b] mg/kg BW in 3 divided doses
Infants and children aged 1-12 years (10-40 kg BW)	200-400 [b] mg/kg BW in 3-4 divided doses

[a] Sum of gestational and postnatal age.

[b] The high-dose regimen may be considered for severe infections and or serious infections (such as meningitis), in particular when known or suspected to be caused by organisms with moderate susceptibility.

Table 1. Parenteral Fosfomycin Neonatal Dosing Recommendations (Nordic Pharma, 2016)

Country	Neonates (pre- & full-term; 0-1 months)	Infants (1-12 months, up to 10kg)	Children (1-12 years; 10-40kg)
Austria	100-200/400* mg/day in 2-3 doses	100-200/400* mg/day in 2-3 doses	4-8g/day, in 2-3 doses
Germany	100mg/day in 2 doses	200-250mg/day in 3 doses	100-200/300*mg/day in 3 doses
United Kingdom	Premature: 100mg/kg in 2 doses<br>Term: 200mg/kg in 3 doses	200-300mg/kg/day in 3 doses	200-400mg/kg in 3-4 doses
Spain	Not specified	Not specified	100-200/400mg/day in 2-3 doses
France	Not specified	Not specified	100-200mg/day; number of daily doses not specified

*Maximum dosage for severe infections

Table 2: Recommended total daily dosages for IV fosfomycin in paediatric patients with normal renal function across various European settings

Safety and Clinical Outcomes of Fosfomycin:

Five published papers have documented the clinical outcomes of (n=84) neonates treated with parenteral

fosfomycin therapy for a range of diagnostic situations (Table 3), with no deaths or severe adverse events attributed to this therapy.

A 2015 review of adverse events (AE) reported to the FDA and the international literature in association with fosfomycin administration (in both adult and paediatric patients) concluded that fosfomycin exhibits low toxicity and few concerns regarding its safety profile.[15] This review included data assessing 254 paediatric patients across 6 trials (3 trials of parenteral and 3 of oral fosfomycin; age range: neonates – 15.5 years), 3 of which were retrospective (n=118)[16–18] and 3 prospective randomized trials (n=134)[19–21] investigating oral fosfomycin. In the trials of parenteral fosfomycin, the drug was administered for up to 4 weeks for the treatment of acute hematogenous osteomyelitis, bacteraemia, and lung infection; while oral fosfomycin was administered as a single dose for the treatment of UTI. Overall, no serious safety issues related to the use of fosfomycin in children were identified in this review; with the most frequently reported AEs associated with (IV and PO) administration across all age ranges identified as being rash, peripheral phlebitis and gastrointestinal symptoms. Less common AEs include hypersensitivity and abnormal liver function. These are common AEs which also occur with other antibiotics.

Combined with the 31 babies documented in the literature investigating fosfomycin PK data (discussed below), this results in a total of 367 children in whom fosfomycin has been administered in the published literature with no significant safety concerns having been reported in this cohort.

However, an important potential safety consideration for parenteral fosfomycin is the sodium (Na+) content (14.4mmol/330mg sodium per gram). The European Society for Paediatric Gastroenterology and Hepatology (ESPGHAN) recommends a daily (enteral) sodium intake of 69mg/kg (minimum) to 115mg/kg (maximum) for preterm infants (with enteral values for term infants not published),[22] and a parenteral sodium intake of 2-3mmol/kg/day for term neonates and 3-5mmol/kg/day for premature neonates.[23] Fosfomycin's sodium content equates to a sodium load of 2.8mmol/kg/day (based on dosing of 200mg/kg/day), which is within the published guidelines for neonates. There are negligible amounts of sodium in IV ampicillin and gentamicin, the antibiotics alongside which fosfomycin will be administered; and there is no sodium in the oral fosfomycin formulation.

The ability to reabsorb sodium is inversely proportional to gestational age, and nephrogenesis is complete by 34 weeks gestation.[24] Hence, we aim to restrict our patient population to exclude very preterm infants. Hypernatremia may also occur secondary to hypoxic-ischemic encephalopathy (i.e., as a consequence of asphyxiation, due to central diabetes insipidus or via acute renal injury). Therefore, any baby presenting with seizures or with admission sodium ≥150mmol/L or creatinine ≥150micromol/L will be excluded from the study. All poorly feeding babies will receive IV (10% dextrose) fluids (as per Kenyan Paediatric Protocols).

Of note, the oral fosfomycin suspension contains no sodium, using a calcium base at a dose equivalent to 1.4mmol/kg/day, within the published neonatal guidelines for calcium administration (of 1.3-3mmol/kg/day).[23] Monitoring of calcium, magnesium and phosphate will therefore be undertaken. Oral fosfomycin also contains fructose to the equivalent of 1600mg/kg/day. There is little published research regarding high fructose loads in neonates, with most previous trials documenting safety at lower doses (150mg as an analgesic therapy);[25] while a recent meta-analysis evaluating sucrose administration (in 7,049 infants) documented a "very low" incidence of minor adverse events, with no reported major adverse events.[26] Nonetheless, the possible adverse event of osmotic diarrhoea will therefore be closely monitored in this study.

Study	N (Total n=82)	Dose and clinical setting	Clinical Setting	Outcomes
Taylor et al. 1977[27]	43 neonates	150-200mg/kg/day	Enterocolitis caused by enteropathic *E. coli*	Favourable clinical outcome in 88%
Rossignol & Regnier 1984[28]	21 neonates	200mg/kg/day in two divided doses, in combination with gentamicin/tobramycin	Sepsis and UTI	Clinical recovery in 19/21 (90.5%)
Guillois et al. 1989[29]	Case report (n =1)	IV fosfomycin-vancomycin, followed by oral pristinamycin	MSSA septicaemia with a liver abscess	Full recovery
Gouyon et al. 1990[30]	16 neonates	IV fosfomycin-cefotaxime	*Staphylococcal* septicaemia (*epidermidis* (n=10) and *aureus* (n=6)) (including meningitis and osteomyelitis)	Full recovery in n=15 (94%)
Algubaisi et al. 2015[31]	Case report (n=1 term infant)	120mg/kg/day fosfomycin and meropenem	Multiple *Citrobacter koseri* intracerebral abscesses	Clinical recovery

Table 3: Clinical studies describing the use of fosfomycin in neonatal sepsis. Modified from Li et al (in publication)[32]

Documented Pharmacokinetics of Fosfomycin:

A recent review of the PK profile of fosfomycin in neonates identified four small additional published studies assessing IV fosfomycin (with no oral PK data available) (Table 4). The elimination half-life (t½) of fosfomycin ranged from 2.4-7 hours following an IV bolus of 25-50mg/kg administered to neonates which included LBW and premature infants.[33,34] Fosfomycin is almost completely eliminated by glomerular filtration, with 80-95% of the dose unchanged in the urine within 24 hours.[35] Consequently, neonates have a prolonged fosfomycin t½ compared to older children and adults due to immature glomerular filtration and a greater volume of distribution.[34] Serum protein binding of fosfomycin has been estimated to be below 3%, and the neonatal C_{max} (60-90mg/L) is comparable with adult populations.[35,36]

Study	N (Total n=31)	Dose and study	Outcome
Molina et al. 1977[34]	11 neonates	50mg/kg IV, comparing infants 1-3d old and 3-4 weeks old	Elimination slower at earlier corrected gestational age
Guggenbichler 1978[33]	5 term & 5 pre-term neonates	25mg/kg IV	95-98% recovered in the urine, 1 compartment model

Study	N<br><br>(Total n=31)	Dose and study	Outcome
Guibert et al.<br>1987[37]	10 neonates	200mg/kg BD, comparing 30m or 2hr infusion schedules	No difference between schedules, serum concentrations are above MIC of common pathogens at 12h post dose
Suzuki et al.<br>2009[38]	Not identified	Dose estimation for renally excreted drugs	Dose estimation validated with GFR, tubular secretion clearance and fraction of unbound drug in plasma

Table 4: Neonatal fosfomycin pharmacokinetic studies; modified from Li et al (2016; in publication)

Bactericidal effects correlate with time above the MIC (t>MIC).[39] Pharmacokinetic modelling suggests that the current lower recommended paediatric doses (100mg/kg/day, Table 2 & Table 3) are insufficient for achieving target t>MIC for term neonates; and the corrected gestational age and body weight in neonates are the key explanatory variables for fosfomycin's PK.[39]

Previous research investigating the oral bioavailability of fosfomycin in adults documented a range between 34 and 58%.[40,41] Absorption is via the small intestine and is reduced by concurrent administration with food (37% when fasting versus 30% with food), thus, C_{max} that is higher under fasting conditions.[42]

<u>*Justification for the study*</u>

Neonatal sepsis has a high risk of morbidity and mortality. The current WHO and national guidelines recommend antibiotics to which resistance is reported in neonatal populations, although the available data is limited. Research on alternative empirical regimens for neonatal sepsis which are affordable, safe and cost-effective, with a step-down oral option, is needed. AMR is an issue of global public health concern and is one of the WHO's global health priority areas.[43] Understanding the benefits, risks, MIC capacity and PK of fosfomycin will influence global policy on the case management of neonates with sepsis in Kenya and international settings.

<u>*State the Null Hypotheses*</u>

i) The pharmacokinetics of the currently recommended various doses of IV and PO fosfomycin are unsuitable for treating neonates.
ii) Fosfomycin administration is not associated with altered plasma sodium in neonates.
iii) Fosfomycin does not inhibit growth of more than 25% of archived isolates of *Enterobacteriaceae* that express an ESBL phenotype *in vitro.*

<u>*Objectives*</u>

a) General Objectives

To improve the understanding of fosfomycin pharmacokinetics and safety amongst newborns aged ≤28 days hospitalized with clinical sepsis and provide detailed information regarding the antimicrobial susceptibility of local invasive bacteria to fosfomycin.

b) Specific Objectives

- To estimate the PK disposition parameters of IV and PO fosfomycin in neonates
- To assess the safety of fosfomycin, particularly with regard to possible elevation of sodium after 48 hours of IV fosfomycin administration in neonates
- To estimate the oral bioavailability of fosfomycin in neonates
- To generate preliminary data on the safety of oral fosfomycin in neonates

- With the above information, generate a recommended dosing schedule for future IV and PO fosfomycin efficacy trials

c) Secondary Objective
- To gain information regarding susceptibility patterns of local bacterial species to fosfomycin

Study Design
A safety and pharmacokinetic study among neonates admitted to a rural hospital in Kenya and eligible for IV antibiotics under current national guidelines. 120 patients will be randomized 1:1 to standard-of-care antibiotics *plus* a 7-day course of fosfomycin (n=60); or standard-of-care (n=60) antibiotics only (ampicillin 50mg/kg twice daily and gentamicin [3mg/kg for babies <2kg or 5mg/kg for babies >2kg] once daily for 7 days, as per Kenyan guidelines).

For the group receiving fosfomycin, fosfomycin will initially be administered IV for at least 48 hours together with standard care (ampicillin + gentamicin). Then, once babies are tolerating oral feeds and clinically improved, fosfomycin will be changed to oral administration to complete a total of 7 days of fosfomycin (or until the baby is discharged). Two PK samples will be taken after each of the first IV and oral doses, with sampling times allocated within possible early (0 to 4h) and late (4 to 12h) time-points after starting the IV and PO formulations; then again together with biochemistry after 7 days for those babies whom remain as inpatients. In total, four PK blood samples of 0.5ml each will be drawn from each participant, plus a fifth sample collected at 7 days to check electrolytes, and from which a PK sample will be assessed from any residual blood. Biochemistry (a commonly performed investigation for babies with sepsis) will be checked at 48 hours and 7 days for participants in both groups at the same time that the PK sample is collected. Daily observations until discharge and AEs up to a follow-up visit at day 28 will be recorded.

For the group receiving standard-of-care only, daily observations until discharge and AEs up to a follow-up visit at day 28 will be recorded.

Study site:
Kilifi County Hospital, Kilifi, Kenya

Definition of Study Population:
a) Criteria for inclusion of subjects (for pharmacokinetics):
Neonates defined as:

- Age 0 to 28 days inclusive
- Weight >1500g
- Born (an estimated) >34 weeks gestation (calculated as per the Ballard Maturational Assessment)
- Admitted to hospital and eligible to receive IV antibiotics, according to national guidelines

All neonates presenting to Kilifi County Hospital meeting the above criteria will be screened for inclusion in a systematic recruitment process.

b) Criteria for exclusion of subjects
- Baseline sodium level ≥150mmol/L
- Baseline creatinine ≥150 micromol/L
  Presenting with severe (grade 3) Hypoxic Ischemic Encephalopathy (HIE), defined as per Sarnat and Sarnat[44] as a stuporous, flaccid infant (with or without seizure activity) with suppressed brainstem and autonomic functions and absent reflexes
- Requiring cardiopulmonary resuscitation on admission

- Jaundice requiring exchange transfusion
- Admitted as a transfer after an overnight inpatient stay at another hospital
- Known allergy or contraindication to fosfomycin
- A specific clinical indication for another class of antibiotic (other than the nationally recommended standard-of-care)
- More than 4 hours after initiating ampicillin plus gentamicin (one dose), which allows for administration of these first-line antibiotics not to be delayed by study procedures
- Concurrent participation in another clinical trial
- Attending clinician's judgement that the child is so severely ill that adequate communication about the study with the parent or legal guardian is not possible.
- Not planning to remain resident in the County for the next 28 days.
- Lack of consent

Rationale for animal use and justification for animal species chosen

Not applicable

Sampling

i. Sample size determination:

i. For Pharmacokinetics:

Sample size has been calculated to ensure that the following PK parameters can be estimated with sufficient precision such that a dose schedule can be recommended for a future efficacy trial:

- Clearance (CL)
- Central volume (V)
- Oral Bioavailability (F)

Precision limits were set to 20%, and the power to estimate parameters with 95% confidence intervals within these limits was assessed by simulation-estimation. The simulation model consisted of an adult disposition model, with age and size scaling down to neonates, with added first-order absorption and assumed bioavailability. Six sampling time points were chosen to cover the dose intervals (3 early, 3 late) and the simulated population was randomly assigned age and weight combinations across the range expected for neonates. Parallel and crossover (IV/oral) designs were considered, with a range of 2-4 samples per patient. Power for sample size was greatest for the cross-over design. For the cross-over design, a minimum of 45 subjects contributing the complete set of 4 samples each (allocated early and late sample following the first IV and PO dose) are required to provide power of >85% to estimate all parameters within 20% precision limits. We estimate that up to 25% of subjects will not provide complete sample sets (either due to missing samples or withdrawal), so plan to recruit 60 subjects to ensure 45 complete sample sets. If all 60 subjects provide complete sample sets, power would rise to 96%.

ii. For Plasma Sodium

We have reviewed the data of (n=1,785) neonates >1500g admitted to Kilifi County Hospital (2015/6), which indicate a sodium mean and standard deviation of 139mmol/L and (SD 7.6, range 106 to 198mmol/L). 7.4% of babies had an admission sodium of >150mmol/L (our exclusion criterion). Excluding these babies, the mean sodium level in (the remaining n=1,653) babies was 137mmol/L (SD 5.2). With a minimum of 45 in each group (PK versus standard-of-care), the study has >85% power to detect a difference in sodium of 5mmol/L between groups.

The sample size is not intended to be powered for antimicrobial efficacy or clinical outcomes.

iii. For MIC of stored bacterial isolates and bowel flora:

Susceptibility of fosfomycin and other antibiotics is already being tested as per protocol SSC-1433. We will test n=200 invasive isolates from paediatric patients collected within the last 5 years, calculated based on >80% power

to discriminate a non-susceptible proportion of up to 17% from a hypothetical proportion of 25% (one-sided). This is selected as a proportion which would render fosfomycin ineffective for introduction should this level of non-susceptibility be found. We shall then investigate fosfomycin susceptibility on other (ESBL-negative) Gram-negative isolates, and Gram-positive pathogens. For assessment of susceptibility patterns in bowel flora, we will systematically assess all admission and discharge nappy swabs from those babies included in the study.

ii. Study Endpoints:

1. Primary Endpoint:

Estimation of the pharmacokinetic disposition and absorption parameters of IV and oral fosfomycin in neonates with clinical sepsis with sufficient precision such that a dose schedule can be recommended for a future efficacy trial

2. Secondary Endpoint(s):

- Difference between the groups in mean 48-hour plasma sodium concentrations
- Difference between the groups in mean 7-day plasma sodium concentrations
- Difference between groups in the rate of adverse events (any grade) to 28 days after enrolment in the study.

Procedures:

A) Analysis of Bacterial Isolates:

Isolates collected from nappy swabs will be subcultured and tested for fosfomycin susceptibility using disk diffusion (*E. coli*) and agar dilution (all isolates).[45] For disc diffusion, commercially available discs containing 200ug fosfomycin and 50mg of glucose-6-phostpate will be used. MICs will be determined by the agar dilution method using Mueller-Hinton agar supplemented with 25ug/m of glucose-6-phosphate and doubling concentrations of fosfomycin. The MIC will be recorded as the lowest concentration inhibiting visible growth. Plates will be incubated in ambient air at 35°C for 16 to 18 hours. Testing will be performed in duplicate, and mean MICs / zone diameters interpreted using EUCAST breakpoints (http://www.eucast.org/clinical_breakpoints/).

B) Pharmacokinetic Study - Enrolment Procedure:

All neonates presenting to Kilifi County Hospital will be systematically screened to assess their eligibility in meeting the inclusion criteria and consent requested from the parent / guardian. Sequential study numbers will be generated according to a blocked randomization from a list of random block sizes created before the study begins. Randomization cards linking allocation (to standard care plus fosfomycin or standard care alone) to study number will be placed in sealed opaque envelopes by the study sponsor. On enrolment, infants will be allocated study numbers sequentially, thus randomly allocating the two groups. Since this is an open-label study, once an envelope is opened, the randomization card will be securely attached to the patient's CRF.

a) Consent Process

Consent will be required for all data and samples taken for research purposes. Consenting will be done in a private room by study clinicians or trained field assistants, with the opportunity to ask questions and discuss concerns. Informed consent will be administered in a language that the parent/guardian best understands (English, Swahili or Giriama) after assessment of his/her literacy level. This will be done in the paediatric ward or high dependency unit once the decision to admit has been made. Whilst giving written consent parents/legal guardians will be able to agree to consent separately for participation in the study, storage of data and samples for future research, and export of samples for the PK assay that cannot currently be conducted in Kenya.

b) Data Collection

For all participants, a study-specific case report form (CRF) will be used from the time of enrolment and captured information will be entered into a database. The CRF will include a daily standardized record of clinical progress and drugs administered which will also be entered into a database. At discharge, the date, vital status and weight will be recorded.

c) *Data Management and Analysis:*

After the CRF has been completed and monitored by the clinical monitor, CRFs will be collected and data will be entered onto a validated password protected Openclinica database. Data will be kept confidential, with access restricted on password-protected computers, with regular secure backup. Any data transferred between Kenya and Europe will be emailed within password-protected encrypted files.

Analysis of fosfomycin and major metabolite concentration in plasma will be undertaken by Liquid Chromatography Mass Spectrometry (LC-MS) using validated methods in the GCP/GLP compliant laboratory in Analytical Services International Ltd, St George's Hospital, London, UK. Where possible, (scavenged) PK for penicillin and gentamicin will be measured using the same sample. Analysis will undertake by Dr Karin Kipper, Dr Joe Standing and Mr Martin Ongas, who will be trained on the techniques whilst running the analyses.

PK modelling and dosing simulations will be undertaken by non-linear mixed-effects modelling using NONMEM® software. The volume of distribution, half-life, clearance and trough levels of bound and unbound drug, and active metabolites will be estimated with 95% confidence intervals. Periods with concentrations above the CLSI, EUCAST and BSAC susceptibility breakpoints will be estimated. We will examine the effects of covariates including age, weight, and concurrently measured plasma sodium, potassium, and liver enzymes. Monte-Carlo simulations will be performed to determine the appropriate dosage and frequency of administration.

d) Clinical Care

Alongside protocol specific training, the study team will also conduct refresher training for clinicians on the current national guidelines for managing neonates presenting with presumed sepsis. Neonates will be reviewed every day by study clinicians, working together with the hospital team. All adverse events will be documented and reported, with any grade 4 or SAE reported within 48 hours to an independent DSMB. All other aspects of care will be provided according to national guidelines. Should a patient require second-line antimicrobials (third-generation cephalosporins), they will not be removed from the study as this will not impact the fosfomycin PK data.

e) Study Treatment:

Fosfomycin is a peptidoglycan antibiotic which has bactericidal effects. There will be two formulations of fosfomycin utilized in this study:

- Fosfomycin 40 mg/ml powder for solution for infusion
- Fosfomycin powder for reconstituted suspension (250mg/5ml)

Preparation will be in accordance with manufacturer's instructions. Further details regarding treatment dispensing, administration and accountability is documented in Appendix D. Training will be provided to all staff involved in its administration.

f) Timing of Assessments:

A schedule of events identifying the timing of required assessments and investigations is documented in Figure 2:

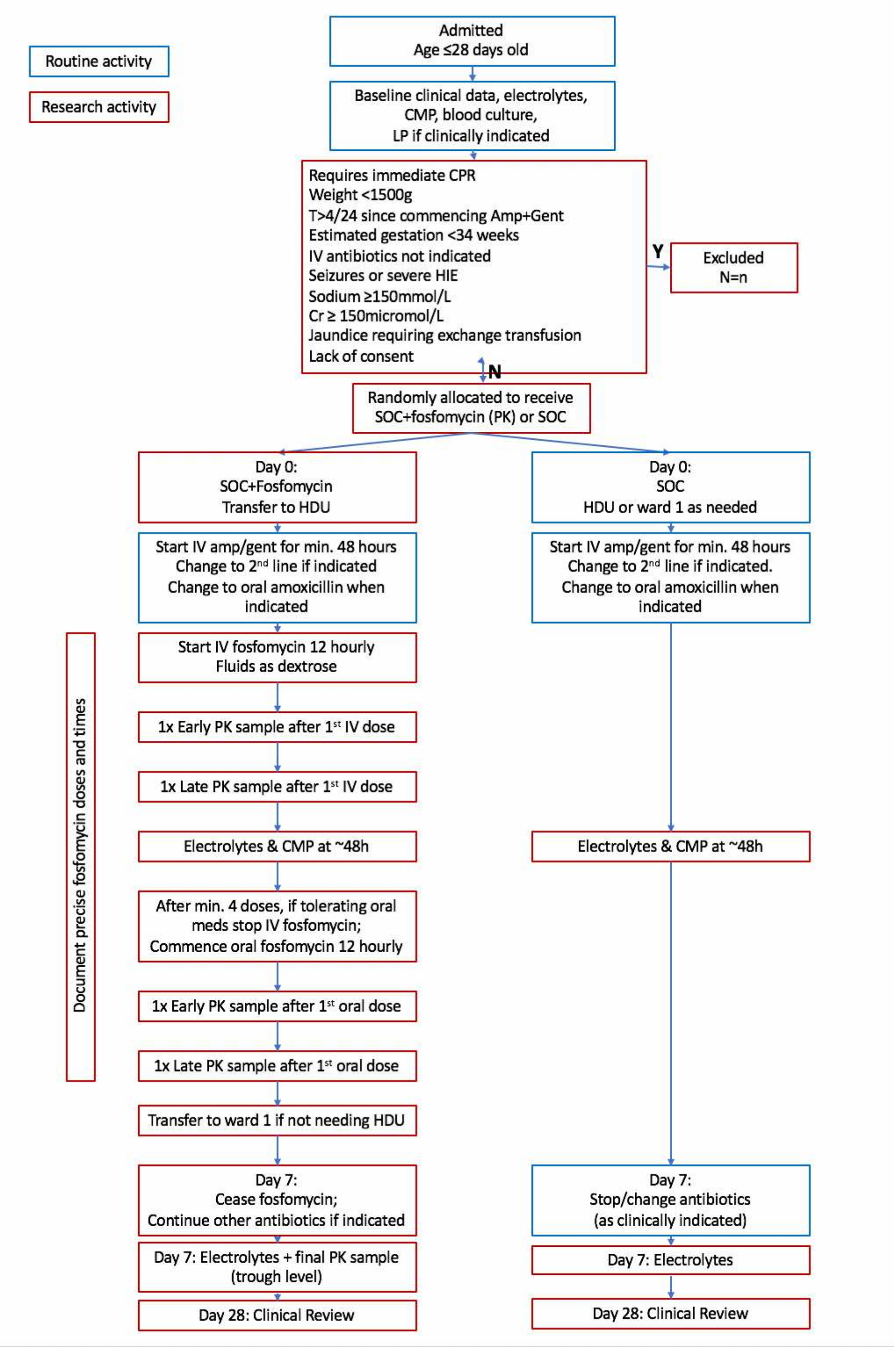

Figure 2: Schedule of Events

Note: If a Lumbar Puncture is clinically indicated after commencing fosfomycin, a scavenged PK sample will be obtained from the CSF

g) Pharmacokinetics Procedures:

Baseline Assessments:

Following informed consent, study clinical officers will prescribe both routine standard-of-care antibiotics (ampicillin 50mg/kg twice daily, and gentamicin [3mg/kg for babies <2kg, 5mg/kg for babies >2kg] once daily) and, for the PK group, fosfomycin (100mg/kg every 12 hours, initially IV). Findings from history and examination, and standard admission investigations (CBC and biochemistry) will be collected at baseline. A blood culture will be performed at admission +/- lumbar puncture; from which a scavenged PK sample will be sent for analysis if sufficient CSF remains (if there is a clinical indication for this to occur following the administration of IV fosfomycin). In order to assess antimicrobial resistance that is bought into hospital and that which has been acquired on the ward, an antimicrobial susceptibility profile will be determined for rectal carriage of resistant isolates by collecting a nappy swab at admission and discharge. This will enable determination of the effect of carriage of antimicrobial resistance following treatment with fosfomycin.

Pharmacokinetic Assessments:

The first dose of fosfomycin will be followed by the collection of two PK samples at allocated times: one early (during 0 to 4 hours post-dose) and one late (during 4 to 12 hours post-dose). After a minimum of 48 hours (or 4 IV doses), when tolerating oral medications, fosfomycin will be changed to oral and prescribed at the same dose (100 mg/kg every 12 hours). Following the first oral dose, one early and one late PK sample will again be obtained. For those who remain as inpatients, a PK sample of 0.5ml will be obtained together with a day 7 biochemistry. This will involve 5 plasma PK samples in total per patient, estimated as requiring an upper limit of 0.5 mL/sample (resulting in 2.5mL total study PK sample collection).

As per usual clinical procedures, blood for plasma electrolytes will be drawn at ~48 hours (co-ordinated with the first post-oral PK sample time-point for patients who step-down to oral fosfomycin at this point) and again at 7 days (for those who remain as inpatients in each study group).

All participants will receive a structured daily review including clinical status and current treatment. Standard antibiotics may be altered in line with the results of a blood culture, which is currently done routinely for clinical care (fosfomycin will be continued). Planned follow-up for clinical review will be done at day 28. Participant fares and compensation for lost work time will be provided at standard rates for this visit. Participants may also attend the ward in case of significant illness between discharge and day 28.

Drug dose or sample	Target times
Dose: 1st Ampicillin	After admission investigations
Dose: 1st Gentamicin	After admission investigations
Dose: 1st Fosfomycin IV	After admission investigations
SAMPLE 1*: ONLY ONE OF:	
5 minutes	5 minutes after 1st fosfomycin intravenous dose
30 minutes	30 minutes after 1st fosfomycin intravenous dose
60 minutes	60 minutes after 1st fosfomycin intravenous dose
SAMPLE 2: ONLY ONE OF:	
2 hours	2 hours after 1st fosfomycin intravenous dose
4 hours	4 hours after 1st fosfomycin intravenous dose
8 hours	8 hours after 1st fosfomycin intravenous dose
Dose: 2nd Ampicillin	12 hours after 1st ampicillin dose; *continuing 12 hourly*
Dose: 2nd Fosfomycin IV	12 hours after 1st fosfomycin dose
Dose: 2nd Gentamicin	24 hours after 1st Gentamicin dose; *continuing daily*
Dose: 3rd Fosfomcycin IV	24 hours after 1st fosfomycin dose
Dose: 4th Fosfomycin IV	36 hours after 1st fosfomycin dose
--Change to PO fosfomycin-- 1st dose Fosfomycin PO	48 hours after 1st fosfomycin dose; when tolerating oral medication and without signs of sepsis (otherwise continue IV 12 hourly)
SAMPLE 3: ONLY ONE OF: (collected alongside biochemistry)	
5 minutes	5 minutes after 1st fosfomycin oral dose
30 minutes	30 minutes after 1st fosfomycin oral dose
60 minutes	60 minutes after 1st fosfomycin oral dose
SAMPLE 4: ONLY ONE OF:	
2 hours	2 hours after 1st fosfomycin intravenous dose
4 hours	4 hours after 1st fosfomycin intravenous dose
8 hours	8 hours after 1st fosfomycin intravenous dose
Dose: 2nd Fosfomycin PO	12 hours after 1st fosfomycin oral dose; *continuing 12 hourly for up to 7 days*
SAMPLE 5: (collected alongside biochemistry)	7 days after 1st fosfomycin intravenous dose

****Note: Subjects will be allocated to only one of each sampling timeframe***

Samples will be collected into heparinised tubes, centrifuged and the separated plasma stored at minus 80°C. Biochemistry will be measured in real time, and results returned immediately to assist clinical care.
If a lumbar puncture is conducted and processed in the laboratory for clinical reasons, residual CSF will be stored at minus 80°C to assess CSF antimicrobial penetration.

Samples will be securely stored on site in the KEMRI-CGMRC in Kilifi. Following export approval by SERU, samples will be packaged and shipped according to IATA regulations by a reputable courier, such as World Courier to Analytical Services International Ltd. At St. George's Hospital, London and University College London for analysis. Pharmacokinetic assay results will be returned to Kenya. Samples may be stored for up to 5 years. Additional analyses beyond those described in this protocol: assaying antimicrobial drug levels and biochemistry/liver function will first require further approval by SERU. Remaining samples will be destroyed by incineration according to GCP after 5 years.

	SCREENING	BASELINE	←-----------TREATMENT PHASE---------→				FOLLOW-UP
PROCEDURES			Daily until discharge	48 hours	Day 7	Discharge	Day 28
Informed Consent	X						
Demographics	X						
Medical History	X		X			X	X
Physical Examination	X		X			X	X
Vital Signs (incl. wt)	X		X			X	X
PK Sampling				X#	X^		
Biochemistry	X			X#	X^		
Eligibility Assessment	X						
Allocation		X					
Dispensing Trial Drugs		X		X	X		
Nappy Swab		X				X	
Adverse Event Assessment	X	X	X	X	X	X	X

**Or when tolerating oral medication*

#Acceptable time point: within 12 hours (either side) of 48 hours since commencing IV fosfomycin

^Acceptable time point: within 24 hours (either side) of day 7 since commencing IV fosfomycin

h) Provisions for data verification, and validation

Clinical data will be collected prospectively, supervised by a consultant paediatrician investigator, and overseen by the coordinating team in Kilifi. Data will be monitored for integrity and completeness weekly by the data manager. The study will be monitored by the KEMRI/Wellcome Clinical Trials Facility Monitoring Team. For the PK study, a comprehensive study initiation visit will be done at which all staff will be trained in protocol specific procedure, and check that all trial logistics are in place. A site initiation visit, then routine monitoring will be conducted during PK study. The monitor's role is part of the quality system that will ensure that all participants have duly completed informed consent; entries relating to eligibility, consent and data collected on the CRF are source-verified; and that staff on the study are following standard operating procedures (SOPs) which are in accordance with the protocol.

Clinical Trial Governance:

An independent DSMB will have an advisory role to safeguard the interests of trial's participants, investigators and sponsor; to assess the safety and efficacy of the trial's intervention, and to monitor the trial's overall conduct, and protect its validity and credibility. Its recommendations will be addressed by the TSC. The DSMB operations will be facilitated by the PI and TSC. The DSMB will usually be convened annually, by teleconference, at the chair's discretion. The chair may also call for *ad hoc* or emergency meetings. The DSMB will report to the TSC, usually within 2 weeks of a meeting, copied to the trial statistician. Unless the DSMB is recommending that the trial protocol be changed, the letter to the TSC should not usually reveal any confidential information. If the DSMB has serious concerns about a TSC decision, a meeting of these groups should be held, chaired by an external expert not directly involved with the trial.

Safety reporting

i) Assessment of Safety:

Neonates will be reviewed every day by study clinicians, working together with the hospital team. All adverse events will be documented and reported, with any grade 4 AE or Serious AE (SAE) that are not 'Anticipated SAEs' (see below) will be reported immediately (no later than within 24h of knowledge by the investigator) to

the Sponsor, within 48 hours of knowledge by the Sponsor (DNDi) to an independent DSMB, and within 72 hours of knowledge by the Sponsor to the Ethics Committee and PPB.

Safety will be assessed through routine monitoring of adverse events. In addition, evaluation of haematology and blood chemistry parameters, regular measurement of vital signs and physical examinations will be conducted as per the protocol and clinical indication. The frequency, severity, seriousness and causality assessments of AEs will be described, as well as frequency of SAEs or AEs that lead to treatment discontinuation. AEs will be collated for both patient groups in the CRF. The AE reporting period begins upon issuance of a clinical trial participant number and ends at the day 28 follow-up visit.

ii) Protocol Violations:

Any protocol violations will be reported to the to the Sponsor (DNDi), to an independent DSMB, and to the Ethics Committee and PPB.

iii) Laboratory examinations

Haematology parameters (CBC, WBC with differential and platelets) will be analysed at screening, on days 2 and day 7 as is normally clinically indicated (plus any additional investigations as clinically indicated). Biochemistry parameters will be analysed at screening, on day 2 and day 7 (plus additional frequency if clinically indicated). Samples will be analysed at the adjacent KWTRP Clinical Trials Laboratory using standardized equipment. Approximately 0.5ml of blood will be collected for each study investigation. Abnormal Lab parameters will be assessed for clinical significance.

iv) Adverse event definitions and reporting

The definition of adverse drug reactions, events or suspected unexpected serious adverse reactions is outlined below:

TERM	DEFINITION
Adverse Event (AE)	Any untoward medical occurrence in a patient or clinical trial participant administered a medicinal product and which does not necessarily have a causal relationship with this treatment[A]
Adverse Drug Reaction (ADR)	All untoward and unintended responses to an investigational medicinal product related to any dose administered[B]
Serious Adverse Event (SAE) or<br>Serious Adverse Reaction (SAR)	Any untoward medical occurrence or effect that at any dose which:<br>• Results in death<br>• Is life-threatening<br>• Requires hospitalization or prolongation of existing hospitalization<br>• Results in persistent or significant disability or incapacity<br>• Is a congenital anomaly or birth defect

TERM	DEFINITION
	• Is considered medically important/clinically significant[C]
Non-Serious Adverse Event / Non-Serious Adverse Drug Reaction	An adverse event or adverse drug reaction that does not meet the criteria listed above for a serious adverse event/serious adverse drug reaction
Expected Adverse Reaction (EAR)	An adverse reaction, the nature or severity of which is consistent with the SPC for Fosfocina, 500 mg capsules and suspension (250mg/5ml) Laboratorios ERN; or Fomicyt, 40mg/ml powder for solution for infusion, Nordic Pharma
Unexpected Adverse Reaction (UAR)	An adverse reaction, the nature or severity of which is not consistent with the SPC for Fosfocina, 500 mg capsules and suspension (250mg/5ml) Laboratorios ERN; or Fomicyt, 40mg/ml powder for solution for infusion, Nordic Pharma
Suspected Unexpected Serious Adverse Reaction (SUSAR)	An adverse reaction that is unexpected (not consistent with the SPC for Fosfocina, 500 mg capsules and suspension [250mg/5ml] Laboratorios ERN; or Fomicyt, 40mg/ml powder for solution for infusion, Nordic Pharma) and meets the definition of a serious adverse event/reaction

An AE can therefore be any unfavourable and unintended sign (including an abnormal laboratory finding), symptom, or disease temporally associated with the use of an investigational medicinal product (IMP), whether considered related to the IMP.

What is not an AE?

- Medical conditions present at the initial study visit, that do not worsen in severity or frequency during the study, are not considered as AE.
- Lack of efficacy of the IMP is not considered as AE.

Laboratory/procedures abnormalities considered as an AE:

Laboratory/procedures abnormalities (or worsening in severity or frequency of pre-existing abnormalities) should be assessed as “clinically significant” if they meet AT LEAST ONE of the following conditions:

- The abnormality suggests a disease and/or organ toxicity AND this abnormality was not present at the screening visit or is assessed as having evolved since the screening visit
- The abnormality results in discontinuation of the study drug
- The abnormality requires medical intervention or concomitant therapy

When reporting an abnormal laboratory/procedure result, a clinical diagnosis should be recorded rather than the abnormal value itself, if available (for example, “anaemia” rather than “low haemoglobin”). For every laboratory assessment, the investigator will evaluate if the lab test is normal or abnormal. If abnormal, the investigator will

assess if this finding is clinically significant or not. If a lab parameter is abnormal and clinically significant, it should be reported as an adverse event, after comparison with the previous value (AE).

v) Eliciting Adverse Event information

The investigator will report all directly observed AEs and all AE spontaneously reported by the parents/guardians using concise medical terminology. In addition, at follow-up visit the parents/guardians will be asked a a generic question such as *"Since you were discharged from hospital, has your child had any health problems?"* Information on AEs will be evaluated by a physician.

vi) Adverse Event reporting period

The adverse events reporting period begins upon subject enrolment in the trial (after signature of informed consent) and ends 28 days (4 weeks) after the first dose was administered.

vii) Anticipated Serious Adverse Events:

Adverse events are relatively common in this patient group due to low birth weight, possible birth asphyxia and concomitant disease processes.[46] Anticipated serious adverse events defined in the table below will be recorded and reported in the CRF, but will be exempt from expedited reporting to the Sponsor and regulatory bodies as they are anticipated in this high-risk population. If an Investigator believes that one of these events is causally related to fosfomycin, this would be classified as a SUSAR and requires expedited reporting.

**ANTICIPATED SERIOUS ADVERSE EVENTS**
**Necrotising enterocolitis (diagnostic radiological/surgical changes)**
**Intracranial abnormality on cranial ultrasound scan**<br>**(parenchymal haemorrhage or focal white matter injury)**
**Patent ductus arteriosus**
**Pulmonary haemorrhage**
**Severe anaemia requiring transfusion (due to ABO incompatibility, prematurity or haemorrhage)**
**Jaundice requiring phototherapy or exchange transfusion**
**Congenital birth defect diagnosed during admission**
**Fracture secondary to birth trauma**
**Apnoea**
**Infection (positive blood culture with clinical signs)***
**Persistent derangement of liver function tests (beyond 36 weeks CGA)**
**Abnormal muscle tone, posturing or convulsions (secondary to suspected birth asphyxia)**
**An episode of Hypoglycaemia (defined as per the World Health Organization, ≤2.6mmol/L)**[47]

** Both infections other than condition for inclusion in trial and, in case of infections linked to the sepsis episode that is the reason of the participant's inclusion in the trial, worsening (e.g. septic shock) and relapse.*

viii) Adverse Event Recording:

All AEs will be recorded on the AE CRF and reported as a listing at the end of the study. In the CRF, a given AE will be recorded one time per subject, and the severity will be assessed and recorded as the maximum level reached. If several distinct episodes of the same condition occur, their number will be recorded in the CRF. In addition, the causal relationship between the onset of each AE and the IMP (for those not already identified as anticipated SAEs) will be assessed.

Information on adverse events will be evaluated by a physician. Each adverse event will be classified by the investigator as serious or non-serious. This classification will determine the reporting procedure for the event.

All unexpected serious adverse events (SAE) will be reported immediately to DNDi and SERU (the Sponsor) within 24 hours of awareness by the investigator, and within 48 hours of awareness of SUSAR by the investigator to OxTREC, PPB and the DSMB (whether or not the event is considered study drug-related), using the SAE report form. This includes a description of the event, onset date and type, duration, severity, relationship to study drug, outcome, measures taken and all other relevant clinical and laboratory data. The initial report will be followed by submission of additional information (follow-up SAE form) as it becomes available.

Any follow-up reports will be submitted within 5 working days. In addition to documenting the SAE on the SAE report form, the SAE will also be documented on the CRF and all medications used to treat the SAE will be documented on the concomitant treatments CRF.

ix) Grading of Adverse Event severity

Toxicities and adverse events will be graded for severity to describe the maximum severity of the adverse event according to the DAIDS grading scales (version 2.1, March 2017; Available at: https://rsc.tech-res.com/docs/default-source/safety/daids-ae-grading-table-mar2017.pdf?sfvrsn=6).

In case of AEs that are not described in the DAIDS AE grading system, the investigator will use the terminology “mild”, “moderate”, “severe” or “life-threatening” to describe the maximum severity of the adverse event. These severity grades are defined as follows:

MILD	Does not interfere with subject's usual functions
MODERATE	Interferes to some extent with subject's usual functions
SEVERE	Interferes significantly with subject's usual functions
LIFE-THREATENING:	The subject is at risk of death at the time of the AE it does not refer to an AE that hypothetically might have caused death if more severe.

It is to be noted there exists a distinction between severity and seriousness of adverse events. *A severe adverse event is not necessarily a serious event.*

x) Adverse Event causality assessment

For both serious and non-serious AEs, the investigator is required to assess the causal relationship between the AE and the study drug, i.e. to determine whether there exists a reasonable possibility that the study drug caused or contributed to the adverse event, or evidence to suggest a causal relationship.

To help investigators with the decision binary tree in the evaluation of causality, as per the the CIOMS VI group recommendation, the investigators will consider the following before reaching a decision:

- Medical history
- Lack of efficacy/worsening of existing condition
- Study medications
- Other medications (concomitant or previous)
- Withdrawal of study medication, especially following trial discontinuation / end of study medication
- Erroneous treatment with study medication (or concomitant)
- Protocol related procedure

The terms for reporting are:

Definite	The adverse event and administration of IMP are related in time, and a direct association can be demonstrated.
Probable	The adverse event and administration of IMP are reasonably related in time, and the adverse event is more likely explained by study agent than other causes
Possibly related	The adverse event and administration of IMP are reasonably related in time, and the adverse event can be explained equally well by causes other than IMP
Unlikely	A potential relationship between IMP and the adverse event could exist (i.e. the possibility cannot be excluded), but the adverse event is most likely explained by causes other than the IMP
Not related	The adverse event is clearly explained by another cause not related to the IMP

xi) Adverse event follow up

All participants with AEs will receive treatment for those events and be followed up until they are resolved, or the investigator assesses them as chronic or stable, or the subject participation in the study ends (i.e., until a final report is completed for that subject) and care is ongoing.

In addition, all SAEs and those non-serious AEs assessed by the investigator as related (related/probably related/possibly related) to the investigational medication will continue to be followed even after the subject participation in the study is over. Such events will be followed until they resolve or until the investigator assesses them as chronic or stable. Resolution of such events will be documented on the CRF.

Withdrawal criteria:

A subject should be withdrawn from the study treatment if, in the opinion of the investigator, it is medically necessary, or if it is the wish of the parent/guardian. If a patient is withdrawn from the study before the full course of the treatment is completed, the physician will make all necessary arrangements to ensure that the subject receives the appropriate treatment for the relevant medical condition (ie with medication/s currently recommended by national guidelines). Should a patient require second-line or alternative antimicrobials, they will not be removed from the study as this will not impact the fosfomycin PK data.

If a subject does not return for the follow-up assessments, every effort will be made to contact their parents/guardian. In any circumstance, every effort should be made to document subject outcome, if possible. If the subject withdraws consent, no further evaluations should be performed and no attempts should be made to collect additional data, except for safety data, which should be collected if possible and in accordance with consent.

If a subject is withdrawn from the study, the reason will be noted on the CRF. If a subject is withdrawn from the study because of a treatment limiting adverse event, thorough efforts will be made to clearly document the outcome of AE.

Data Safety Monitoring Board:

A Data Safety Monitoring Board (DSMB), composed of 3 members independent of the investigator and sponsor, will be set up prior to study initiation. The DSMB monitors the study in order to ensure that harm is minimized, and benefits maximized for the study subjects. They will review the study data at pre-determined intervals and issue recommendations about the study. The data and intervals will be agreed prior to, or soon after, the study initiation and documented in the DSMB Charter.

Quality Assurance and Quality Control Procedures

A) Investigator's file:

The investigator must maintain adequate and accurate records to enable the conduct of the study to be fully documented and the study data to be subsequently verified. These documents include:

- Investigator's Site File
- Subject clinical source documents
- Screening / enrolment logs.

The Investigator's Site File will contain the protocol/protocol amendments, CRF and query forms, REC and regulatory approval with correspondence, sample informed consent, drug accountability records, staff curriculum vitae, authorization forms and other appropriate documents and correspondence.

B) Case report forms (CRFs):

For all participants, a study-specific standardized daily record (which will constitute part of the CRF) will be used from admission (enrolment), with data entered directly into the CRF, and subsequently into the trial database. At discharge, the date, vitals status and weight will be recorded. Clinical data will be collected prospectively, supervised by a consultant paediatrician investigator to ensure data completeness and accuracy. Data that are derived should be consistent with the source documents or the discrepancies will be explained. All CRF data will be anonymized (identified by study patient number only). The study will be reviewed by the KEMRI/Wellcome Clinical Trials Facility Monitoring Team. Study monitors will raise queries on data discrepancies, and these will be corrected by the study investigator against verified source information.

The investigators will ensure the accuracy, completeness, legibility, and timelines of all data reported to the sponsor in the CRFs and any other additional information that is required. The investigator is responsible for keeping all consent forms, screening forms, CRF and the completed subject identification code list in a secure location.

C) Source documents:

The verification of the CRF data will be by direct inspection of source documents. Source documents include subject hospital/clinic records, physician's and nurse's notes, appointment book, original laboratory reports, letters, and subject screening and enrolment logs. The Investigator / designee will record the date of each subject's visit together with a summary of their status and progress in the study. The investigator will maintain source documents for possible review and/or audit by DNDi, Ethics Committees and/or Regulatory Authorities.

D) Record Retention:

The sponsor will keep all study documents on file for at least 25 years after completion or discontinuation of the study, at a secure facility contracted by the DNDi Nairobi offices, within Kenya. After that period of time the documents may be destroyed with prior permission from DNDi, subject to local regulations. Should the investigator wish to assign the study records to another party or move them to another location, DNDi will be notified in advance.

E) Monitoring, audits and inspections:

The investigators will permit representatives of DNDi, designated clinical monitors and representatives of Ethics Committees or Regulatory Authorities to review all CRFs, medical records, laboratory work sheets and to assess the status of drug storage, dispensing and retrieval at any time during the study. The Investigator's File and

corresponding source documents for each subject will be made available provided that subject confidentiality is maintained in accordance with local regulations. The monitoring, audits or inspections are for the purpose of verifying the adherence to the protocol and to ensure the study is conducted according to GCP. It is important that the investigators and other trial site staff are available at these visits.

F) Protocol Amendments:

The principal investigator will ensure that the study protocol is strictly adhered to throughout, and that all data are collected and recorded correctly on the CRF. All protocol modifications must be documented in writing. Any protocol amendment must be approved and signed by the sponsor and the Principal investigator and is to be submitted to the appropriate IEC for information and approval in accordance with local requirements, and to regulatory agencies if required. Approval by IEC (and Regulatory Authority, if applicable) must be awaited before any changes can be implemented, except for changes necessary to eliminate an immediate hazard to trial subjects, or when the change involves only logistical or administrative aspects of the trial [e.g. change in clinical monitor[s], change of telephone number[s].

The protocol amendment can be initiated by either sponsor or by any Principal investigator. The investigator will provide in writing the reasons for the proposed amendment and will discuss with the medical coordinator and sponsor.

Data Sharing

The results of this work will be accessible with no financial barriers, by contributing to open source initiatives such as public databases and open access journals in a timely fashion. Permission to utilize anonymized data will be by application to the Data Governance Committee at CGMR-C who will ensure that appropriate ethical approval is in place for any new analysis. Explanation of this eventuality will be included in the participant Information and Consent Form (ICF).

Intellectual property

Any intellectual property rights that arise from the work will be safeguarded according to DNDi's IP policy and current KEMRI guidelines and the Industrial Property Act of 2001, sections 32, 58 and 80. The scientific and intellectual contributions of all persons involved in the research will be appropriately acknowledged in all publications and presentations arising from the work.

Time Frame/Duration of the Project

May 2017 – December 2017: MIC analysis (once approval is granted)

October 2017 – February 2018: PK study (once approval is granted)

February 2018 – June 2018: Analysis and write-up / publication

Activity	Q1 2017	Q2 2017	Q3 2017	Q4 2017	Q1 2018	Q2 2018
Scientific & ethical approval	X	X				
Database set up		X				
Training		X	X			
PK recruitment & sampling			X	X	X	
PK analysis				X	X	

PK dissemination				X	X	
MIC analysis			X	X	X	
Write-up				X	X	X
Publication						X

Table 7: GANTT Chart of Work Package

Ethical Considerations

The experimental protocol for this study has been designed in accordance with the general ethical principles outlined in the Declaration of Helsinki and ICH guidelines for Good Clinical Practice (International Committee for Harmonization). DNDi assures that it will comply with all applicable state, local and foreign laws for protecting the rights and welfare of human subjects. This protocol and any protocol amendments will be reviewed / approved by the KEMRI Scientific and Ethical Review Unit, Nairobi and the Oxford Tropical Research Ethics Committee, Oxford, UK. The study will be registered on www.clinicaltrials.gov.

Participation in research is voluntary and consent must be given with free will of choice, and without undue inducement. The parent/legal guardian must have sufficient knowledge and understanding of the nature of the proposed research, the anticipated risks and potential benefits, and the requirements of the research to be able to make an informed decision.

b) Human Subjects
First do no harm

(i) Risks

The study will be performed in a patient group who may potentially benefit from the treatment. Fosfomycin is licensed for use in neonates throughout Europe and is used to treat resistant UTIs among adults in Europe and the USA. Adverse events are reported to be rare, and monitoring of renal function, sodium and potassium levels should pre-empt major adverse clinical outcomes from occurring.

The risks of blood drawing include pain and thrombophlebitis. These will be minimized by careful aseptic technique according to a standard SOP. No more than 1ml/kg of blood will be drawn for research at any one time, and no more than a total of 2.5ml will be drawn for research during the entire study. DNDi has a specialist insurance policy in place which would operate in the event of any participant suffering harm as a result of their involvement in the performance of the study.

(ii) Benefits to the Patients and Community as a whole

Additional clinical staff will be recruited and will undertake study duties and assist in care, adding to the staff available for clinical care on the wards. For babies who do not improve on the currently recommended standard-of-care, we will provide second-line antibiotics free of charge if they are required. This study contributes to knowledge informing the appropriate use of antimicrobials, thus benefiting the whole community both at a local and international level.

(iii) Confidentiality

On enrolment, participants will be issued with a unique identifying number and names recorded on CRFs and in the database as initials only. All clinical data will be held confidentially, and the investigator will ensure that

subjects' anonymity will be maintained, and their identities protected from unauthorized parties. No documents containing patient identity will be submitted to the sponsor. The investigator will maintain documents for submission to sponsor authorized representatives, and the subject's signed written consent forms, in strict confidence.

(iv) Community Engagement Strategy

Community engagement will commence prior to the study with a stakeholders meeting involving Ministry of Health County staff and relevant national Government authorities and policy makers. This will be facilitated by the study sponsor, with attendance by investigators. Ongoing community engagement will be through regular meetings with the community involving KEMRI-Community Representatives and County Government Health and hospital management teams. At these meetings, information and feedback will be given and received.

(v) Stakeholder information giving

We will engage key individuals whom patients may receive information from, including nurses and all ward staff. We will expand ongoing communication activities about research to include this study in order to support parents and guardians receiving information on the study, before being asked for consent.

(vi) Individual informed consent process

Written consent will be required for all data and samples taken for research purposes. Consenting will be done in a separate area to ensure privacy and the opportunity to ask questions and discuss concerns in the paediatric ward, high dependency unit or in casualty once the decision to admit has been made. It is the responsibility of the investigator / designee to obtain written informed consent for each individual participating in this study, after adequate presentation of the aims, methods, anticipated benefits, and potential hazards of the study. The written informed consent document will be translated into the local language (Swahili and Giriama). If needed, the person will be given time to discuss the information received with other members of the family before deciding to consent, providing this does not extend beyond the time identified for inclusion in the study as per the inclusion criteria. The subject or parent/guardian will be asked to provide written and signed consent. Parents or legal guardians will be able to consent separately for participation in the study, storage of data and samples for future research, and export of samples for investigations that cannot currently be conducted in Kenya. Where the attending clinician judges that the child is so severely ill that adequate communication with the parent or legal guardian is not possible, the child will be excluded from participation. If the parent /guardian is illiterate, a literate witness must sign (this person should have no connection to the research team and the sponsor, and, if possible, should be selected by the participant). The witness shall attest that they have provided information accurately to the parent/guardian and this was understood; a thumbprint of the parent/guardian must be provided to attest to this. If new safety information results in significant changes in the risk/benefit assessment, the consent form should be reviewed and updated if necessary. All subjects (including those already being treated) should be informed of the new information, given a copy of the revised form and give their consent to continue in the study.

(vii) Training / support for those involved in community engagement and administering consent

Clinical officers, nurses and field workers will be trained in providing information and administering the consent procedure, following a standard operating procedure in the local language of participants, using didactic learning and role plays. In addition, all investigators will complete relevant courses in Good Clinical Practice ethical training specifically addressing research involving human subjects.

(viii) Feedback of information

This study will be undertaken with the medical and nursing unit staff and the hospital consultants, who have been involved in its design and will be essential in its implementation. Information arising from the study will be fed back through hospital-wide meetings. Study results will be disseminated to study staff, hospital staff and local communities through meetings targeting respective groups. Results will also be published in peer-reviewed journals and presented at local and international meetings or conferences.

(ix) Animal Subjects
N/A

<u>*Expected Application of the Results*</u>
The results will contribute to both local and international knowledge regarding the appropriate use of antimicrobials in neonates with presumed sepsis, and help in the design of a subsequent multi-centre, large-scale randomized clinical trial to determine the risks and benefits (in terms of mortality and antimicrobial resistance) of an updated antibiotic schedule, particularly where multidrug resistant bacteria are prevalent.

References

1 Lawn J, Blencowe H, Oza S, You D, Lee A. Every Newborn: Progress, Priorities, and Potential beyond Survival. *Lancet* 2014; **384**: 189–205.

2 Liu L, Oza S, Hogan D, *et al.* Global, regional, and national causes of child mortality in 2000-2013; with projections to inform post-2015 priorities: an updated systematic analysis. *Lancet* 2014; **385**: 430–40.

3 Shah D, Doyle L, Anderson P, *et al.* Adverse neurodevelopment in preterm infants with postnatal sepsis or necrotizing enterocolitis is mediated by white matter abnormalities on magnetic resonance imaging at term. *J Pediatr* 2008; **153**: 170–5.

4 The World Health Organization. Pocket Book of Hospital Care for Children: Guidelines for the Management of Common Illnesses with Limited Resources. 2013. http://apps.who.int/iris/bitstream/10665/43206/1/9241546700.pdf.

5 Downie L, Armiento R, Subhi R, Kelly J, Clifford V, Duke T. Community-acquired neonatal and infant sepsis in developing countries: efficacy of WHO's currently recommended antibiotics- systematic review and meta-analysis. *Arch Dis Childhood* 2013; **98**: 146–54.

6 Le Doare J. Heath, P. and Sharland, M. KB. Systematic review of antibiotic resistance rates among gram-negative bacteria in children with sepsis in resource-limited countries. *J Paediatr Infect Dis Soc* 2014; **4**: 11–20.

7 Folgori L, Livadiotti S, Carletti M, *et al.* Epidemiology and clinical outcomes of multidrug-resistant, gram-negative bloodstream infections in a European tertiary paediatric hospital during a 12-month period. *Pediatr Infect Dis J* 2014; **33**: 929–32.

8 Delhi Neonatal Infection Study (DeNIS). Characterisation and antimicrobial resistance of sepsis pathogens in neonates born in tertiary care centres in Delhi, India: a cohort study. *Lancet Glob Heal* 2016; **4**: e752–60.

9 Laxminarayan R, Matsoso P, Pant S, *et al.* Access to effective antimicrobials: A worldwide challenge. *Lancet* 2016; **387**: 168–75.

10 The World Health Organization. Critically important antimicrobials for human medicine. 3rd Revis. 2011.

11 Hendlin D, Stapley M, Jackson H, Wallick A, Miller A, Wolf F. Phosphonomycin. A new antibiotic produced by strains of Streptomyces. *Science (80-)* 1969; **166**: 122–3.

12 Michalopoulos AS, Livaditis IG, Gougoutas V. The revival of fosfomycin. *Int J Infect Dis* 2011; **15**: e732–9.

13 Falagas M, Vouloumanou E, Samonis G, Vardakas K. Fosfomycin. *Clin Microbiol Rev* 2016; **29**: 321–47.

14 Vardakas KZ, Legakis NJ, Triarides N, Falagas ME. Susceptibility of contemporary isolates to fosfomycin: a systematic review of the literature. *Int J Antimicrob Agents* 2016; **47**: 269–85.

15 Iarikov D, Wassel R, Farley J, Nambiar S. Adverse Events Associated with Fosfomycin Use: Review of the Literature and Analyses of the FDA Adverse Event Reporting System Database. *Infect Dis Ther* 2015; **4**: 433–58.

16 Baquero F, Hortelano JG, Navarro M, *et al.* Antibiotherapy of Serratia marcescens septicemia in children. *Chemotherapy* 1977; **23 Suppl 1**: 416–22.

17 Corti N, Sennhauser FH, Stauffer UG, Nadal D. Fosfomycin for the initial treatment of acute haematogenous osteomyelitis. *Arch Dis Child* 2003; **88**: 512–6.

18 Llorens J, Lobato A, Olay T. The passage of fosfomycin into the cerebrospinal fluid in children's meningitis. *Chemotherapy* 1977; **23 Suppl 1**: 189–95.

19 Careddu P, Borzani M, Scotti L, Varotto F, Garlaschi L, Fontana P. Treatment of lower urinary tract infections in children: single dose fosfomycin trometamol versus pipemidic acid. *Chemioterapia* 1987; **6**: 290–4.

20 Principi N, Corda R, Bassetti D, Varese LA, Peratoner L. Fosfomycin trometamol versus netilmicin in children's lower urinary tract infections. *Chemotherapy* 1990; **36 Suppl 1**: 41–5.

21 Varese LA. Trometamol salt of fosfomycin versus netilmicin: randomized multicenter study in children's lower urinary tract infections. *Eur Urol* 1987; **13 Suppl 1**: 119–21.

22 Nutrition A et al. for the EC on. Enteral Nutrient Supply for Preterm Infants: Commentary From the European Society for Paediatric Gastroenterology, Hepatology, and Nutrition Committee on Nutrition. *Hepatol Nutr* 2010; **50**: 85–91.

23 Group K et al. for the PNGW. Guidelines on Paediatric Parenteral Nutrition of the European Society of Paediatric Gastroenterology, Hepatology and Nutrition (ESPGHAN) and the European Society for Clinical Nutrition and Metabolism (ESPEN), Supported by the European Society of Paediatri. *J Pediatr Gastroenterol Nutr* 2005; **41**: S1–S4.

24 Nash P. Potassium and Sodium Homeostasis in the Neonate. *Neonatal Netw* 2007; **26**: 125–8.

25 Akçam M. Oral fructose solution as an analgesic in the newborn: A randomized, placebo-controlled and masked study. *Pediatr Int* 2004; **46**: 459–62.

26 Bueno M, Yamada J, Harrison D, *et al.* A systematic review and meta-analyses of nonsucrose sweet solutions for pain relief in neonates. *Pain Res Manag* 2013; **18**: 153–61.

27 Taylor CG, Mascaros E, Roman J, *et al.* Enteropathogenic E. coli gastroenterocolitis in neonates treated with fosfomycin. *Chemotherapy* 1977; **23 Suppl 1**: 310–4.

28 Rossignol S, Regnier C. [Fosfomycin in severe infection in neonatology]. *Ann Pediatr (Paris)* 1984; **31**: 437–44.

29 Guillois B, Guillemin MG, Thoma M, Sizun J, Monnery JL, Alix D. [Neonatal pleuropulmonary staphylococcal infection with multiple abscesses of the liver]. *Ann Pediatr (Paris)* 1989; **36**: 681–4.

30 Gouyon JB, Francois C, Semama D, Sandre D, Duez JM, Portier H. [Nosocomial Staphylococcus epidermidis and Staphylococcus aureus septicemias in neonates]. *Ann Pediatr (Paris)* 1990; **37**: 21–5.

31 Algubaisi S, Buhrer C, Thomale U-W, Spors B. Favorable outcome in cerebral abscesses caused by Citrobacter koseri in a newborn infant. *IDCases* 2015; **2**: 22–4.

32 Li G, Standing J, Bielicki J, *et al.* The potential role of fosfomycin in neonatal sepsis caused by multidrug resistant bacteria. *Publ* 2016.

33 Guggenbichler JP, Kienel G, Frisch H. [Fosfomycin, a new antibiotic drug (author's transl)]. *Padiatr Padol* 1978; **13**: 429–36.

34 Molina MA, Olay T, Quero J. Pharmacodynamic data on fosfomycin in underweight infants during the neonatal period. *Chemotherapy* 1977; **23 Suppl 1**: 217–22.

35 Bergan T. Degree of absorption, pharmacokinetics of fosfomycin trometamol and duration of urinary antibacterial activity. *Infection* 1990; **18 Suppl 2**: S65-9.

36 Bergan T, Thorsteinsson SB, Albini E. Pharmacokinetic profile of fosfomycin trometamol. *Chemotherapy* 1993; **39**: 297–301.

37 Guibert M, Magny JF, Poudenx F, Lebrun L, Dehan M. [Comparative pharmacokinetics of fosfomycin in the neonate: 2 modes of administration]. *Pathol Biol (Paris)* 1987; **35**: 750–2.

38 Suzuki S, Murayama Y, Sugiyama E, Sekiyama M, Sato H. [Dose estimation for renal-excretion drugs in neonates and infants based on physiological development of renal function]. *Yakugaku Zasshi* 2009; **129**: 829–42.

39 Traunmuller F, Popovic M, Konz K-H, Vavken P, Leithner A, Joukhadar C. A reappraisal of current dosing strategies for intravenous fosfomycin in children and neonates. *Clin Pharmacokinet* 2011; **50**: 493–503.

40 Patel S, Balfour J, Bryson H. Fosfomycin tromethamine. A review of its antibacterial activity, pharmacokinetic properties and ther- apeutic efficacy as a single-dose oral treatment for acute uncomplicated lower urinary tract infections. *Drugs* 1997; **53**: 637–56.

41 Duez J, Mousson C, Siebor E, *et al.* Fosfomycin and its application in the treatment of multidrug-resistant Enterobacteriaceae infections. *Clin Med Rev Ther* 2011; **3**: 123–42.

42 Borgia M, Longo A, Lodola E. Relative bioavailability of fosfo- mycin and of trometamol after administration of single dose by oral route of fosfomycin trometamol in fasting conditions and after a meal. *Int J Clin Pharmacol Ther Toxicol* 1989; **27**: 411–17.

43 WHO | Antimicrobial resistance: a priority for global health action. *Bull World Heal Organ* 2015; **93**: 437–512.

44 Sarnat H, Sarnat M. Neonatal encephalopathy following fetal distress: A clinical and electroencephalographic study. *Arch Neurol* 1976; **33**: 696–705.

45 Lu C, Liu C, Huang Y, *et al.* Antimicrobial Susceptibilities of Commonly Encountered Bacterial Isolates to Fosfomycin Determined by Agar Dilution and Disk Diffusion Methods. *Antimicrob Agents Chemother* 2011; **55**: 4295–4301.

46 Morgan C, Herwitker S, Badhawi I, *et al.* SCAMP: standardised, concentrated, additional macronutrients, parenteral nutrition in very preterm infants: a phase IV randomised, controlled exploratory study of macronutrient intake, growth and other aspects of neonatal care. *BMC Pediatr* 2011; **11**.

47 The World Health Organization. Hypoglycaemia of the Newborn: Review of the Literature. 1997 DOI:WHO/CHD/97.1 / WHO/MSM/97.1.

Intravenous and Oral Fosfomycin in Hospitalized Neonates with Clinical Sepsis: an open-label safety and pharmacokinetics study (neoFosfo)

Short title	Neo-Fosfo
Name of product(s)	Fosfomycin oral and IV formulations
Drug Class	Antibiotic
Indication	
Protocol Number	Neo-Fos-001
EudraCT	Not applicable
Study Sponsor	DND*i*<br>Tetezi Towers, 3rd Floor<br>George Padmore Rd<br>Kilimani<br>PO BOX 21936-00505<br>Nairobi<br>KENYA<br>+254 20 3995 000<br>Head Office: Chemin Louis Dunant, 15, 1202 GENEVA Switzerland Phone: +41 22 906 9230
Principal Investigator<br><br>Coordinating Institutions	Professor James A. Berkley<br><br>KEMRI/Wellcome Trust Research Programme<br>PO Box 230, Kilifi, 80108, Kenya<br><br>University of Oxford, University Offices, Wellington Square, Oxford, OX1 2JD, UK
Clinical Trial Protocol Version / Date	Version 1.1 dated 23rd August 2017
Protocol Amendment Number / Date	Version 2.0 dated 13th April 2018

The information contained in this document is confidential. It is to be used by investigators, potential investigators, consultants, or applicable independent ethics committees. It is understood that this information will not be disclosed to others without written authorisation from DNDi, except where required by applicable local laws

CLINICAL TRIAL PROTOCOL SIGNATURE PAGE

Statistician

Signature	______________________	______________
		Date of Signature (DD/MMM/YY)
Name	Sarah Walker	
Title	Professor	
Institution	MRC Clinical Trials Unit	
Address	University College London, London, UK	

Principal Investigator

Investigator Signature	______________________	______________
		Date of Signature (DD/MMM/YY)
Name	Prof James A Berkely	
Title	Professor	
Institution	KEMRI/Wellcome Trust Research Programme	
Address	PO Box 230, Kilifi, 80108, Kenya	

Investigators Signature Page

I have read this protocol and agree that it contains all necessary details for carrying out this trial. I will conduct the trial as outlined herein and will complete the trial within the time designated.
I will provide copies of the protocol and all pertinent information to all individuals responsible to me who assist in the conduct of this trial. I will discuss this material with them to ensure they are fully informed regarding the drug and the conduct of the trial.
I will use only the informed consent form approved by the sponsor or its representative and will fulfil all responsibilities for submitting pertinent information to the Institutional Review Board/Independent Ethics Committee (IRB/IEC) responsible for this trial if required by national law.
I agree that the sponsor or its representatives shall have access to any source documents from which case report form information may have been generated.

Table of Contents

Supplementary Methods .... **Error! Bookmark not defined.**
Study Site and Population .... **Error! Bookmark not defined.**
Screening and Enrolment of Study Participants .... **Error! Bookmark not defined.**
Procedures .... **Error! Bookmark not defined.**
Study Treatment .... **Error! Bookmark not defined.**
Clinical Definitions .... **Error! Bookmark not defined.**
Safety Assessment .... **Error! Bookmark not defined.**
PK Modelling and Simulations .... **Error! Bookmark not defined.**

Supplementary Tables .... **Error! Bookmark not defined.**
Table S1: Baseline laboratory test parameters by treatment arm .... **Error! Bookmark not defined.**
Table S2: Serious and non-serious AEs by treatment arm, severity and MedDRA coding classification .... **Error! Bookmark not defined.**
Table S3. Anticipated adverse events by treatment arm .... **Error! Bookmark not defined.**
Table S4: AE outcome at study termination excluding events that either resolved or occurred in neonates who died .... **Error! Bookmark not defined.**
Table S5 Blood and CSF culture results by treatment arm .... **Error! Bookmark not defined.**

Supplementary Figures .... **Error! Bookmark not defined.**
Figure S1: Outline of study procedures .... **Error! Bookmark not defined.**

References for Supplementary Appendix .... **Error! Bookmark not defined.**

Trial Protocol .... **Error! Bookmark not defined.**

Investigators and Institutional Affiliations 43

Lay summary 44

Abstract 46

Abbreviations 47

Introduction / Background 49

Justification for the study 52

State the Null Hypotheses 52

Objectives 53
General Objectives 53
Specific Objectives 53
Secondary Objective 53

Study Design 53

Study site: 53

Definition of Study Population: 53

Rationale for animal use and justification for animal species chosen 54

Sampling 54

i. Sample size determination: 54

i. For Pharmacokinetics: 54

ii. For Plasma Sodium 54

iii. For MIC of stored bacterial isolates and bowel flora: 55

Procedures: 55

Clinical Trial Governance: 60

Safety reporting 61
i) Assessment of Safety: 61
ii) Protocol Violations: 61
iii) Laboratory examinations 61

iv) Adverse event definitions and reporting 61
v) Eliciting Adverse Event information 62
vi) Adverse Event reporting period 62
vii) Anticipated Events in neonatal setting: 62
viii) Adverse Event Recording: 63
ix) Grading of Adverse Event severity 63
x) Adverse Event causality assessment 64
xi) Grading of Adverse Event seriousness 64
xii) Serious Adverse Event Reporting: 65
xiii) Adverse event follow-up 66

Withdrawal criteria: 66

Data Safety Monitoring Board: 66

Quality Assurance and Quality Control Procedures 66

A) Investigator's file: 66

B) Case report forms (CRFs): 66

C) Source documents: 67

D) Record Retention: 67

E) Monitoring, audits and inspections: 67

F) Protocol Amendments: 67

Data Sharing 67

Intellectual property 67

Expected Application of the Results 69

References 70

Title of the Project

Intravenous and oral fosfomycin in hospitalized neonates with clinical sepsis: An open-label safety and pharmacokinetics study.

Investigators and Institutional Affiliations

KEMRI CGMR-C
James Berkley (PI)
Phoebe Williams
Christina Obiero
Alexander Balo Makazi
Joseph Waichungo
Johnstone Thitiri
Mwanamvua Boga
Sheila Murunga

KEMRI CCR
Martin Ongas

Non-KEMRI*:
Mike Sharland: St George's Hospital, University of London, UK
Julia Anna Bielicki: St George's Hospital, University of London, UK
Karin Kipper: St George's Hospital, University of London, UK
Joseph Standing: University College, London, UK
Sarah Walker: Medical Research Council Clinical Trials Unit at University College London, UK

*Curriculum Vitae of non-KEMRI investigators attached, see appendix E.

Lay summary

A study measuring the levels of an additional antibiotic (fosfomycin) to treat severe infections in newborns.

What is the problem?

Among babies presenting with signs of serious infection, or who develop these signs whilst in hospital, bacterial resistance to the antibiotics that are normally used is increasing. This means that babies with infections may be at a higher risk of dying. In Africa, alternative antibiotics are often expensive and may themselves cause the bacteria to become resistant. Therefore, new treatment strategies are needed. Fosfomycin is a potentially inexpensive antibiotic that is licensed for use in children in Europe and may be useful to resource-poor countries. It has a good safety profile in children and is expected to be effective against infections that do not respond to the currently used antibiotics. However, more information is needed to inform how fosfomycin should be used in babies in Kenya.

What questions are we trying to answer?

We want to find out what doses of fosfomycin would be most suitable for sick newborns in Kenya in order to optimize its use in an effective and safe way. We also want to find out how well local bacteria that have been previously found (and stored) from patients at Kilifi County Hospital are able to be killed by fosfomycin.

Where is the study taking place?

The study will take place in Kilifi County Hospital, Kilifi, Kenya.

How many people does it involve?

For the study measuring the levels of antibiotics in newborn babies, we will include approximately 120 babies admitted to the hospital with presumed infection. Approximately 60 of these babies will be given fosfomycin in addition to standard treatment and drug levels measured; the other 60 will receive standard treatment.

How are these people selected?

We will ask parents and guardians of all babies aged 28 days or less who are admitted to Kilifi County Hospital with signs of infection to participate, unless they are being transferred from another hospital, already received other antibiotics by injection, are very sick or premature, or have abnormally high salt (sodium) levels in their blood.

What does the study involve for those who are in it?

After providing information and answering any questions, informed consent will be requested from the child's parent or guardian. A doctor or study clinical officer will examine the baby and take the usual admission investigations, then prescribe the two antibiotics that are currently recommended by the WHO for the treatment of presumed infection in babies (ampicillin and gentamicin).

Half of the babies will be selected randomly to receive intravenous fosfomycin *as well as* the standard antibiotics. The nurse or clinical officer will then take two blood samples to check fosfomycin levels. After a minimum of four doses of intravenous (IV) fosfomycin (over 48 hours), when their condition is improved and they are tolerating feeds by mouth, the baby will then be changed on to oral fosfomycin. A further two blood samples will be collected after the first oral dose of fosfomycin, including one to check the kidney and liver function and level of salts in the blood (which is currently a routine investigation). Each of these blood samples is 0.5ml, giving a total for this research of 2.5ml (half a teaspoon) for checking the drug levels, and a further 1ml (a quarter of a teaspoon) for checking the level of salts in their blood.

For all babies, a blood test will be taken (which is normally part of routine care) at around 48 hours to check the blood count, kidney and liver function and level of salts in the blood. If a baby has a lumbar puncture as part of their normal treatment (if their doctor is concerned about an infection in their brain), the fosfomycin level in the fluid surrounding their brain will also be checked.

The babies will be closely followed by the study team, working together with the hospital staff to provide the best care available in the hospital. On day 7, any babies who remain as inpatients will have a blood test to check blood count, kidney and liver function and level of salts in the blood (and 0.5ml drawn for fosfomycin levels in the group receiving this antibiotic). Breastfeeding and health counselling will be given according to national guidelines. All babies will be followed up in our outpatients' clinic 28 days after their presentation to hospital

and parents/guardians may also phone the study team directly on a study-specific mobile phone or bring the baby to the ward prior to that review, in case of health concerns.

What are the benefits and risks/costs of the study for those who are involved?

Additional staff (clinical officers and nurses) will be recruited to undertake study duties and assist in general care on the ward, adding to the staff available. Training will be enhanced for all paediatric ward staff on the treatment of babies presenting with infections, and on the prevention of infections within the hospitals. We will also make available additional antibiotics as needed, should a baby continue to have signs of infection despite treatment or remain unwell. Drawing a blood sample carries the potential risks of bruising to the vein or infection, and careful training on procedures will help to prevent these. There may be a small risk of the baby having high levels of salt (sodium) in their blood due to the salt content of the fosfomycin injection. Improved monitoring of kidney function and blood salt levels will offset these risks.

How will the study benefit society?

This study is leading up to a large clinical trial assessing how effective fosfomycin is to treat babies with infections, and if it is effective, will support efforts to make fosfomycin available at low cost for Kenya and other countries. This will help babies to be more effectively treated when bacteria are resistant to the currently used antibiotics.

When does the study start and finish?

The study aims to start as soon as scientific and ethical approval is granted and is expected to continue for 18 months (including analysis and write-up).

Abstract

Antimicrobial resistance (AMR) has become a major issue in global health. Despite progress in the reduction of under 5 mortality rates in recent decades, the proportion of neonatal deaths occurring within this age group has increased, with almost one quarter of all neonatal deaths occurring due to serious bacterial infection. Common bacteria causing neonatal sepsis are now exhibiting widespread resistance to several classes of antibiotics. There is an urgent need to discover new, effective treatments and re-evaluate existing therapeutic agents to treat infections potentially caused by multi-drug resistant (MDR) pathogens. Gram-negative bacteria (GNB) predominate as the cause of neonatal sepsis and are increasingly associated with high rates of resistance to the currently recommended WHO empirical therapy regimen of ampicillin/penicillin and gentamicin. There is therefore a need to develop an updated empiric regimen with improved efficacy in the context of increasing MDR sepsis in neonates. New antimicrobials under development will be expensive once licensed, and there are currently virtually no planned trials to assess their efficacy in neonates in low- and middle-income countries (LMICs).

One potential strategy is utilizing an existing off-patent (and therefore affordable) antibiotic available in intravenous and oral formulations – fosfomycin. Fosfomycin has a wide spectrum of activity against Gram-positive and Gram-negative bacteria causing neonatal sepsis. It is mainly used for resistant urinary tract infections in adults but has licensed neonatal and paediatric doses in Europe (though dosing regimens vary between countries). Both oral and IV formulations are available. A large clinical trial to assess the efficacy of a fosfomycin plus an aminoglycoside combination (compared to the current WHO recommended ampicillin and gentamicin) is anticipated, including sites in Kenya. The ultimate aim is for fosfomycin to be included in the WHO Essential Medicines List for children (EMLc) and be available for use in developing countries, where rates of resistance to ampicillin and gentamicin have been estimated at over 40%. The first steps before this trial are to clarify the pharmacokinetics (PK) and safety profile of fosfomycin in neonates, as well as generating further information regarding local patterns of bacterial susceptibility to fosfomycin. The aim of this study is to fulfil both these steps. Fosfomycin (IV and oral) PK will be investigated among 60 babies admitted to hospital and being treated for presumed sepsis; administered alongside the standard antibiotics. Another 60 babies receiving standard treatment only (without PK sampling) will be monitored in the same way to compare adverse events. In the laboratory at CGMR-C, previously archived bacterial isolates will be tested for their sensitivity to fosfomycin.

Abbreviations

AE	Adverse Event
AGISAR	WHO Advisory Group on Integrated Surveillance of Antimicrobial Resistance
AMR	Antimicrobial Resistance
BSAC	British Society of Antimicrobial Chemotherapy
BW	Birth weight
C_{max}	Peak serum concentration of a therapeutic drug
CBC	Complete blood count
CGMR-C	Centre for geographic medicine research, Coast (Kenya)
CI	Confidence Interval
CLSI	Clinical and Laboratory Standards Institute
CMP	Calcium, Magnesium and Phosphate
CNS	Central Nervous System
CR	Carbapenem resistance/resistant
CRF	Case report form
CRO	Carbapenem resistant organisms
CSF	Cerebrospinal Fluid
DSMB	Drug Safety Monitoring Board
ECCMID	European Congress of Clinical Microbiology and Infectious Diseases
EMLc	Essential Medicines List for children
ESBL	Extended Spectrum Beta-Lactamase
ESPGHAN	European Society for Paediatric Gastroenterology, Hepatology and Nutrition
EUCAST	European Committee on Antimicrobial Susceptibility Testing
FBC	Full blood count
GNB	Gram-negative bacteria
HIE	Hypoxic Ischaemic Encephalopathy
ICF	Informed consent form
IMP	Investigational Medicinal Product
IV	Intravenous
IP	Intellectual Property
LBW	Low birth weight
LMICs	Low- and middle-income countries
LC-MS	Liquid chromatography mass spectrometry
LSM	Local Safety Monitor
MDR	Multi-drug resistant

MIC	Minimum inhibitory concentration
MRSA	Multi-resistant Staphylococcus Aureus
OxTREC	Oxford University Tropical Research Ethics Committee
PK	Pharmacokinetic
PO	Per os (oral)
PPB	Republic of Kenya Ministry of Health Poisons and Pharmacy Board
SAE	Serious Adverse Event
SBI	Serious bacterial infection
SERU	Scientific and Ethics Review Committee (Kenya)
SIADH	Syndrome of inappropriate antidiuretic hormone secretion
SOC	Standard-of-care
SOP	Standard operating procedure
SPC	Summary of Product Characteristics
SUSAR	Suspected Unexpected Serious Adverse Reaction
UTI	Urinary tract infection
VRE	Vancomycin-resistant enterococci
WHO	World Health Organization

Introduction / Background

The purpose of this study is to support the design of an international multi-centre randomized trial of fosfomycin to treat neonates with presumed sepsis, by providing an improved understanding of fosfomycin pharmacokinetics, safety and antimicrobial susceptibility of local invasive bacterial species.

Maternal and child deaths have halved over the past two decades;[1] however neonatal mortality has remained unacceptably high, with an estimated 2.9 million deaths in newborns every year.[2] The proportion of deaths (in children under 5 years) occurring in the neonatal period has increased from 38% to 44% between 2000 and 2013,[2] and 23% of neonatal deaths are due to presumed serious bacterial infections (SBI).[1] Aside from this mortality burden, sepsis in the neonatal period is also associated with significant morbidity secondary to an increased risk of adverse neurodevelopmental outcomes.[3]

The WHO and Kenyan guidelines currently recommend ampicillin (or penicillin) plus gentamicin for the treatment of sepsis in neonates and infants <2 months of old, with third-generation cephalosporins listed as second-line therapy.[4] However, two recent systematic reviews have documented increasing rates of AMR to this regimen.[5,6] Downie et al. (2013) examined 19 studies from 13 LMICs across Asia and Africa, revealing non-susceptibility to penicillin/gentamicin and third-generation cephalosporins of 44% and 43% respectively.[5] Le Doare et al. (2015) identified 15 studies investigating non-susceptibility among Gram-negative pathogens across SE Asia, Africa and the Middle East which revealed Enterobacteriaceae exhibit high rates of non-susceptibility to ampicillin (80%), gentamicin, (22%) and ceftriaxone (74%).[6]

Challenges in interpreting this literature include the limited data available being mostly from urban tertiary hospital settings (rather than district- or community-level facilities), failure to account for prior treatment, not distinguishing community- from hospital-acquired infections, and inconsistent laboratory facilities. Nevertheless a consensus is emerging that AMR to recommended first-line antibiotics in LMICs is associated with significant morbidity and mortality.[7,8] A recent study of neonatal deaths attributable to MDR sepsis (in 5 countries accounting for half the global neonatal sepsis death rates - India, Pakistan, Nigeria, DR Congo and China) identified 214,000 neonatal deaths occurring each year due to resistant bacterial infections.[9] Notable is the emergence and spread of extended spectrum beta-lactamase (ESBL)-producing Enterobacteriaceae, which render many commonly used (and cheaply available) antimicrobials ineffective. Carbapenems are increasingly being used as second-line therapy in neonatal sepsis, but they are expensive, and their use is associated with increasing AMR due to the dissemination of infections with carbapenem-resistant organisms (CRO). There is therefore a need to clarify an empiric regimen with improved for use in LMICs.

The repurposing of older antimicrobials for current treatment regimens has recently received increasing international attention. The WHO Advisory Group on Integrated Surveillance of Antimicrobial Resistance (AGISAR) included fosfomycin in the current revision of critically important antimicrobials for human medicine.[10] Fosfomycin is a bactericidal peptidoglycan antibiotic that was first produced in the 1970s[11] though its marketing was largely replaced in the 1980s by oral cephalosporins. Its infrequent international use over the past 30 years has resulted in low global resistance rates.

Fosfomycin is orally absorbed, crosses the blood brain barrier and is renally excreted. It exhibits minimal toxicity, low levels of cross-resistance, and provides synergistic effects with other antibiotics (including beta-lactams, aminoglycosides and fluoroquinolones).[12] IV fosfomycin is licensed in Europe and the USA as a second-line treatment in adults and children with osteomyelitis, complicated UTI, nosocomial lower respiratory tract infections, bacterial meningitis, or bacteraemia associated with any of these causes. Oral fosfomycin is used for treating UTI caused by Escherichia coli and Enterococcus faecalis.

Fosfomycin has a broad-spectrum of activity against both Gram-negative and Gram-positive organisms, including MRSA and ESBL infections.[13] A recent systematic review evaluated the susceptibility of contemporary bacteria to fosfomycin, revealing 84 studies which documented susceptibility to Staphylococcus aureus (range 33% to 100%); ESBL-producing Escherichia coli (range 81% to 100%); ESBL-producing Klebsiella pneumoniae (range 15% to 100%); and carbapenem-resistant Klebsiella pneumoniae (range 39% to 100%).[14] Thus, fosfomycin currently exhibits high levels of antimicrobial activity against common causes of neonatal sepsis.

The Summary of Product Characteristics (SPC) gives a neonatal intravenous dosing, including for preterm and term infants by age and body weight (Table 1). However, parenteral dosing recommendations for neonates and children patients vary widely between countries in Europe (Table 2), and there are currently no PO dosing recommendations for neonates.

Age/weight	Daily dose
Premature neonates (age [a] < 40 weeks)	100 mg/kg BW in 2 divided doses
Neonates (age [a] 40-44 weeks)	200 mg/kg BW in 3 divided doses
Infants 1-12 months (up to 10 kg BW)	200-300 [b] mg/kg BW in 3 divided doses
Infants and children aged 1-12 years (10-40 kg BW)	200-400 [b] mg/kg BW in 3-4 divided doses

[a] Sum of gestational and postnatal age.

[b] The high-dose regimen may be considered for severe infections and or serious infections (such as meningitis), in particular when known or suspected to be caused by organisms with moderate susceptibility.

Table 1. Parenteral Fosfomycin Neonatal Dosing Recommendations (Nordic Pharma, 2016)

Country	Neonates<br>(pre- & full-term; 0-1 months)	Infants<br>(1-12 months, up to 10kg)	Children<br>(1-12 years; 10-40kg)
Austria	100-200/400* mg/day in 2-3 doses	100-200/400* mg/day in 2-3 doses	4-8g/day, in 2-3 doses
Germany	100mg/day<br>in 2 doses	200-250mg/day in 3 doses	100-200/300*mg/day in 3 doses
United Kingdom	Premature: 100mg/kg in 2 doses<br>Term: 200mg/kg in 3 doses	200-300mg/kg/day in 3 doses	200-400mg/kg in 3-4 doses
Spain	Not specified	Not specified	100-200/400mg/day in 2-3 doses
France	Not specified	Not specified	100-200mg/day; number of daily doses not specified

*Maximum dosage for severe infections

Table 2: Recommended total daily dosages for IV fosfomycin in paediatric patients with normal renal function across various European settings

Safety and Clinical Outcomes of Fosfomycin:

Five published papers have documented the clinical outcomes of (n=84) neonates treated with parenteral fosfomycin therapy for a range of diagnostic situations (Table 3), with no deaths or severe adverse events attributed to this therapy.

A 2015 review of adverse events (AE) reported to the FDA and the international literature in association with fosfomycin administration (in both adult and paediatric patients) concluded that fosfomycin exhibits low toxicity and few concerns regarding its safety profile.[15] This review included data assessing 254 paediatric patients across 6 trials (3 trials of parenteral and 3 of oral fosfomycin; age range: neonates – 15.5 years), 3 of which were retrospective (n=118)[16–18] and 3 prospective randomized trials (n=134)[19–21] investigating oral fosfomycin. In the trials of parenteral fosfomycin, the drug was administered for up to 4 weeks for the treatment of acute hematogenous osteomyelitis, bacteraemia, and lung infection; while oral fosfomycin was administered as a single dose for the treatment of UTI. Overall, no serious safety issues related to the use of fosfomycin in children were identified in this review; with the most frequently reported AEs associated with (IV and PO) administration across all age ranges identified as being rash, peripheral phlebitis and gastrointestinal symptoms. Less common AEs include hypersensitivity and abnormal liver function. These are common AEs which also occur with other antibiotics.

Combined with the 31 babies documented in the literature investigating fosfomycin PK data (discussed below), this results in a total of 367 children in whom fosfomycin has been administered in the published literature with no significant safety concerns having been reported in this cohort.

However, an important potential safety consideration for parenteral fosfomycin is the sodium (Na+) content (14.4mmol/330mg sodium per gram). The European Society for Paediatric Gastroenterology and Hepatology (ESPGHAN) recommends a daily (enteral) sodium intake of 69mg/kg (minimum) to 115mg/kg (maximum) for preterm infants (with enteral values for term infants not published),[22] and a parenteral sodium intake of 2-3mmol/kg/day for term neonates and 3-5mmol/kg/day for premature neonates.[23] Fosfomycin's sodium content equates to a sodium load of 2.8mmol/kg/day (based on dosing of 200mg/kg/day), which is within the published guidelines for neonates. There are negligible amounts of sodium in IV ampicillin and gentamicin, the antibiotics alongside which fosfomycin will be administered; and there is no sodium in the oral fosfomycin formulation.

The ability to reabsorb sodium is inversely proportional to gestational age, and nephrogenesis is complete by 34 weeks gestation.[24] Hence, we aim to restrict our patient population to exclude very preterm infants. Hypernatremia may also occur secondary to hypoxic-ischemic encephalopathy (ie, as a consequence of asphyxiation, due to central diabetes insipidus or via acute renal injury). Therefore, any baby presenting with seizures or with admission sodium ≥150mmol/L or creatinine ≥150micromol/L will be excluded from the study. All poorly feeding babies will receive IV (10% dextrose) fluids (as per Kenyan Paediatric Protocols).

Of note, the oral fosfomycin suspension contains no sodium, using a calcium base at a dose equivalent to 1.4mmol/kg/day, within the published neonatal guidelines for calcium administration (of 1.3-3mmol/kg/day).[23] Monitoring of calcium, magnesium and phosphate will therefore be undertaken. Oral fosfomycin also contains fructose to the equivalent of 1600mg/kg/day. There is little published research regarding high fructose loads in neonates, with most previous trials documenting safety at lower doses (150mg as an analgesic therapy);[25] while a recent meta-analysis evaluating sucrose administration (in 7,049 infants) documented a "very low" incidence of minor adverse events, with no reported major adverse events.[26] Nonetheless, the possible adverse event of osmotic diarrhea will therefore be closely monitored in this study.

Study	N (Total n=82)	Dose and clinical setting	Clinical Setting	Outcomes
Taylor et al. 1977[27]	43 neonates	150-200mg/kg/day	Enterocolitis caused by enteropathic E. coli	Favourable clinical outcome in 88%
Rossignol & Regnier 1984[28]	21 neonates	200mg/kg/day in two divided doses, in combination with gentamicin/tobramycin	Sepsis and UTI	Clinical recovery in 19/21 (90.5%)
Guillois et al. 1989[29]	Case report (n =1)	IV fosfomycin-vancomycin, followed by oral pristinamycin	MSSA septicaemia with a liver abscess	Full recovery
Gouyon et al. 1990[30]	16 neonates	IV fosfomycin-cefotaxime	Staphylococcal septicaemia (epidermidis (n=10) and aureus (n=6)) (including meningitis and osteomyelitis)	Full recovery in n=15 (94%)
Algubaisi et al. 2015[31]	Case report (n=1 term infant)	120mg/kg/day fosfomycin and meropenem	Multiple Citrobacter koseri intracerebral abscesses	Clinical recovery

Table 3: Clinical studies describing the use of fosfomycin in neonatal sepsis. Modified from Li et al (in publication)[32]

Documented Pharmacokinetics of Fosfomycin:

A recent review of the PK profile of fosfomycin in neonates identified four small additional published studies assessing IV fosfomycin (with no oral PK data available) (Table 4). The elimination half-life (t½) of fosfomycin ranged from 2.4-7 hours following an IV bolus of 25-50mg/kg administered to neonates which included LBW and premature infants.[33,34] Fosfomycin is almost completely eliminated by glomerular filtration, with 80-95% of the dose unchanged in the urine within 24 hours.[35] Consequently, neonates have a prolonged fosfomycin t½ compared to older children and adults due to immature glomerular filtration and a greater volume of distribution.[34] Serum protein binding of fosfomycin has been estimated to be below 3%, and the neonatal C_{max} (60-90mg/L) is comparable with adult populations.[35,36]

Study	N (Total n=31)	Dose and study	Outcome
Molina et al. 1977[34]	11 neonates	50mg/kg IV, comparing infants 1-3d old and 3-4 weeks old	Elimination slower at earlier corrected gestational age
Guggenbichler 1978[33]	5 term & 5 pre-term neonates	25mg/kg IV	95-98% recovered in the urine, 1 compartment model
Guibert et al. 1987[37]	10 neonates	200mg/kg BD, comparing 30m or 2hr infusion schedules	No difference between schedules, serum concentrations are above MIC of common pathogens at 12h post dose
Suzuki et al. 2009[38]	Not identified	Dose estimation for renally excreted drugs	Dose estimation validated with GFR, tubular secretion clearance and fraction of unbound drug in plasma

Table 4: Neonatal fosfomycin pharmacokinetic studies; modified from Li et al (2016; in publication)

Bactericidal effects correlate with time above the MIC (t>MIC).[39] Pharmacokinetic modelling suggests that the current lower recommended paediatric doses (100mg/kg/day, Table 2 & Table 3) are insufficient for achieving target t>MIC for term neonates; and the corrected gestational age and body weight in neonates are the key explanatory variables for fosfomycin's PK.[39]

Previous research investigating the oral bioavailability of fosfomycin in adults documented a range between 34 and 58%.[40,41] Absorption is via the small intestine and is reduced by concurrent administration with food (37% when fasting versus 30% with food), thus, C_{max} that is higher under fasting conditions.[42]

Justification for the study

Neonatal sepsis has a high risk of morbidity and mortality. The current WHO and national guidelines recommend antibiotics to which resistance is reported in neonatal populations, although the available data is limited. Research on alternative empirical regimens for neonatal sepsis which are affordable, safe and cost-effective, with a step-down oral option, is needed. AMR is an issue of global public health concern and is one of the WHO's global health priority areas.[43] Understanding the benefits, risks, MIC capacity and PK of fosfomycin will influence global policy on the case management of neonates with sepsis in Kenya and international settings.

State the Null Hypotheses

i. The pharmacokinetics of the currently recommended various doses of IV and PO fosfomycin are unsuitable for treating neonates.
ii. Fosfomycin administration is not associated with altered plasma sodium in neonates.
iii. Fosfomycin does not inhibit growth of more than 25% of archived isolates of Enterobacteriaceae that express an ESBL phenotype in vitro.

Objectives

i. *General Objectives*

To improve the understanding of fosfomycin pharmacokinetics and safety amongst newborns aged ≤28 days hospitalized with clinical sepsis and provide detailed information regarding the antimicrobial susceptibility of local invasive bacteria to fosfomycin.

ii. *Specific Objectives*

- To estimate the PK disposition parameters of IV and PO fosfomycin in neonates
- To assess the safety of fosfomycin, particularly with regard to possible elevation of sodium after 48 hours of IV fosfomycin administration in neonates
- To estimate the oral bioavailability of fosfomycin in neonates
- To generate preliminary data on the safety of oral fosfomycin in neonates
- With the above information, generate a recommended dosing schedule for future IV and PO fosfomycin efficacy trials
- To collect information of the tolerability of oral fosfomycin

iii. *Secondary Objective*

- To gain information regarding susceptibility patterns of local bacterial species to fosfomycin

Study Design

A safety and pharmacokinetic study among neonates admitted to a rural hospital in Kenya and eligible for IV antibiotics under current national guidelines. Approximately 120 patients will be randomized 1:1 to standard-of-care antibiotics plus a 7-day course of fosfomycin (n=60); or standard-of-care (n=60) antibiotics only (ampicillin 50mg/kg twice daily and gentamicin [3mg/kg for babies <2kg or 5mg/kg for babies >2kg] once daily for 7 days, as per Kenyan guidelines).

For the group receiving fosfomycin, fosfomycin will initially be administered IV for at least 48 hours together with standard care (ampicillin + gentamicin). Then, once babies are tolerating oral feeds and clinically improved, fosfomycin will be changed to oral administration to complete a total of 7 days of fosfomycin (or until the baby is discharged). Two PK samples will be taken after each of the first IV and oral doses, with sampling times allocated within possible early (5, 10 or 60 minutes) and late (2, 4 or 8 hours) time-points after starting the IV and PO formulations; for the babies who remains inpatient at D7, a 5th PK sample will be collected to check for long term accumulation together with a laboratory safety sample. PK sampling timepoints may be subject to change based on emerging data. In total, four PK blood samples of 0.5ml each will be drawn from each participant, plus a fifth sample collected at 7 days. Haematology, biochemistry and electrolytes (a commonly performed investigation for babies with sepsis) will be checked at 48 hours and 7 days (if the baby is still inpatient) in both groups at the same time that the PK sample is collected. Daily observations until discharge and AEs up to a follow-up visit at day 28 will be recorded.

For the group receiving standard-of-care only, daily observations until discharge and AEs up to a follow-up visit at day 28 will be recorded.

The Day 28 visit will ideally be conducted within 7 days of the target date but if this is not practical the visit may be later than this. The visit date will be recorded.

Study site:

Kilifi County Hospital, Kilifi, Kenya

Definition of Study Population:

Criteria for inclusion of subjects (for pharmacokinetics):

i. Neonates defined as:

- Age 0 to 28 days inclusive
- Weight >1500g

- Born (an estimated) >34 weeks gestation (calculated as per the Ballard Maturational Assessment)
- Admitted to hospital and eligible to receive IV antibiotics, according to national guidelines

All neonates presenting to Kilifi County Hospital meeting the above criteria will be screened for inclusion in a systematic recruitment process.

ii. Criteria for exclusion of subjects

- Baseline sodium level ≥150mmol/L
- Baseline creatinine ≥150 micromol/L
- Presenting with severe (grade 3) Hypoxic Ischaemic Encephalopathy (HIE), defined as per Sarnat and Sarnat[44] as a stuporous, flaccid infant (with or without seizure activity) with suppressed brainstem and autonomic functions and absent reflexes
- Requiring cardiopulmonary resuscitation on admission
- Jaundice requiring exchange transfusion
- Admitted as a transfer after an overnight inpatient stay at another hospital
- Known allergy or contraindication to fosfomycin
- A specific clinical indication for another class of antibiotic (other than the nationally recommended standard-of-care)
- More than 24 hours after initiating ampicillin plus gentamicin (one dose), which allows for administration of these first-line antibiotics not to be delayed by study procedures
- Concurrent participation in another clinical trial
- Attending clinician's judgement that the child is so severely ill that adequate communication about the study with the parent or legal guardian is not possible.
- Not planning to remain resident in the County for the next 28 days.
- Lack of consent

iii. Rationale for animal use and justification for animal species chosen

- Not applicable

Sampling

i. Sample size determination:

a. For Pharmacokinetics:

Sample size has been calculated to ensure that the following PK parameters can be estimated with sufficient precision such that a dose schedule can be recommended for a future efficacy trial:

- Clearance (CL)
- Central volume (V)
- Oral Bioavailability (F)

Precision limits were set to 20%, and the power to estimate parameters with 95% confidence intervals within these limits was assessed by simulation-estimation. The simulation model consisted of an adult disposition model, with age and size scaling down to neonates, with added first-order absorption and assumed bioavailability. Six sampling time points were chosen to cover the dose intervals (3 early, 3 late) and the simulated population was randomly assigned age and weight combinations across the range expected for neonates. Parallel and crossover (IV/oral) designs were considered, with a range of 2-4 samples per patient. Power for sample size was greatest for the cross-over design. For the cross-over design, a minimum of 45 subjects contributing the complete set of 4 samples each (allocated early and late sample following the first IV and PO dose) are required to provide power of >85% to estimate all parameters within 20% precision limits. We estimate that up to 25% of subjects will not provide complete sample sets (either due to missing samples or withdrawal), so plan to recruit approximately 60 subjects to ensure 45 complete sample sets. If all 60 subjects provide complete sample sets, power would rise to 96%. Recruitment will continue until 45 patients in fosfomycin arm have a complete set of PK samples.

b. For Plasma Sodium

We have reviewed the data of (n=1,785) neonates >1500g admitted to Kilifi County Hospital (2015/6), which indicate a sodium mean and standard deviation of 139mmol/L and (SD 7.6, range 106 to 198mmol/L). 7.4% of babies had an admission sodium of >150mmol/L (our exclusion criterion). Excluding these babies, the mean sodium level in (the remaining n=1,653) babies was 137mmol/L (SD 5.2). With a minimum of 45 in each group

(PK versus standard-of-care), the study has >85% power to detect a difference in sodium of 5mmol/L between groups.

The sample size is not intended to be powered for antimicrobial efficacy or clinical outcomes.

c. For MIC of stored bacterial isolates and bowel flora:

Susceptibility of fosfomycin and other antibiotics is already being tested as per protocol SSC-1433. We will test n=200 invasive isolates from paediatric patients collected within the last 5 years, calculated based on >80% power to discriminate a non-susceptible proportion of up to 17% from a hypothetical proportion of 25% (one-sided). This is selected as a proportion which would render fosfomycin ineffective for introduction should this level of non-susceptibility be found. We shall then investigate fosfomycin susceptibility on other (ESBL-negative) Gram-negative isolates, and Gram-positive pathogens. For assessment of susceptibility patterns in bowel flora, we will systematically assess all admission and discharge nappy swabs from those babies included in the study.

ii. Study Endpoints:

a. Primary Endpoint:

Estimation of the pharmacokinetic disposition and absorption parameters of IV and oral fosfomycin in neonates with clinical sepsis with sufficient precision such that a dose schedule can be recommended for a future efficacy trial

b. Secondary Endpoint(s):

Difference between the groups in mean 48-hour plasma sodium concentrations

Difference between the groups in mean 7-day plasma sodium concentrations

Difference between groups in the rate of adverse events (any grade) to 28 days after enrolment in the study.

Procedures:

i. Analysis of Bacterial Isolates:

Isolates collected from nappy swabs will be subcultured and tested for fosfomycin susceptibility using disk diffusion (E. coli) and agar dilution (all isolates).[45] For disc diffusion, commercially available discs containing 200ug fosfomycin and 50mg of glucose-6-phostpate will be used. MICs will be determined by the agar dilution method using Mueller-Hinton agar supplemented with 25ug/m of glucose-6-phosphate and doubling concentrations of fosfomycin. The MIC will be recorded as the lowest concentration inhibiting visible growth. Plates will be incubated in ambient air at 35°C for 16 to 18 hours. Testing will be performed in duplicate, and mean MICs / zone diameters interpreted using EUCAST breakpoints (http://www.eucast.org/clinical_breakpoints/).

ii. Pharmacokinetic Study - Enrolment Procedure:

All neonates presenting to Kilifi County Hospital will be systematically screened to assess their eligibility in meeting the inclusion criteria and consent requested from the parent / guardian. Sequential study numbers will be generated according to a blocked randomization from a list of random block sizes created before the study begins. Randomization cards linking allocation (to standard care plus fosfomycin or standard care alone) to study number will be placed in sealed opaque envelopes by the study sponsor. On enrolment, infants will be allocated study numbers sequentially, thus randomly allocating the two groups. Since this is an open-label study, once an envelope is opened, the randomization card will be securely attached to the patient's CRF.

iii. Consent Process

Consent will be required for all data and samples taken for research purposes. Consenting will be done in a private room by study clinicians or trained field assistants, with the opportunity to ask questions and discuss concerns. Informed consent will be administered in a language that the parent/guardian best understands (English, Swahili or Giriama) after assessment of his/her literacy level. This will be done in the paediatric ward or high dependency unit once the decision to admit has been made. Whilst giving written consent parents/legal guardians will be able to agree to consent separately for participation in the study, storage of data and samples for future research, and export of samples for the PK assay that cannot currently be conducted in Kenya.

iv. Data Collection

For all participants, a study-specific case report form (CRF) will be used from the time of enrolment and captured information will be entered into a database. The CRF will include a daily standardized record of

clinical progress and drugs administered which will also be entered into a database. At discharge, the date, vital status and weight will be recorded.

v. Data Management and Analysis:

After the CRF has been completed and monitored by the clinical monitor, CRFs will be collected and data will be entered onto a validated password protected Openclinica database. Data will be kept confidential, with access restricted on password-protected computers, with regular secure backup. Any data transferred between Kenya and Europe will be emailed within password-protected encrypted files.

Analysis of fosfomycin and major metabolite concentration in plasma will be undertaken by Liquid Chromatography Mass Spectrometry (LC-MS) using validated methods in the GCP/GLP compliant laboratory in Analytical Services International Ltd, St George's Hospital, London, UK. Where possible, (scavenged) PK for penicillin and gentamicin will be measured using the same sample. Analysis will undertake by Dr Karin Kipper, Dr Joe Standing and Mr Martin Ongas, who will be trained on the techniques whilst running the analyses.

PK modelling and dosing simulations will be undertaken by non-linear mixed-effects modelling using NONMEM® software. The volume of distribution, half-life, clearance and trough levels of bound and unbound drug, and active metabolites will be estimated with 95% confidence intervals. Periods with concentrations above the CLSI, EUCAST and BSAC susceptibility breakpoints will be estimated. We will examine the effects of covariates including age, weight, and concurrently measured plasma sodium, potassium, and liver enzymes. Monte-Carlo simulations will be performed to determine the appropriate dosage and frequency of administration.

vi. Clinical Care

Alongside protocol specific training, the study team will also conduct refresher training for clinicians on the current national guidelines for managing neonates presenting with presumed sepsis. Neonates will be reviewed every day by study clinicians, working together with the hospital team. All adverse events will be documented and reported. All SAEs (whether or not the event is considered related to medication) are to be reported as described in section on safety reporting.

All other aspects of care will be provided according to national guidelines. Should a patient require second-line antimicrobials (third-generation cephalosporins), they will not be removed from the study as this will not impact the fosfomycin PK data.

vii. Study Treatment:

Fosfomycin is a peptidoglycan antibiotic which has bactericidal effects. There will be two formulations of fosfomycin utilized in this study:

- Fosfomycin 40 mg/ml powder for solution for infusion
- Fosfomycin powder for reconstituted suspension (250mg/5ml)

Preparation will be in accordance with manufacturer's instructions. Further details regarding treatment dispensing, administration and accountability is documented in Appendix D. Training will be provided to all staff involved in its administration.

viii. Timing of Assessments:

A schedule of events identifying the timing of required assessments and investigations is documented in Figure 2:

Figure 2: Schedule of Events

Note: If a Lumbar Puncture is clinically indicated after commencing fosfomycin, a scavenged PK sample will be obtained from the CSF

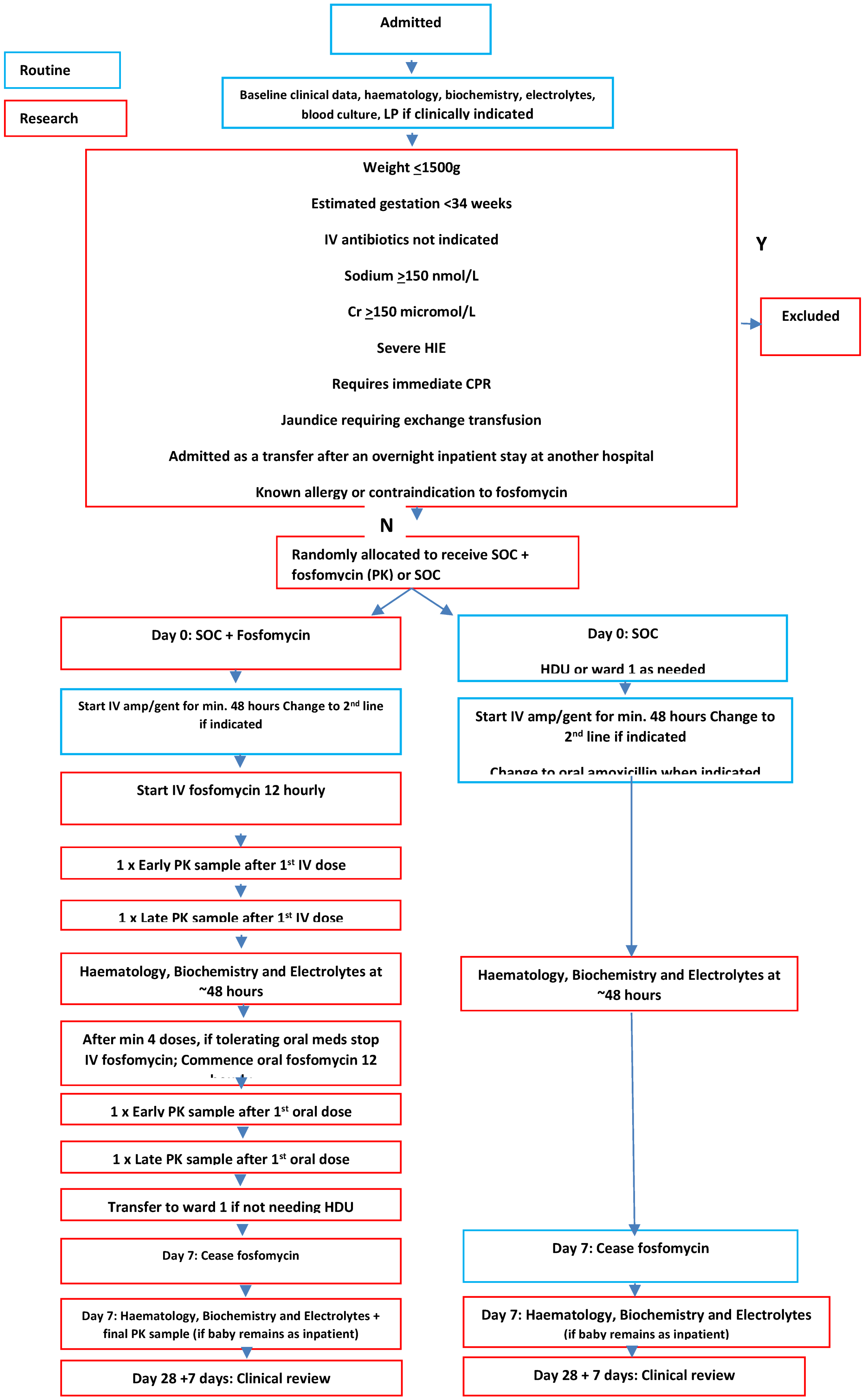
Admitted
Routine
Research
Baseline clinical data, haematology, biochemistry, electrolytes, blood culture, LP if clinically indicated
Weight <1500g
Estimated gestation <34 weeks
IV antibiotics not indicated
Sodium >150 nmol/L
Cr >150 micromol/L
Severe HIE
Requires immediate CPR
Jaundice requiring exchange transfusion
Admitted as a transfer after an overnight inpatient stay at another hospital
Known allergy or contraindication to fosfomycin
Y
Excluded
N
Randomly allocated to receive SOC + fosfomycin (PK) or SOC
Day 0: SOC + Fosfomycin
Start IV amp/gent for min. 48 hours Change to 2nd line if indicated
Start IV fosfomycin 12 hourly
1 x Early PK sample after 1st IV dose
1 x Late PK sample after 1st IV dose
Haematology, Biochemistry and Electrolytes at ~48 hours
After min 4 doses, if tolerating oral meds stop IV fosfomycin; Commence oral fosfomycin 12
1 x Early PK sample after 1st oral dose
1 x Late PK sample after 1st oral dose
Transfer to ward 1 if not needing HDU
Day 7: Cease fosfomycin
Day 7: Haematology, Biochemistry and Electrolytes + final PK sample (if baby remains as inpatient)
Day 28 +7 days: Clinical review
Day 0: SOC
HDU or ward 1 as needed
Start IV amp/gent for min. 48 hours Change to 2nd line if indicated
Change to oral amoxicillin when indicated
Haematology, Biochemistry and Electrolytes at ~48 hours
Day 7: Cease fosfomycin
Day 7: Haematology, Biochemistry and Electrolytes (if baby remains as inpatient)
Day 28 + 7 days: Clinical review

ix. Pharmacokinetics Procedures:

a. Baseline Assessments:

Following informed consent, study clinical officers will prescribe both routine standard-of-care antibiotics (ampicillin 50mg/kg twice daily, and gentamicin [3mg/kg for babies <2kg, 5mg/kg for babies >2kg] once daily) and, for the PK group, fosfomycin (100mg/kg every 12 hours, initially IV). Findings from history and examination, and standard admission investigations (CBC, biochemistry and electrolytes) will be collected at baseline, D2 and D7 (if baby remains inpatient). A blood culture will be performed at admission +/- lumbar puncture; from which a scavenged PK sample will be sent for analysis if sufficient CSF remains (if there is a clinical indication for this to occur following the administration of IV fosfomycin). In order to assess antimicrobial resistance that is bought into hospital and that which has been acquired on the ward, an antimicrobial susceptibility profile will be determined for rectal carriage of resistant isolates by collecting a nappy swab at admission and discharge. This will enable determination of the effect of carriage of antimicrobial resistance following treatment with fosfomycin.

b. Pharmacokinetic Assessments:

The first dose of fosfomycin will be followed by the collection of two PK samples at allocated times: one early (during 5, 10- or 60-minutes post-dose) and one late (during 2, 4- or 8-hours post-dose). After a minimum of 48 hours (or 4 IV doses), when tolerating oral medications, fosfomycin will be changed to oral and prescribed at the same dose (100 mg/kg every 12 hours). Following the first oral dose, one early and one late PK sample will again be obtained. For those who remain as inpatients, a PK sample of 0.5ml will be obtained together with a day 7 safety assessment. This will involve 5 plasma PK samples in total per patient, estimated as requiring an upper limit of 0.5 mL/sample (resulting in 2.5mL total study PK sample collection).

As per usual clinical procedures, blood for haematology, biochemistry and electrolytes will be drawn at ~48 hours (co-ordinated with the first post-oral PK sample time-point for patients who step-down to oral fosfomycin at this point) and again at 7 days (for those who remain as inpatients in each study group).

All participants will receive a structured daily review including clinical status and current treatment. Standard antibiotics may be altered in line with the results of a blood culture, which is currently done routinely for clinical care (fosfomycin will be continued). Planned follow-up for clinical review will be done at 28 days and will be conducted within 7 days of the target date but if this is not practical the visit may be later than this. Participant fares and compensation for lost work time will be provided at standard rates for this visit. Participants may also attend the ward in case of significant illness between discharge and day 28.

**Drug dose or sample**	**Target times⁺**
Dose: 1st Ampicillin	After admission investigations
Dose: 1st Gentamicin	After admission investigations
Dose: 1st Fosfomycin IV	After admission investigations
SAMPLE 1*: ONLY ONE OF:	
5 minutes	5 minutes after 1st fosfomycin intravenous dose
30 minutes	30 minutes after 1st fosfomycin intravenous dose
60 minutes	60 minutes after 1st fosfomycin intravenous dose
SAMPLE 2: ONLY ONE OF:	
2 hours	2 hours after 1st fosfomycin intravenous dose
4 hours	4 hours after 1st fosfomycin intravenous dose
8 hours	8 hours after 1st fosfomycin intravenous dose
Dose: 2nd Ampicillin	12 hours after 1st ampicillin dose; continuing 12 hourly
Dose: 2nd Fosfomycin IV	12 hours after 1st fosfomycin dose
Dose: 2nd Gentamicin	24 hours after 1st Gentamicin dose; continuing daily
Dose: 3rd Fosfomcycin IV	24 hours after 1st fosfomycin dose
Dose: 4th Fosfomycin IV	36 hours after 1st fosfomycin dose
**--Change to PO fosfomycin--**<br>1st dose Fosfomycin PO	48 hours after 1st fosfomycin dose; when tolerating oral medication and without signs of sepsis (otherwise continue IV 12 hourly)
SAMPLE 3: ONLY ONE OF: (collected alongside biochemistry)	
5 minutes	5 minutes after 1st fosfomycin oral dose
30 minutes	30 minutes after 1st fosfomycin oral dose
60 minutes	60 minutes after 1st fosfomycin oral dose
SAMPLE 4: ONLY ONE OF:	
2 hours	2 hours after 1st fosfomycin oral dose
4 hours	4 hours after 1st fosfomycin oral dose
8 hours	8 hours after 1st fosfomycin oral dose
Dose: 2nd Fosfomycin PO	12 hours after 1st fosfomycin oral dose;<br>continuing 12 hourly for up to 7 days
SAMPLE 5: (collected alongside biochemistry)	7 days after 1st fosfomycin intravenous dose

***Note: Subjects will be allocated to only one of each sampling timeframe**

⁺ **PK** timepoints may be subject to change based on emerging data

Samples will be collected into heparinised tubes, centrifuged and the separated plasma stored at minus 80°C. Haematology, Biochemistry and Electrolytes will be measured in real time, and results returned immediately to assist clinical care.

If a lumbar puncture is conducted and processed in the laboratory for clinical reasons, residual CSF will be stored at minus 80°C to assess CSF antimicrobial penetration.

Samples will be securely stored on site in the KEMRI-CGMRC in Kilifi. Following export approval by SERU, samples will be packaged and shipped according to IATA regulations by a reputable courier, such as World Courier to Analytical Services International Ltd. At St. George's Hospital, London and University College London for analysis. Pharmacokinetic assay results will be returned to Kenya. Samples may be stored for up to 5 years. Additional analyses beyond those described in this protocol: assaying antimicrobial drug levels and haematology, biochemistry/liver function will first require further approval by SERU. Remaining samples will be destroyed by incineration according to GCP after 5 years.

	SCREENING	BASELINE	←--TREATMENT PHASE---→					FOLLOW-UP
**PROCEDURES**			Daily until discharge	D0	48 hours	Day 7	Discharge	Day 28 + 7 days
**Informed Consent**	X							
**Demographics**	X							
**Medical History**	X		X				X	X
**Physical Examination**	X		X				X	X
**Vital Signs (incl. wt)**	X		X				X	X
**PK Sampling**				X	X[#]	X[^]		
**Haematology, Biochemistry and Electrolytes**	X				X[#]	X[^]		
**Eligibility Assessment**	X							
**Allocation**		X						
**Dispensing Trial Drugs**		X	X	X				
**Tolerability Questionnaire**						X	X	
**Nappy Swab**		X					X	
**Adverse Event Assessment**	X	X	X	X	X	X	X	X

*Or when tolerating oral medication

[#]Acceptable time point: within 12 hours (either side) of 48 hours since commencing IV fosfomycin

[^]Acceptable time point: within 24 hours (either side) of day 7 since commencing IV fosfomycin

x. Provisions for data verification and validation

Clinical data will be collected prospectively, supervised by a consultant paediatrician investigator, and overseen by the coordinating team in Kilifi. Data will be monitored for integrity and completeness weekly by the data manager. The study will be monitored by the Sponsor. For the PK study, a comprehensive study initiation visit will be done at which all staff will be trained in protocol specific procedure and check that all trial logistics are in place. A site initiation visit, then routine monitoring will be conducted during PK study. The monitor's role is part of the quality system that will ensure that all participants have duly completed informed consent; entries relating to eligibility, consent and data collected on the CRF are source-verified; and that staff on the study are following standard operating procedures (SOPs) which are in accordance with the protocol.

Clinical Trial Governance:

An independent DSMB will have an advisory role to safeguard the interests of trial's participants, investigators and sponsor; to assess the safety of the trial's intervention, and to monitor the trial's overall conduct, and protect its validity and credibility. Its recommendations will be addressed by the Sponsor to the study team. The DSMB operations will be facilitated by the Sponsor. The DSMB will usually be convened in a quarterly basis, by teleconference. The chair may also call for ad hoc or emergency meetings. The DSMB will report to the Sponsor usually within 2 weeks of a meeting, copied to the trial statistician. Unless the DSMB is recommending that the trial protocol be changed, the letter to the Sponsor should not usually reveal any confidential information. If the DSMB has serious concerns about a Study team decision, a meeting of these groups should be held, chaired by an external expert not directly involved with the trial.

Safety reporting

i. Assessment of Safety:

Neonates will be reviewed every day by study clinicians, working together with the hospital team. All adverse events will be documented and reported.

Safety will be assessed through routine monitoring of adverse events. In addition, evaluation of haematology and blood chemistry parameters, regular measurement of vital signs and physical examinations will be conducted as per the protocol and clinical indication. The frequency, severity, seriousness and causality assessments of AEs will be described, as well as frequency of SAEs or AEs that lead to treatment discontinuation. AEs will be collated for both patient groups in the CRF.

ii. Protocol Violations:

Any protocol violations will be reported to the Sponsor (DNDi), to an independent DSMB, and to the Ethics Committee and PPB.

iii. Laboratory examinations

Haematology parameters (CBC) will be analysed at screening, on days 2 and day 7 (if baby remains as inpatient) as is normally clinically indicated (plus any additional investigations as clinically indicated). Biochemistry and electrolytes parameters will be analysed at screening, on day 2 and day 7 (if baby remains as inpatient). Additional samples may be done if clinically indicated. Samples will be analysed at the adjacent KWTRP Clinical Trials Laboratory using standardized equipment. Approximately 0.5ml of blood will be collected for each study investigation.

Abnormal Lab parameters will be assessed for clinical significance. If the abnormality is judged clinically significant, an adverse event must be reported.

iv. Adverse event definitions and reporting

The definition of adverse events, adverse drug reactions, serious adverse event/reactions, or suspected unexpected serious adverse reactions (SUSARs) is outlined below:

**TERM**	**DEFINITION**
Adverse Event (AE)	Any untoward medical occurrence in a patient or clinical trial participant administered a medicinal product and which does not necessarily have a causal relationship with this treatment[A]
Adverse Drug Reaction (ADR)	A response to a (investigational or authorized) medicinal product which is noxious and unintended.
Serious Adverse Event (SAE) or<br>Serious Adverse Reaction (SAR)	Any untoward medical occurrence or effect that at any dose which:<br>Results in death<br>Is life-threatening<br>Requires hospitalization or prolongation of existing hospitalization<br>Results in persistent or significant disability or incapacity<br>Is a congenital anomaly or birth defect<br>Is considered medically important/clinically significant[C]
Non-Serious Adverse Event /<br>Non-Serious Adverse Drug Reaction	An adverse event or adverse drug reaction that does not meet the criteria listed above for a serious adverse event/serious adverse drug reaction

TERM	DEFINITION
Unexpected Adverse Reaction (UAR)	An adverse reaction, the nature or severity of which is not consistent with the SPC for Fosfocina, suspension (250mg/5ml) Laboratorios ERN; or Fomicyt, 40mg/ml powder for solution for infusion, InfectoPharm
Suspected Unexpected Serious Adverse Reaction (SUSAR)	An adverse reaction that is unexpected (not consistent with the SPC for Fosfocina suspension [250mg/5ml] Laboratorios ERN; or Fomicyt, 40mg/ml powder for solution for infusion, (InfectoPharm) and meets the definition of a serious adverse event/reaction

Adverse event:

Any untoward medical occurrence in a patient or clinical trial participant administered a medicinal product and which does not necessarily have a causal relationship with this treatment.

An AE can therefore be any unfavourable and unintended sign (including an abnormal laboratory finding), symptom, or disease temporally associated with the use of an investigational medicinal product (IMP), whether considered related to the IMP.

What is not an AE?

- Medical conditions present at the initial study visit, that do not worsen in severity or frequency during the study, are not considered as AE.
- Lack of efficacy of the IMP is not considered as AE.

Laboratory/procedures abnormalities considered as an AE:

Laboratory/procedures abnormalities (or worsening in severity or frequency of pre-existing abnormalities) should be assessed as “clinically significant” if they meet AT LEAST ONE of the following conditions:

- The abnormality suggests a disease and/or organ toxicity AND this abnormality was not present at the screening visit or is assessed as having evolved since the screening visit
- The abnormality results in discontinuation of the study drug
- The abnormality requires medical intervention or concomitant therapy

When reporting an abnormal laboratory/procedure result, a clinical diagnosis should be recorded rather than the abnormal value itself, if available (for example, “anaemia” rather than “low haemoglobin”). For every laboratory assessment, the investigator will evaluate if the lab test is normal or abnormal. If abnormal, the investigator will assess if this finding is clinically significant or not. If a lab parameter is abnormal and clinically significant, it should be reported as an adverse event, after comparison with the previous value (AE).

v. Eliciting Adverse Event information

The investigator will report all directly observed AEs and all AE spontaneously reported by the parents/guardians using concise medical terminology. In addition, at follow-up visit the parents/guardians will be asked a a generic question such as “Since you were discharged from hospital, has your child had any health problems?” Information on AEs will be evaluated by a physician.

All AEs must be evaluated with regards to severity (Clinical intensity), causality (with each study drug) and for seriousness (regulatory definition for reporting).

vi. Adverse Event reporting period

The adverse events reporting period begins upon subject enrolment in the trial (after signature of informed consent and ends 28 days (4 weeks) after the first dose of study drug(s) is administered.

vii. Anticipated Events in neonatal setting:

Anticipated events listed below are relatively common in this patient group due to low birth weight, possible birth asphyxia and concomitant disease processes.[46] Anticipated events in this patient population (babies under 28 days) defined in the table below will be recorded and reported in the CRF session “Anticipated Events”.

These anticipated events must be assessed by the investigators with regards to the definition of adverse event, in the context of neonatal setting.

Anticipated events associated with neonatal setting, but which are **not assessed as "AEs** "(not an untoward medical occurrence taking into account the newborn pre-exiting conditions and common neonatal setting/conditions**)** will be reported on the CRF **but not as AEs (or SAEs).**

Anticipated events which are **more severe or more frequent than expected in this neonatal setting** will be reported as **AEs, and assessed for severity, causality and seriousness as any other AE**. If classified as AEs, they must be reported as AEs in the CRF and, if matching any seriousness criteria, on CRF AE and SAE form.

All anticipated events, not matching the AE definition will be provided to DSMB as part of the quarterly line listings reviewed by the DSMB and then forwarded to SERU/OXTREC and PPB.

They will not be reported in an expedited manner unless they are assessed as both AEs and serious.

**ANTICIPATED EVENTS IN NEONATAL SETTING**
**Necrotising enterocolitis (diagnostic radiological/surgical changes)**
**Intracranial abnormality on cranial ultrasound scan (parenchymal haemorrhage or focal white matter injury)**
**Patent ductus arteriosus**
**Pulmonary haemorrhage**
**Severe anaemia requiring transfusion (due to ABO incompatability, prematurity or haemorrhage)**
**Jaundice requiring phototherapy or exchange transfusion**
**Congenital birth defect diagnosed during admission**
**Fracture secondary to birth trauma**
**Apnoea**
**Infection (positive blood culture with clinical signs)***
**Persistent derangement of liver function tests (beyond 36 weeks CGA)**
**Abnormal muscle tone, posturing or convulsions (secondary to suspected birth asphyxia)**
**An episode of Hypoglycaemia (defined as per the World Health Organization, ≤2.6mmol/L)[47]**

* Both infections other than condition for inclusion in trial and, in case of infections linked to the sepsis episode that is the reason of the participant's inclusion in the trial, worsening (e.g. septic shock) and relapse.

viii. Adverse Event Recording:

All AEs will be recorded on the AE CRF and reported as a listing at the end of the study.

In the CRF, a given AE will be recorded one time per subject, and the severity will be assessed and recorded as the maximum level reached during an episode. If several distinct episodes of the same condition occur, their number will be recorded in the CRF. In addition, the causal relationship between the onset of each AE and each study drug IMP will be assessed.

Information on adverse events will be evaluated by a physician. Each adverse event will be classified by the investigator for severity (ix), causality (x) and also as serious or non-serious (xi). This "seriousness" classification will determine the regulatory reporting procedure (reporting form and timelines) for the AE.

ix. Grading of Adverse Event severity

Toxicities and adverse events will be graded for severity to describe the maximum severity of the adverse event according to the DAIDS grading scales (version 2.1, March 2017; Available at: https://rsc.tech-res.com/docs/default-source/safety/daids-ae-grading-table-mar2017.pdf?sfvrsn=6).

In case of AEs that are not described in the DAIDS AE grading system, the investigator will use the terminology "mild", "moderate", "severe" or "life-threatening" to describe the maximum severity of the adverse event. These severity grades are defined as follows:

MILD	Does not interfere with subject's usual functions
MODERATE	Interferes to some extent with subject's usual functions

SEVERE	Interferes significantly with subject's usual functions
LIFE-THREATENING	The subject is at risk of death at the time of the AE it does not refer to an AE that hypothetically might have caused death if more severe.

It is to be noted there exists a distinction between severity and seriousness of adverse events. A severe adverse event is not necessarily a serious event.

x. Adverse Event causality assessment

For both serious and non-serious AEs, the investigator is required to assess the causal relationship between the AE and the study drug, i.e. to determine whether there exists a reasonable possibility that the study drug caused or contributed to the adverse event, or evidence to suggest a causal relationship.

To help investigators with the decision binary tree in the evaluation of causality, as per the CIOMS VI group recommendation, the investigators will consider the following before reaching a decision:

- Medical history
- Lack of efficacy/worsening of existing condition
- Study medications
- Other medications (concomitant or previous)
- Withdrawal of study medication, especially following trial discontinuation / end of study medication
- Erroneous treatment with study medication (or concomitant)
- Protocol related procedure

The terms for reporting are:

Definitely related	The adverse event and administration of IMP are related in time, and a direct association can be demonstrated.
Probably related	The adverse event and administration of IMP are reasonably related in time, and the adverse event is more likely explained by study agent than other causes
Possibly related	The adverse event and administration of IMP are reasonably related in time, and the adverse event can be explained equally well by causes other than IMP
Unlikely related	A potential relationship between IMP and the adverse event could exist (i.e. the possibility cannot be excluded), but the adverse event is most likely explained by causes other than the IMP
Not related	The adverse event is clearly explained by another cause not related to the IMP

Adverse Drug Reaction (ADR):

A response to a (investigational or authorized) medicinal product which is noxious and unintended.

Response in this context means that a causal relationship between a medicinal product and an AE is at least a reasonable possibility.

The definition implies a reasonable possibility of a causal relationship between the event and the investigational medicinal product. This means that there are **facts (evidence) or arguments** to suggest a causal relationship (see definition of causality below).

With regards to safety reporting definition, “not related” corresponds to “not related, unlikely related” and “related” (AE related to any study drug at any dose) corresponds to “possibly related, probably related and definitely related”.

xi. Grading of Adverse Event seriousness

A serious Adverse event (SAE) is an Adverse Event (AE) which:

- **results in death**

i.e. causes or contributes to the death.

- **is life-threatening**

in this context refers to an AE in which the patient was **at immediate risk of death at the time of the AE**; it does not refer to an AE that hypothetically might have caused death if more severe.

- **requires in-patient hospitalization or prolongation of existing hospitalization**

i.e. the AE requires at least an overnight admission or prolongs a hospitalization beyond the expected length of stay. Hospital admissions for surgery planned before study entry, for social reasons, for any elective surgery (i.e. plastic surgery) or for normal disease management (including treatment adjustment) are NOT to be considered as SAE according to this criterion (i.e. if the protocol or the standard management of the disease under study requires planned hospitalization).

- **results in persistent or significant disability or incapacity**

i.e. the AE resulted in a substantial disruption of the subject's ability to conduct normal activities.

- **is a congenital anomaly / birth defect (Not applicable in this study)**

i.e. an AE outcome in a child or foetus of a subject exposed to the Investigational Medicinal Product (or marketed medicinal product (Note: to be only added for marketed drug)) before conception or during pregnancy.

- **is an important medical event, i.e. is medically significant**

Medical and scientific judgment should be exercised in deciding whether other situations should be considered serious events, such as important medical events that might not be immediately life-threatening or result in death or hospitalization but might jeopardize the patient or might require intervention to prevent one of the other outcomes listed above. In addition, any suspected transmission via a medicinal product of an infectious agent is also considered a SAE.

xii. Serious Adverse Event Reporting:

All Serious AEs (SAE) will be reported immediately (no later than within 24h of knowledge by the investigator) to the Sponsor, and within 24-48 hours of knowledge by the Investigator to the Ethics Committees (SERU and OxTREC) (whether or not the event is considered study drug-related), using the SAE report form. This includes a description of the event, onset date and type, duration, severity, relationship to each study drug, outcome, measures taken and all other relevant clinical and laboratory data.

In support of investigator, the Sponsor will report the SAE (using the SAE report form) to PPB and DSMB within 2 working days of awareness by investigator.

The initial report will be followed by submission of additional information (follow-up SAE form) as it becomes available.

Any follow-up reports will be submitted within 5 working days. In addition to documenting the SAE on the SAE report form, the SAE will also be documented on the AE CRF and all medications used to treat the SAE will be documented on the concomitant treatments CRF.

The sponsor will assess the causality of every SAE with each study drug.

In addition, the Sponsor will assess the expectedness of every SAE reported as "definitely/probably/possibly related" (therefore an adverse reaction) by the investigator or assessed the Sponsor as "related", with each study drug, and as per following definitions.

- Unexpected Adverse Reaction (UAR):

An adverse reaction, the nature or severity of which is not consistent with the SPC for Fosfocina, suspension (250mg/5ml) Laboratorios ERN; or Fomicyt, 40mg/ml powder for solution for infusion, InfectoPharm

- Suspected Unexpected Serious Adverse Reaction (SUSAR)

An adverse reaction that is unexpected (not consistent with the SPC for Fosfocina, suspension [250mg/5ml] Laboratorios ERN; or Fomicyt, 40mg/ml powder for solution for infusion, InfectoPharm) and meets the definition of a serious adverse event/reaction.

The Sponsor will report every SUSARs or Fatal SARs or other SAEs (CIOMS-I regulatory format) to PPB, Ethics Committees (SERU and OxTREC) and DSMB as follows:

- Fatal/Life-threatening SUSARs within 7 calendar days (+follow-up within 8 days) of knowledge by Sponsor
- Fatal SARs in 7 calendar days from knowledge by Sponsor
- Other SUSARs/SAEs within 15 calendar days of knowledge by Sponsor

xiii. Adverse event follow-up

All participants with AEs will receive treatment for those events and be followed up until they are resolved, or the investigator assesses them as chronic or stable, or the subject participation in the study ends (i.e., until a final report is completed for that subject) and care is ongoing.

In addition, all SAEs and those non-serious AEs assessed by the investigator as related (related/probably related/possibly related) to the investigational medication will continue to be followed even after the subject participation in the study is over. Such events will be followed until they resolve or until the investigator assesses them as chronic or stable. Resolution of such events will be documented on the CRF.

xiv. Withdrawal criteria:

A subject should be withdrawn from the study treatment if, in the opinion of the investigator, it is medically necessary, or if it is the wish of the parent/guardian. If a patient is withdrawn from the study before the full course of the treatment is completed, the physician will make all necessary arrangements to ensure that the subject receives the appropriate treatment for the relevant medical condition (i.e. with medication/s currently recommended by national guidelines). Should a patient require second-line or alternative antimicrobials, they will not be removed from the study as this will not impact the fosfomycin PK data.

If a subject does not return for the follow-up assessments at D28, every effort will be made to contact their parents/guardian. In any circumstance, every effort should be made to document subject outcome, if possible. If the subject withdraws consent, no further evaluations should be performed and no attempts should be made to collect additional data, except for safety data, which should be collected if possible and in accordance with consent.

If a subject is withdrawn from the study, the reason will be noted on the CRF. If a subject is withdrawn from the study because of a treatment limiting adverse event, thorough efforts will be made to clearly document the outcome of AE.

Data Safety Monitoring Board:

A Data Safety Monitoring Board (DSMB), composed of 3 members independent of the investigator and sponsor, will be set up prior to study initiation. The DSMB monitors the study in order to ensure that harm is minimized, and benefits maximized for the study subjects. They will review the study data at pre-determined intervals and issue recommendations about the study. The data and intervals will be agreed prior to, or soon after, the study initiation and documented in the DSMB Charter.

Quality Assurance and Quality Control Procedures

i. Investigator's file:

The investigator must maintain adequate and accurate records to enable the conduct of the study to be fully documented and the study data to be subsequently verified. These documents include:

- Investigator's Site File
- Subject clinical source documents
- Screening / enrolment logs.

The Investigator's Site File will contain the protocol/protocol amendments, CRF and query forms, REC and regulatory approval with correspondence, sample informed consent, drug accountability records, staff curriculum vitae, authorization forms and other appropriate documents and correspondence.

ii. Case report forms (CRFs):

For all participants, a standardized daily record will be used from admission (enrolment), and the study specific information will be captured in the respective CRF, and subsequently into the trial database. At discharge, the date, vitals status and weight will be recorded. Clinical data will be collected prospectively, supervised by a consultant paediatrician investigator to ensure data completeness and accuracy. Data that are derived should be consistent with the source documents or the discrepancies will be explained. All CRF data will be anonymized

(identified by study patient number only). The study will be reviewed by the Sponsor CRA. Study monitors will raise queries on data discrepancies, and these will be corrected by the study investigator against verified source information.

The investigators will ensure the accuracy, completeness, legibility, and timelines of all data reported to the sponsor in the CRFs and any other additional information that is required. The investigator is responsible for keeping all consent forms, screening forms, CRF and the completed subject identification code list in a secure location.

iii. Source documents:
The verification of the CRF data will be by direct inspection of source documents. Source documents include subject hospital/clinic records, physician's and nurse's notes, appointment book, original laboratory reports, letters, and subject screening and enrolment logs. The Investigator / designee will record the date of each subject's visit together with a summary of their status and progress in the study. The investigator will maintain source documents for possible review and/or audit by DNDi, Ethics Committees and/or Regulatory Authorities.

Indicate the identification of any data to be recorded directly on the CRFs (i.e. no prior written or electronic record of data), and to be considered to be source data. (Chapter 6: Clinical trial protocol and protocol amendment(s) § 6.4.9, CPMP/ICH/135/95 Topic E6)

iv. Record Retention:
The sponsor will keep all study documents on file for at least 25 years after completion or discontinuation of the study, at a secure facility contracted by the DNDi Nairobi offices, within Kenya. After that period of time the documents may be destroyed with prior permission from DNDi, subject to local regulations. Should the investigator wish to assign the study records to another party or move them to another location, DNDi will be notified in advance.

v. Monitoring, audits and inspections:
The investigators will permit representatives of DNDi, designated clinical monitors and representatives of Ethics Committees or Regulatory Authorities to review all CRFs, medical records, laboratory work sheets and to assess the status of drug storage, dispensing and retrieval at any time during the study. The Investigator's File and corresponding source documents for each subject will be made available provided that subject confidentiality is maintained in accordance with local regulations. The monitoring, audits or inspections are for the purpose of verifying the adherence to the protocol and to ensure the study is conducted according to GCP. It is important that the investigators and other trial site staff are available at these visits.

vi. Protocol Amendments:
The principal investigator will ensure that the study protocol is strictly adhered to throughout, and that all data are collected and recorded correctly on the CRF. All protocol modifications must be documented in writing. Any protocol amendment must be approved and signed by the sponsor and the Principal investigator and is to be submitted to the appropriate IEC for information and approval in accordance with local requirements, and to regulatory agencies if required. Approval by IEC (and Regulatory Authority, if applicable) must be awaited before any changes can be implemented, except for changes necessary to eliminate an immediate hazard to trial subjects, or when the change involves only logistical or administrative aspects of the trial [e.g. change in clinical monitor[s], change of telephone number[s].
The protocol amendment can be initiated by either sponsor or by any Principal investigator. The investigator will provide in writing the reasons for the proposed amendment and will discuss with the medical coordinator and sponsor.

Data Sharing

The results of this work will be accessible with no financial barriers, by contributing to open source initiatives such as public databases and open access journals in a timely fashion. Permission to utilize anonymized data will be by application to the Data Governance Committee at CGMR-C who will ensure that appropriate ethical approval is in place for any new analysis. Explanation of this eventuality will be included in the participant Information and Consent Form (ICF).

Intellectual property

Any intellectual property rights that arise from the work will be safeguarded according to DNDi's IP policy and current KEMRI guidelines and the Industrial Property Act of 2001, sections 32, 58 and 80. The scientific and intellectual contributions of all persons involved in the research will be appropriately acknowledged in all publications and presentations arising from the work.

Ethical Considerations

The experimental protocol for this study has been designed in accordance with the general ethical principles outlined in the Declaration of Helsinki and ICH guidelines for Good Clinical Practice (International Committee for Harmonization). DNDi assures that it will comply with all applicable state, local and foreign laws for protecting the rights and welfare of human subjects. This protocol and any protocol amendments will be reviewed / approved by the KEMRI Scientific and Ethical Review Unit, Nairobi and the Oxford Tropical Research Ethics Committee, Oxford, UK. The study will be registered on www.clinicaltrials.gov.

Participation in research is voluntary and consent must be given with free will of choice, and without undue inducement. The parent/legal guardian must have sufficient knowledge and understanding of the nature of the proposed research, the anticipated risks and potential benefits, and the requirements of the research to be able to make an informed decision.

i. Human Subjects

First do no harm

ii. Risks

The study will be performed in a patient group who may potentially benefit from the treatment. Fosfomycin is licensed for use in neonates throughout Europe and is used to treat resistant UTIs among adults in Europe and the USA. Adverse events are reported to be rare, and monitoring of renal function, sodium and potassium levels should pre-empt major adverse clinical outcomes from occurring.

The risks of blood drawing include pain and thrombophlebitis. These will be minimized by careful aseptic technique according to a standard SOP. No more than 1ml/kg of blood will be drawn for research at any one time, and no more than a total of 2.5ml will be drawn for research during the entire study. DNDi has a specialist insurance policy in place which would operate in the event of any participant suffering harm as a result of their involvement in the performance of the study.

iii. Benefits to the Patients and Community as a whole

Additional clinical staff will be recruited and will undertake study duties and assist in care, adding to the staff available for clinical care on the wards. For babies who do not improve on the currently recommended standard-of-care, we will provide second-line antibiotics free of charge if they are required. This study contributes to knowledge informing the appropriate use of antimicrobials, thus benefiting the whole community both at a local and international level.

iv. Confidentiality

On enrolment, participants will be issued with a unique identifying number and names recorded on CRFs and in the database as initials only. All clinical data will be held confidentially, and the investigator will ensure that subjects' anonymity will be maintained, and their identities protected from unauthorized parties. No documents containing patient identity will be submitted to the sponsor. The investigator will maintain documents for submission to sponsor authorized representatives, and the subject's signed written consent forms, in strict confidence.

v. Community Engagement Strategy

Community engagement will commence prior to the study with a stakeholders meeting involving Ministry of Health County staff and relevant national Government authorities and policy makers. This will be facilitated by the study sponsor, with attendance by investigators. Ongoing community engagement will be through regular meetings with the community involving KEMRI-Community Representatives and County Government Health and hospital management teams. At these meetings, information and feedback will be given and received.

vi. Stakeholder information giving

We will engage key individuals whom patients may receive information from, including nurses and all ward staff. We will expand ongoing communication activities about research to include this study in order to support parents and guardians receiving information on the study, before being asked for consent.

vii. Individual informed consent process

Written consent will be required for all data and samples taken for research purposes. Consenting will be done in a separate area to ensure privacy and the opportunity to ask questions and discuss concerns in the paediatric ward, high dependency unit or in casualty once the decision to admit has been made. It is the responsibility of the investigator / designee to obtain written informed consent for each individual participating in this study, after adequate presentation of the aims, methods, anticipated benefits, and potential hazards of the study. The written

informed consent document will be translated into the local language (Swahili and Giriama). If needed, the person will be given time to discuss the information received with other members of the family before deciding to consent, providing this does not extend beyond the time identified for inclusion in the study as per the inclusion criteria. The subject or parent/guardian will be asked to provide written and signed consent. Parents or legal guardians will be able to consent separately for participation in the study, storage of data and samples for future research, and export of samples for investigations that cannot currently be conducted in Kenya. Where the attending clinician judges that the child is so severely ill that adequate communication with the parent or legal guardian is not possible, the child will be excluded from participation. If the parent /guardian is illiterate, a literate witness must sign (this person should have no connection to the research team and the sponsor, and, if possible, should be selected by the participant). The witness shall attest that they have provided information accurately to the parent/guardian and this was understood; a thumbprint of the parent/guardian must be provided to attest to this. If new safety information results in significant changes in the risk/benefit assessment, the consent form should be reviewed and updated if necessary. All subjects (including those already being treated) should be informed of the new information, given a copy of the revised form and give their consent to continue in the study.

viii. Training / support for those involved in community engagement and administering consent

Clinical officers, nurses and field workers will be trained in providing information and administering the consent procedure, following a standard operating procedure in the local language of participants, using didactic learning and role plays. In addition, all investigators will complete relevant courses in Good Clinical Practice ethical training specifically addressing research involving human subjects.

ix. Feedback of information

This study will be undertaken with the medical and nursing unit staff and the hospital consultants, who have been involved in its design and will be essential in its implementation. Information arising from the study will be fed back through hospital-wide meetings. Study results will be disseminated to study staff, hospital staff and local communities through meetings targeting respective groups. Results will also be published in peer-reviewed journals and presented at local and international meetings or conferences.

x. Animal Subjects

N/A

Expected Application of the Results

The results will contribute to both local and international knowledge regarding the appropriate use of antimicrobials in neonates with presumed sepsis and help in the design of a subsequent multi-centre, large-scale randomized clinical trial to determine the risks and benefits (in terms of mortality and antimicrobial resistance) of an updated antibiotic schedule, particularly where multidrug resistant bacteria are prevalent.

References

1 Lawn J, Blencowe H, Oza S, You D, Lee A. Every Newborn: Progress, Priorities, and Potential beyond Survival. Lancet 2014; **384**: 189–205.

2 Liu L, Oza S, Hogan D, et al. Global, regional, and national causes of child mortality in 2000-2013; with projections to inform post-2015 priorities: an updated systematic analysis. Lancet 2014; **385**: 430–40.

3 Shah D, Doyle L, Anderson P, et al. Adverse neurodevelopment in preterm infants with postnatal sepsis or necrotizing enterocolitis is mediated by white matter abnormalities on magnetic resonance imaging at term. J Pediatr 2008; **153**: 170–5.

4 The World Health Organization. Pocket Book of Hospital Care for Children: Guidelines for the Management of Common Illnesses with Limited Resources. 2013. http://apps.who.int/iris/bitstream/10665/43206/1/9241546700.pdf.

5 Downie L, Armiento R, Subhi R, Kelly J, Clifford V, Duke T. Community-acquired neonatal and infant sepsis in developing countries: efficacy of WHO's currently recommended antibiotics- systematic review and meta-analysis. Arch Dis Childhood 2013; **98**: 146–54.

6 Le Doare J. Heath, P. and Sharland, M. KB. Systematic review of antibiotic resistance rates among gram-negative bacteria in children with sepsis in resource-limited countries. J Paediatr Infect Dis Soc 2014; **4**: 11–20.

7 Folgori L, Livadiotti S, Carletti M, et al. Epidemiology and clinical outcomes of multidrug-resistant, gram-negative bloodstream infections in a European tertiary paediatric hospital during a 12-month period. Pediatr Infect Dis J 2014; **33**: 929–32.

8 Delhi Neonatal Infection Study (DeNIS). Characterisation and antimicrobial resistance of sepsis pathogens in neonates born in tertiary care centres in Delhi, India: a cohort study. Lancet Glob Heal 2016; **4**: e752–60.

9 Laxminarayan R, Matsoso P, Pant S, et al. Access to effective antimicrobials: A worldwide challenge. Lancet 2016; **387**: 168–75.

10 The World Health Organization. Critically important antimicrobials for human medicine. 3rd Revis. 2011.

11 Hendlin D, Stapley M, Jackson H, Wallick A, Miller A, Wolf F. Phosphonomycin. A new antibiotic produced by strains of Streptomyces. Science (80-) 1969; **166**: 122–3.

12 Michalopoulos AS, Livaditis IG, Gougoutas V. The revival of fosfomycin. Int J Infect Dis 2011; **15**: e732–9.

13 Falagas M, Vouloumanou E, Samonis G, Vardakas K. Fosfomycin. Clin Microbiol Rev 2016; **29**: 321–47.

14 Vardakas KZ, Legakis NJ, Triarides N, Falagas ME. Susceptibility of contemporary isolates to fosfomycin: a systematic review of the literature. Int J Antimicrob Agents 2016; **47**: 269–85.

15 Iarikov D, Wassel R, Farley J, Nambiar S. Adverse Events Associated with Fosfomycin Use: Review of the Literature and Analyses of the FDA Adverse Event Reporting System Database. Infect Dis Ther 2015; **4**: 433–58.

16 Baquero F, Hortelano JG, Navarro M, et al. Antibiotherapy of Serratia marcescens septicemia in children. Chemotherapy 1977; **23 Suppl 1**: 416–22.

17 Corti N, Sennhauser FH, Stauffer UG, Nadal D. Fosfomycin for the initial treatment of acute haematogenous osteomyelitis. Arch Dis Child 2003; **88**: 512–6.

18 Llorens J, Lobato A, Olay T. The passage of fosfomycin into the cerebrospinal fluid in children's meningitis. Chemotherapy 1977; **23 Suppl 1**: 189–95.

19 Careddu P, Borzani M, Scotti L, Varotto F, Garlaschi L, Fontana P. Treatment of lower urinary tract infections in children: single dose fosfomycin trometamol versus pipemidic acid. Chemioterapia 1987; **6**: 290–4.

20 Principi N, Corda R, Bassetti D, Varese LA, Peratoner L. Fosfomycin trometamol versus netilmicin in children's lower urinary tract infections. Chemotherapy 1990; **36 Suppl 1**: 41–5.

21 Varese LA. Trometamol salt of fosfomycin versus netilmicin: randomized multicenter study in children's lower urinary tract infections. Eur Urol 1987; **13 Suppl 1**: 119–21.

22 Nutrition A et al. for the EC on. Enteral Nutrient Supply for Preterm Infants: Commentary From the European Society for Paediatric Gastroenterology, Hepatology, and Nutrition Committee on Nutrition. Hepatol Nutr 2010; **50**: 85–91.

23 Group K et al. for the PNGW. Guidelines on Paediatric Parenteral Nutrition of the European Society of Paediatric Gastroenterology, Hepatology and Nutrition (ESPGHAN) and the European Society for Clinical Nutrition and Metabolism (ESPEN), Supported by the European Society of Paediatri. J Pediatr Gastroenterol Nutr 2005; **41**: S1–S4.

24 Nash P. Potassium and Sodium Homeostasis in the Neonate. Neonatal Netw 2007; **26**: 125–8.

25 Akçam M. Oral fructose solution as an analgesic in the newborn: A randomized, placebo-controlled and masked study. Pediatr Int 2004; **46**: 459–62.

26 Bueno M, Yamada J, Harrison D, et al. A systematic review and meta-analyses of nonsucrose sweet solutions for pain relief in neonates. Pain Res Manag 2013; **18**: 153–61.

27 Taylor CG, Mascaros E, Roman J, et al. Enteropathogenic E. coli gastroenterocolitis in neonates treated with fosfomycin. Chemotherapy 1977; **23 Suppl 1**: 310–4.

28 Rossignol S, Regnier C. [Fosfomycin in severe infection in neonatology]. Ann Pediatr (Paris) 1984; **31**: 437–44.

29 Guillois B, Guillemin MG, Thoma M, Sizun J, Monnery JL, Alix D. [Neonatal pleuropulmonary staphylococcal infection with multiple abscesses of the liver]. Ann Pediatr (Paris) 1989; **36**: 681–4.

30 Gouyon JB, Francois C, Semama D, Sandre D, Duez JM, Portier H. [Nosocomial Staphylococcus epidermidis and Staphylococcus aureus septicemias in neonates]. Ann Pediatr (Paris) 1990; **37**: 21–5.

31 Algubaisi S, Buhrer C, Thomale U-W, Spors B. Favorable outcome in cerebral abscesses caused by Citrobacter koseri in a newborn infant. IDCases 2015; **2**: 22–4.

32 Li G, Standing J, Bielicki J, et al. The potential role of fosfomycin in neonatal sepsis caused by multidrug resistant bacteria. Publ 2016.

33 Guggenbichler JP, Kienel G, Frisch H. [Fosfomycin, a new antibiotic drug (author's transl)]. Padiatr Padol 1978; **13**: 429–36.

34 Molina MA, Olay T, Quero J. Pharmacodynamic data on fosfomycin in underweight infants during the neonatal period. Chemotherapy 1977; **23 Suppl 1**: 217–22.

35 Bergan T. Degree of absorption, pharmacokinetics of fosfomycin trometamol and duration of urinary antibacterial activity. Infection 1990; **18 Suppl 2**: S65-9.

36 Bergan T, Thorsteinsson SB, Albini E. Pharmacokinetic profile of fosfomycin trometamol. Chemotherapy 1993; **39**: 297–301.

37 Guibert M, Magny JF, Poudenx F, Lebrun L, Dehan M. [Comparative pharmacokinetics of fosfomycin in the neonate: 2 modes of administration]. Pathol Biol (Paris) 1987; **35**: 750–2.

38 Suzuki S, Murayama Y, Sugiyama E, Sekiyama M, Sato H. [Dose estimation for renal-excretion drugs in neonates and infants based on physiological development of renal function]. Yakugaku Zasshi 2009; **129**: 829–42.

39 Traunmuller F, Popovic M, Konz K-H, Vavken P, Leithner A, Joukhadar C. A reappraisal of current dosing strategies for intravenous fosfomycin in children and neonates. Clin Pharmacokinet 2011; **50**: 493–503.

40 Patel S, Balfour J, Bryson H. Fosfomycin tromethamine. A review of its antibacterial activity, pharmacokinetic properties and ther- apeutic efficacy as a single-dose oral treatment for acute uncomplicated lower urinary tract infections. Drugs 1997; **53**: 637–56.

41 Duez J, Mousson C, Siebor E, et al. Fosfomycin and its application in the treatment of multidrug-resistant Enterobacteriaceae infections. Clin Med Rev Ther 2011; **3**: 123–42.

42 Borgia M, Longo A, Lodola E. Relative bioavailability of fosfo- mycin and of trometamol after administration of single dose by oral route of fosfomycin trometamol in fasting conditions and after a meal. Int J Clin Pharmacol Ther Toxicol 1989; **27**: 411–17.

43 WHO | Antimicrobial resistance: a priority for global health action. Bull World Heal Organ 2015; **93**: 437–512.

44 Sarnat H, Sarnat M. Neonatal encephalopathy following fetal distress: A clinical and electroencephalographic study. Arch Neurol 1976; **33**: 696–705.

45 Lu C, Liu C, Huang Y, et al. Antimicrobial Susceptibilities of Commonly Encountered Bacterial Isolates to Fosfomycin Determined by Agar Dilution and Disk Diffusion Methods. Antimicrob Agents Chemother 2011; **55**: 4295–4301.

46 Morgan C, Herwitker S, Badhawi I, et al. SCAMP: standardised, concentrated, additional macronutrients, parenteral nutrition in very preterm infants: a phase IV randomised, controlled exploratory study of macronutrient intake, growth and other aspects of neonatal care. BMC Pediatr 2011; **11**.

47 The World Health Organization. Hypoglycaemia of the Newborn: Review of the Literature. 1997 DOI:WHO/CHD/97.1 / WHO/MSM/97.1.

Patient Information Sheets and Informed Consent Forms

A study measuring the levels of an additional antibiotic (fosfomycin) to treat severe infections in newborns (NeoFosfo)

KEMRI-Wellcome Trust, Kilifi, Kenya	James Berkley (Principal Investigator), Phoebe Williams, Christina Obiero, Neema Mturi, Claire Gordon, Johnstone Thitiri, Mwanamvua Boga, Sheila Murunga, Joseph Waichungo
KEMRI CCR & CREATES, Strathmore University, Nairobi	Martin Ongas
St George's Hospital, University of London, UK	Mike Sharland, Julia Anna Bielicki, Karin Kipper
University College, London, UK	Joseph Standing
Medical Research Council Clinical Trials Unit at University College London, UK	Sarah Walker
GARDP, Geneva, Switzerland	Sally Ellis
Study Sponsor	Drugs for Neglected Diseases Initiative

We are speaking with you today to give you information about a research study, answer any questions you may have and ask for your consent for your child's participation by signing this form (or for those who cannot write, to select a witness to help them through the consenting process and give a thumb print). Participation in research is voluntary; you are free to decide if you want your child to take part or not. A copy of the signed/thumb printed consent form will be given to you to keep if you agree to participate in this study.

Your child is being admitted to hospital where you will receive the best treatment available at this hospital. As part of normal standard care, these things are usually done:

There will be an admission blood test to check your baby's kidney and liver function, as well as for an infection in the blood; and usually a test for infection in the spinal fluid using a needle;

For sick babies with presumed infections, antibiotic medications are always given into their bloodstream (via a vein).

What is this research about?

Currently, all sick babies admitted to hospital with a presumed infection are treated with two antibiotics. However, bacteria are increasingly becoming resistant to the antibiotics that are normally used. This may make it harder for babies to recover from their illnesses. In Africa, other drugs that work are expensive, and may themselves cause the bacteria to become resistant. New alternatives are therefore needed. One drug that might be able to be used as an alternative is 'fosfomycin'. This drug is already used in Europe and America to treat infections in adults and children and is cheaply available, however we need more information to help us understand how fosfomycin should be used in babies. To help find this out, we want to add fosfomycin to the usual antibiotics we give to provide us with information about how much fosfomycin is in a baby's blood after a dose is given. This will help us know what doses are effective for babies with infection. The people conducting this research have been carefully trained on the research and on ensuring the rights and safety of participants.

Who is carrying out this study?

The Kenya Medical Research Institute is part of the Ministry of Health that carries out research with the aim of finding better ways of preventing and treating illness in the future, for everybody's benefit. One health problem we are trying to discover more about is how best to treat newborn babies with serious infections.

What will it involve for me/my child?

This research will involve 120 children, 60 of whom will receive fosfomycin together with the standard antibiotics and the other 60 will just receive the standard antibiotics which are normally given. We will put your baby into a group by chance, like tossing a coin Your baby will have an equal chance of being in either group.

The doctors and nurses will open a closed envelope which tells them which group your baby will be in. They cannot themselves choose which group your baby will be in.

If your child is assigned to the group receiving fosfomycin, we are asking:

To give your child the standard treatment (two antibiotics), plus fosfomycin as an extra (third) antibiotic for up to one week;

To take a small amount of blood, about half a teaspoon (2.5ml in **total**) for this research at five different times while your baby is in hospital (0.5ml, or a few drops, each time). We will use these samples to check the amount of antibiotics present in your baby's blood and conduct routine tests to check their blood cells, kidney and liver function;

To take a swab from your baby's nappy when they are admitted and discharged from hospital, to check if there are changes to the type of bacteria in their stool;

If your treating Doctor decides that your baby needs a lumbar puncture to check for an infection in the fluid surrounding the brain, we will send any leftover fluid from this test to a lab in England to check the levels of antibiotic present;

To come for a follow-up, visit after 28 days to check if your baby is well. We will pay your transport fare and compensate you for your time (KSh300) for this visit.

Everything else that is done during your stay in hospital will be part of normal tests and treatment requested by doctors. If your baby becomes ill or you are concerned about their health before this follow-up appointment, you may call 0740 310 773 or bring your baby to the ward/clinic for review (and any necessary treatment) by a clinician.

Are there any risks from my child's participation?

KEMRI's priority for every patient is his/her care. The drug being studied is already licensed and in use by adults and children in Europe and other parts of the world, but not yet in Kenya. Your child will be closely observed for any side effects. Occasionally reported side effects include a mild rash or stomach upset, while rare side effects include an allergic reaction or changes to the level of salts in the blood. These are unlikely to occur and may also occur with other antibiotics. Your baby will be closely monitored and treated for any side effects they may develop while participating in the study.

The blood samples for measuring the level of the drug will be taken using a small plastic tube that is like the one that the drugs are given through. If your child needs further blood tests as part of their care, these can be done at the same time. Taking blood can cause a small amount of pain, bruising, swelling, discomfort or a very small chance of infection. We will use a careful procedure to help prevent these from occurring. The use of a cotton swab to collect a sample from your child's nappy poses no risk to your child.

Are there any benefits for me/my child's participation into this study?

Your child will be reviewed daily by one of our study clinicians, together with the regular hospital staff. We will provide alternative antibiotics at no cost if they are needed. We will also provide an extra clinical review 3 weeks after your baby has been discharged from hospital, and you are welcome to contact us at any point or bring your baby to see us at the ward prior to this if you have any concerns in regard to your baby. There is no other direct benefit to your baby in participating. The research aims to benefit society by helping to improve care for children in the future.

What will happen if I do not agree to participate?

If you do not want your baby to take part, no blood samples for research will be taken; but the Doctor may still wish to test your child's blood for regular clinical care. If you agree to participate now, you can still change your mind at any time and withdraw your child from the study. This will not affect your child's care now or in the future.

What will happen to the samples?

All the information and samples collected will be held confidentially. Individual names are removed from all samples and replaced by codes, so that samples can only be linked to the children by people closely concerned with the research. Some of the research tests that will be done on the blood will be done in Kilifi. However, for

some tests that cannot be done in Kenya, part of the sample will be sent overseas (to the United Kingdom) to identify the levels of antibiotics in your baby's blood.

Who will be able to access my child's information in this study?

All information on participants collected in this study will be stored in a confidential manner in locked, secured cabinets and password-protected computers, and will only be accessible to authorized study personnel. Data will be stored to the end of the study, and clinical monitors and regulatory authorities (such as the Kenyan Pharmacy and Poisons Board or Ethics Committee) may check this information to ensure the study is being conducted correctly. In the future, information collected or generated during this study may be used to support new research by other researchers in Kenya and other countries on questions about child health. In all cases, we will only share information with other researchers in ways that do not reveal individual participants' identities. Any future research using information from this study must first be approved by a local or national expert committee to make sure that the interests of participants and their communities are protected. Samples will be stored for up to 5 years.

Who has authorized this research to take place?

All research at KEMRI has to be approved before it begins by an independent ethical review board, the Scientific and Ethical Review Unit in Nairobi and the Oxford University Tropical Research Ethics Committee in the UK, who look carefully at planned work. They must agree that the research is important, relevant to Kenya and follows nationally and internationally agreed research guidelines. This includes ensuring that all participants' safety and rights are respected.

What if I have any questions?

You may ask any of our staff questions at any time. You can also contact the research team using the contacts below:

0740 210 773: KEMRI - STUDY-SPECIFIC MOBILE LINE (LOCAL LANGUAGE SPEAKER)

If you want to ask someone independent about this research, please contact:

Community Liaison Manager, KEMRI Wellcome Trust Research Programme, P.O. Box 230, Kilifi. Telephone: 041 7522 063, Mobile 0723 342 780 or 0705 154 386

Or

The Head, KEMRI Scientific and Ethics Review Unit, P. O. Box 54840-00200, Nairobi;

Telephone numbers: 0717 719477; 0776 399979 Email address: seru@kemri.org

[ENGLISH] A study measuring the levels of an additional antibiotic (fosfomycin) to treat severe infections in newborns (NeoFosfo)

I, being the parent/guardian of, ________________________________ (name), have had the research explained to me. I have understood all that has been read and had my questions answered satisfactorily.

Please place a tick in each box below:

- ☐ I agree / do not agree (delete as appropriate) for my child to take part in this research
- ☐ I agree / do not agree (delete as appropriate) to samples being stored for future research
- ☐ I agree / do not agree (delete as appropriate) to samples being exported to measure blood drug levels

I understand that I can change my mind at any stage, and it will not affect me or my baby in any way.

Parent/guardian's signature: ___________________________ **Date** _____________

Parent/guardian's name: _____________________________**Time** ____________

(Please print name)

I certify that I have followed the study SOP to obtain consent from the [participant]. She/he apparently understood the nature and the purpose of the study and consents to participation in the study. She has been given opportunity to ask questions which have been answered satisfactorily.

Designee/investigator's signature: _______________________ **Date** ____________

Designee/investigator's name: ________________________ **Time** ____________

(Please print name)

Only necessary if the participant cannot read:

I *attest that the information concerning this research was accurately explained to and apparently understood by the subject and that informed consent was freely given by the participant.

Witness' signature: ______________________________ **Date** _____________

Witness' name: _______________________________ **Time** _____________

(Please print name)

***A witness is a person who is independent from the trial or a member of staff who was not involved in gaining the consent.**

Thumbprint of the subject as named above if they cannot write:

THE PARTICIPANT SHOULD NOW BE GIVEN A SIGNED COPY TO KEEP

[SWAHILI]: Utafiti wa kupima viwango vya dawa nyongeza dhidi ya bakteria (fosfomycin) kwa kutibu maambukizi makali kwa watoto wachanga (NeoFosfo)

**Taasisi**	**Watafiti**
KEMRI-Wellcome Trust, Kilifi, Kenya	James Berkley (Mtafiti mkuu),<br>Phoebe Williams, Neema Mturi, Claire Gordon, Johnstone Thitiri, Mwanamvua Boga, Christina Obiero, Sheila Murunga, Joseph Waichungo
KEMRI CCR & CREATES, Strathmore University, Nairobi	Martin Ongas
St George's Hospital, University of London, UK	Mike Sharland, Julia Anna Bielicki, Karin Kipper
University College, London, UK	Joseph Standing
Medical Research Council Clinical Trials Unit at University College London, UK	Sarah Walker
GARDP, Geneva, Switzerland	Sally Ellis
Mfadhili wa utafiti	Drugs for Neglected Diseases Initiative

Tunazungumza na wewe kutoa habari kuhusu utafiti, kujibu maswali yako na tuombe ruhusa yako ya kushirikisha mtoto wako kwa kusaini fomu hii, au kwa wale ambao hawawezi kuandika, kupata shahidi atakaye wasaidia kupitia utaratibu wa kuomba idhini na kuweka alama ya dole gumba. Kushiriki katika utafiti ni hiari; uko huru kuamua kama unataka mtoto wako kushiriki katika utafiti au la. Utapewa nakala ya fomu ya idhini yenye sahihi/alama ya dole gumba uiweke ikiwa unakubali kushiriki katika utafiti huu.

Mtoto wako amelazwa hospitali ambako utapata matibabu bora yanayopatikana katika hospitali hii. Mambo haya yanafanywa kwa kawaida kama sehemu ya huduma ya kawaida:

Kutakuwa na sampuli ya damu wakati wa kulazwa kuangalia jinsi figo na ini zinavyofanya kazi na kwa maambukizi katika damu na kwa kawaida uchunguzi wa maambukizi kwenye maji ya uti wa mgongo inayofanyika kwa kutumia sindano.

Kwa watoto wagonjwa wanaokisiwa kuwa na maambukizi, dawa dhidi ya bakteria huingizwa kwenye damu yao (kupitia mishipa).

Je utafiti huu unahusu nini?

Kwa sasa watoto wote wachanga wanaolazwa hospitali wakidhaniwa kuwa na maambuziki hutibiwa kwa dawa dhidi ya bakteria aina mbili. Hata hivyo, bakteria wanazidi kuwa sugukwa dawa zinazotumika kwa kawaida. Hii inaweza kufanya iwe vigumu kwa watoto wachanga kupona kutoka kwa magonjwa. Hapa Afrika, dawa mbadala zinazofanya kazi ni ghali na pia zinaweza kufanya bakteria kuwa sugu. Kwa hivyo, dawa mpya mbadala zinahitajika. Dawa moja inayoweza kutumika kama mbadala ni 'fosfomycin'. Dawa hii inatumika bara Uropa na Amerika kutibu maambukizi katika watu wazima na watoto na iko rahisi kupatikana,Hata hivyo tunahitaji habari zaidi ili kutusaidia kuelewa jinsi 'fosfomycin' inastahili kutumika kwa watoto wachanga. Ili kutusaidia kujua hili, tungependa kujumuisha fosfomycin kwa dawa za kawaida dhidi ya bakteria tunazopeana ili kutupatia habari kuhusu kiwango cha fosfomycin kilicho kwenye damu ya mtoto mchanga baada ya kupewa kiwango chadawa (dozi?). Hii itatusaidia kujua ikiwa viwango ni vya sawa kwa watoto wachanga walio na maambukizi.

Ni nani anayefanya utafiti huu?

Shirika la utafiti la KEMRI Wellcome Trust ni sehemu ya wizara ya afya linalofanya utafiti kwa lengo la kutafuta njia bora za kukinga na kutibu magonjwa kwa siku za usoni kwa manufaa ya watu wote. Tatizo moja la kiafya ambalo tunajaribu kujifunza zaidi ni jinsi ya kutibu vyema watoto wachanga wenye maambukizi makali. Watu wanaofanya utafiti huu wamepata mafunzo kwa uangalifu na wanahakikisha haki na usalama wa wanaoshiriki vinaheshimiwa.

Utafiti huu utahusisha watoto 120, 60 ambao watapewa fosfomycin pamoja na dawa za kawaida na wengine 60 wataendelea tu kupewa dawa za kawaida ambazo hupewa kwa kawaida. Mtoto wako yuko na nafasi sawa ya kuwa kwenye moja wapo ya makundi haya. Tutamuweka mtoto wako kwenye kungi kwa njia ya bahati na sibu, kama vile kurusha sarafu.. Madaktari na wauguzi watafungua bahasha zilizofungwa ambaazo zinaonyesha mtoto wako atakuwa kundi gani. Hawana njia ya kuchagua kundi ambalo mtoto wako ataingia.

Je, itahusisha nini kwangu/mtoto wangu?

Ikiwa mtoto wako atawekwa kwa kundi la wanaopewa fosfomycin, tunauliza:

Kumpa mtoto wako matibabu ya kawaida (dawa 2 dhidi ya bakteria – antibiotiki?); na kuongeza fosfomycin, kama dawa ya ziada (ya tatu) kwa wiki moja.

Kuchukua kiwango kidogo cha damu, kama nusu ya kijiko cha chai (mililita 2.5 **jumla**) kwa utafiti huu mara tano wakati tofauti mtoto wako akiwa hapa hospitalini (mililita 0.5, au matone machache, kila wakati). Tutatumia sampuli hizi kuangalia kiwango cha dwa dhidi ya bacteria kilichoko kwenye damu ya mtoto wako na kufanya vipimo vya kawaida kuangalia kuangalia chembechembe za damu, figo na ini vinavyofanyakazi.

Kuchukua sampuli ya choo kutoka kwa mtoto wako atakapokuwa amelazwa na watakapotolewa hospitali, kuangaliaa iwapo kuna mabadiliko ya aina ya bakteria katika choo chao.

Iwapo daktari wako anayetibu mtoto ataamua ya kwamba mtoto wako anahitaji kutolewa maji ya mti wa mgongo kuangalia maambukizi ya maji yanayozunguka ubongo tutatuma mabaki ya maji kutoka kwa utafiti huu kwa mahabara iliyoko Uingereza ili kuangalia kiwango cha dawa dhidi ya bakteria kilichoko.

Uje kwa ziara za ufuatilizi baada ya siku 28 ili kuangalia kama mtoto wako yuko salama Tutalipia usafiri wako na kufidia muda wako (Shilingi 300) kwa kila ziara.

Kila kitu kingine ambacho kitafanywa wakati uko hospitali itakuwa sehemu ya vipimo vya kawaida na matibabu vinavyo ulizwa na madaktari.

Ikiwa mtoto wako atakupwa mgonjwa, au una wasi wasi kuhusu afya yake kabla ya tarehe ufuatilizi wa mwezi wa kwanza, unaweza kupiga simu **0740310773** timu watafiti iliyopeanwa hapa chini au umlete mtoto wako kwa wodi/kliniki kwa ukaguzi (na matibabu yoyote yanayohitaji) daktari.

Je, kuna madhara yoyote kwa mtoto wangu kushiriki?

Kipao mbele cha KEMRI kwa kila mgonjwa ni uangalizi wake. Dawa inayotafitiwa tayari imeidhinishwa kutumika katika watu wazima na watoto huko bara Uropa na semehu nyengine za ulimwengu, lakini bado hapa Kenya. Mtoto wako atafuatilizwa kwa ukaribu kwa madhara yoyote kutokana na dawa hii. Mara chache, madhara ambayo yameripotiwa yanajumuisha vipele na kuumwa na tumbo madhara nadra ni kama mabadiliko ya kiwango cha chumvi kwenye damu. Haya yanauwezekano mchache kutokea, na hata yanafanyika hata kwa dawa nyengine dhidi ya bakteria. Mtoto wako atafuatiliwa kwa ukaribu na atibiwe madhara yoyote yahayoweza kutokea wakati akishiriki kwenye utafiti

Sampuli ya damu ya vipimo vya kiwango cha dawa zitachukuliwa kutumia mrija mdogo wa plastiki ambayo ni kama zile zitumikazo kupitisha dawa. Ikiwa mtoto wako anahitaji vipimo zaidi vya damu kama sehemu ya huduma yake, hivi vitafanywa wakati mmoja. Kuchukua damu kutoka mkononi kunaweza kusababisha maumivu machache, mkwaruzo, kufura au uwezekano mdogo wa kupata maambukizi. Tutatumia utaratibu wa makini ili kuzuia haya. Utumizi wa kifaa cha pamba kuchukua sampuli ya choo kutoka kwa kibinda cha mtoto wako hakuna hatari yoyote kwa mtoto wako.

Je, kuna manufaa kwangu/mtoto wangu kwa kushiriki katika utafiti huu?

Mtoto wano atachunguzwa kila siku na mmoja wa daktari wa utafiti, pamoja na wafanyikani wa kawaida wa hospitali. Tutatoa dawa mbadala dhidi ya bakteria bila kuhitaji malipo zaidi. Pia tutapeana ukaguzi wa kimatibabu wiki tatu baada ya mtoto wako kuruhusiwa kuenda nyumbani, na unaweza kututafuta wakati wowote, au kumleta mtoto wako wodini kabla haya ikiwa una wasi wasi wowote kuhusu mtoto wako. Hakuna

manufaa mengine ya moja kwa moja kwa kushiriki. Utafiti huu unalenga kunufaisha jamii kwa kuboresha huduma kwa watoto siku za usoni.

Je, kutafanyika nini nikikataa kushiriki?

Ikiwa hutaki mtoto wako ashiriki, hakuna sampuli za damu zitachukuliwa kwa utafiti, hata hivyo madaktari bado watahitaji kupima damu ya mtoto wako kama ilivyo kawaida kwa huduma. Ukikubali kushiriki sasa, unaweza kubadilisha mawazo yako wakati wowote na kumuondoa mtoto wako kutoka kwa utafiti na hakuna sampuli ya damu au habari za utafiti zaidi ambazo zitachukuliwa. Hii haitaathiri huduma kwa mtoto wako sasa wala siku za usoni.

Je, nini kitafanyika kwa sampuli?

Habari zote na sampuli zitakazokusanywa zitawekwa katika hali ambayo haiwafikii wengine. Majina ya watu binafsi yanatolewa kutoka kwa sampuli zote na yabadilishwe na nambari maalum (codes), kuhakikisha kwamba sampuli zinaweza tu kuambatanishwa na watu wanaohusika na utafiti kwa ukaribu. Baadhi ya vipimo vitakavyofanywa kwenye sampuli ya damu vitafanywa hapa Kilifi. Hata hivyo kwa baadhi ya vipimo ambavyo haviwezi kufanywa Kenya, baadhi ya sampuli zitatumwa ng’ambo nchi ya Uingereza ili kutambua viwango vya dawa dhidi ya bakteria vilivyo katika damu ya mtoto wako. Sampuli zinaweza kuhifadhiwa kwa hadi miaka mitano.

Ni nani atakayefikia habari kunihusu mimi/mtoto wangu katika utafiti huu?

Habari zote kuhusu washiriki katika utafiti huu zitahifadhiwa katika hali ambayo haziwezi kufikia wengine, katika kabati zilizofungwa na komputa zinazohitaji neno siri kufunguliwa zinazofikiwa tu na wafanyikazi walioidhinishwa. Habari zitahifadhiwa hadi mwisho wa utafiti na uchambuzi; na wakaguzi wa tafiti za kimatibabu au wakaguzi wengine na mamlaka za udhibiti (kama vile Bodi ya Kenya ya Dawa na Sumu au Kamati ya Maadili) wanaweza kukagua habari ili kuhakikisha utafiti unafanywa sawa. Ripoti yoyote au jarida kuhusu utafiti huu hazitatumia majina au vitambulishi vya mshiriki.

Siku za usoni, habari zitakazokusanya wakati wa utafiti huu zitatumika kusaidia tafiti mpya zitakazofanywa na watafiti wengine nchini Kenya na nchi zengine kuhusu maswali ya afya ya mtoto. Katika hali zote, tutasambaza tu habari katika njia ambayo haidhihirishi vitambulisho binafsi vya washiriki. Kwa mfano, tutaondoa habari ambazo zinaweza kutambulisha watu, kama vile majina yao na kule wanakoishi, na tubadilishe na nambari maalum (codes). Kisha, tafiti zozote za siku zijazo zitakazotumia habari kutoka kwa utafiti huu lazima kwanza ziidhinishwe na kamati za wataalam za Kilifi na za kitaifa ili kuhakikisha kwamba maslahi ya washirikina jamii zao yanalindwa.

Ni nani ameidhinisha utafiti huu?

Tafiti zote za KEMRI ni lazima ziidhinishwe kabla kuanza na kamati iliyo huru ya KEMRI ya kukagua maadili, Kitengo cha Sayansi na Maadili huko Nairobi na Kamati ya Chuo Kikuu cha Oxford ya Maadili ya utafiti huko Uiengereza, zinazoangalia kwa makini mpangilio wa kazi. Ni lazima wakubali kwamba utafiti ni muhimu, wafaa Kenya na unafuata taratibu zinazokubalika kitaifa na kimataifa. Hii ni pamoja na kuhakikisha kwamba usalama na haki za washiriki zinaheshimika.

Je nikiwa na maswali yoyote?

Uko huru kumuuliza maswali mfanyaji kazi wetu yeyote wakati wowote. Pia unaweza kuwasiliana na kundi la utafiti kutumia anwani zifuatazo:

0715 938 077 KEMRI – Nambari ya mradi wa utafiti (Mtu anayezungumza lugha ya hapa)

Ukitaka kumuuliza mtu huru kuhusu utafiti huu tafadhali wasiliana na:

Meneja wa kitengo cha uhusiano mwema na jamii, Shirika la utafiti la KEMRI Wellcome Trust, S. L. Posta 230, Kilifi. Simu: 0723 342 780 au 041 7522 063

Na

Kiongozi, Kitengo cha kukagua sayansi na maadili cha KEMRI, S. L. Posta 54840-00200, Nairobi; Nambari ya simu: 0717 719477; 0776 399979 Barua pepe: seru@kemri.org

Utafiti wa kupima viwango vya nyongeza vya dawa dhidi ya bakteria (fosfomycin) kutibu maambukizi makali katika watoto wachanga (NeoFosfo)

Mimi, nikiwa mzazi/mlezi wa ______________________________(jina la mtoto),] nimeelezewa utafiti huu. Nimeelewa yote yaliyosomwa/elezwa na maswali yangu yamejibiwa kikamilifu.

- ☐ Ndio nakubali kumruhusu mtoto wangu kushiriki kwenye utafiti huu
- ☐ Ndio nakubali sampuli zihifadhiwe na zitumike kwa utafiti wa siku za usoni
- ☐ Ndio nakubali sampuli zisafirishwe ng'ambo kupima viwango vya dawa katika damu

Naelewa kwamba naweza kubadilisha nia wakati wowote na haitaniathiri mimi/mtoto wangu kwa njia yoyote.

Sahihi ya mhusika/ mzazi/mlezi:___________________________Tarehe:_________________

Jina la mhusika/ mzazi/mlezi:______________________________Saa:___________________

(tafadhali andika jina kwa herufi kubwa)

Ninathibitisha kwamba nimefuata muongozo wa utafiti wa kuchukua idhini kutoka kwa mshiriki. Ni wazi kuwa ameelewa asili na madhumuni ya utafiti na amekubali kushiriki katika utafiti. Amepewa nafasi ya kuuliza maswali ambayo yamejibiwa kikamilifu.

Sahihi ya mwakilishi/ mtafiti: ___________________________ Tarehe ______________

Jina la mwakilishi/mtafiti: ________________________________ **Saa** ____________

(Tafadhali andika jina kwa herufi kubwa)

Ni muhimu tu kama mshiriki hawezi kusoma

Ninathibitisha kwamba habari kuhusu utafiti huu zimeelezwa kikamilifu na ni dhahiri kuwa mshiriki ameelewa na kwamba idhini imetolewa kwa hiari na mshiriki.

Sahihi ya shahidi: _________________________________ Tarehe _____________

Jinan a shahidi: ___________________________________ Saa ________________

(Tafadhali andika jina kwa herufi kuubwa)

*Shahidi ni mtu ambaye yuko huru kutokana na utafiti au mfanyajikazi ambaye hakuhusika katika kupata idhini.

Alama ya kidole gumba cha mzazi kama alivyotajwa hapo juu ikiwa hawezi kuandika:

MSHIRIKI/MZAZI/MLEZI SASA APEWE NAKALA ILIYOWEKWA SAHIHI AHIFADHI

[GIRIAMA]: Utafiti wa kupima viwango zha dawa ya nyongeza dhidi ya bakteria (fosfomycin) kahi za kutibu maambukizi makali kahi za ahoho atsanga (NeoFosfo)

**Taasisi**	**Atafiti**
KEMRI-Wellcome Trust, Kilifi, Kenya	James Berkley (Mutafiti mubomu),<br>Phoebe Williams, Neema Mturi, Claire Gordon, Johnstone Thitiri, Mwanamvua Boga, Christina Obiero, Sheila Murunga, Joseph Waichungo
KEMRI CCR & CREATES, Strathmore University, Nairobi	Martin Ongas
St George's Hospital, University of London, UK	Mike Sharland, Julia Anna Bielicki, Karin Kipper
University College, London, UK	Joseph Standing
Medical Research Council Clinical Trials Unit at University College London, UK	Sarah Walker
GARDP, Geneva, Switzerland	Sally Ellis
Mufadhili wa utafiti	Drugs for Neglected Diseases Initiative

Funazungumuza na uwe kumboza habari kuhusu utafiti, kujibu maswali gakwako na fovoye ruhusayo kwa ushiriki wa mwanao na kusaini fomu ii, hedu kwa aryahu ambao kamadima kwandhika, kupata shahidhi andiye asaidhia kukirira utharathibu wa kuvoya idhini na kungiza alama ya dzalagumbe. Kushiriki kahi za utafiti ni hiari; uhuru kuamua kala undahenza mwanao kushiriki kahi za utafiti uu ama la. Undagerwa fomu ya idhini yenye saini /alama ya dzalagumbe iriyochapishwa undapewa ili uenderere kala unakubali kushiriki kahi za utafiti uu.

Mwanao alazwa kahi za sipitali ambaho andahokera matibabu madzo gapatikanago kahi za sipitali ii. Here sehemu ya huduma ya kawaida mambo gathuwago nikukala ganahendwa:

Fundahala sampuli ya mulatso wakati wa kulazwa kolola jinsi figo na ini zihendazho kazi na kwa maambukizi kwa damu na kwa kawaida uchunguzi wa maambukizi kwenye madzi ga utafiti wa utu wa mongo ihendekayo kuhumira shindano.

Kwa ahoho akongo ambao manakisiwa kukala na maambukizi, dawa dhidi ya bakteria nikungizwa kahi za mulatso wa kwao kukirira mishipani.

Utafiti uno unahusu noni?

Ana atsanga osini Mario malazwa sipitali na manakisiwa kukala mana maambukizi nikukala manatibiwa na dawa mbiri dhidi ya kidudu cha bakteria ilazhwayo kukirira mishipani. Hata vizho, vidudu zha bakteria vinaenderera kukala na sugu ya dawa dhidi yao ambazo zinahumika kwa kawaida. Ii inadima kureha ugumu wa ahoho kuhola kula kwa makongo gao. Kahi za Afrika, dawa mbadala ambazo zinadima kuhenda kazi zi gali na pia zinadima kusababisha vidudu zha bakteria kukala sugu. Kwa vizho, dawa mbisha mbadala inahitajika. Dawa ambayo inadima kuhumika here dawa mbadala ni 'fosfomycin'. Dawa ii inahumika kuko Ulaya na Marekani kutibu maambukizi kwa atu azima na ahoho na inapatikana kwa bei rahisi lakini kaidzaanza kuhumika Kenya. Funahitaji habari zaidi ili zifusaidie kuelewa kuhusu mahumizi ga fosfomycin kahi za ahoho a Kenya. Ili kumanya vivi, funahenza fungehenza kungeza fosfomycin kahi za dawa za kawaida dhidi ya bakteria ili fupate habari kuhusu kiwango cha 'fosfomycin' kahi za mulatso wa mwana mutsanga baada ya kiwango maalumu cha dawa kulazhwa. Iii indafusaidia kumanya kala kiwango kicho ni sawa kwa ana atsanga enye maambukizi.

Ni hani ahendaye utafiti uno?

Shirika ra utafiti ra afya ra Kenya ni shirika kahi za wizara ya afya rihendaro utafiti kwa lengo ra kutafuta ngira mbidzo za kuchinga na kutibu makongo siku za usoni kwa manufaa ga kila mumwenga. Tatizo mwenga raho ra kiafya ambaro funajeza kudzifundisha zaidi ni jinsi ya kutibu ahoho atsanga enye maambukizi makali.

Utafiti uu undahusisha ahoho 120, 60 mandahokera fosfomycin hamwenga na dawa ya kawaida na o angine 60 mandahokera yo dawa ya kawaida hakeye ambayo kwa kawaida nikukala inalazhwa. Fundahumira bahasha zidzizofungwa ili fumuike mwanao kwa kundi mwenga kahi za go makundi mairi ga utafiti kwa ngira mabayo inalazha nafasi sawa ya kukala kahi za kundi rorosi. II inamaanisha fundamwika mwanao kahi za kundi kwa ngira ilazhayo nafasi ya sawa ya kuikwa kahi za kundi mwenga, here muchezo wa kuzungulusha shilingi. Mwanao andakala na nafasi sawa ya kukala kahi za kundi rorosi. Madaktari na auguzi kamana ngira ya kumutsagula mwanao kukala kahi za kundi rorosi.

Indahusisha noni kwangu/kwa mwanangu?

Kala mwanao adzaikwa kahi za kundi rihokeraro fosfomycin, funauza:

Kumupa mwanao matibabu ga kawaida (dawa mbiri dhidi ya bakteria), nay a hahu ya nyongeza fosfomycin kwa wiki mwenga kwa jumula;

Kuhala kiwango kithithe cha mulatso, here nisu kujiko cha chai (mililita 2.5 **kwa jumula**) kwa ajili ya utafiti uu kwa mara 5 wakati mwanao akikala sipitali (mililita 5, hedu madeswe machache, kila wakati). Fundahumira sampuli zizi kulola kiwango cha dawa dhidi ya bakteria kahi za mulatso wa mwanao na kuhenda vipimo zha chembechembe za mulatso wa kwao na viryahu figo na maini vihendazhokazi.

Kuhala sampuli kukirira kuhangusa kibinda cha mwanao wakati kala adzalazwa sipital na kumbozwa sipitali, kulola kala kuna mabadiliko kahi za aina ya bakteria kahi za choo cha kwakwe;

Kala daktari akutibuye andaamua kukala mwanao anahitaji kumbozwa sampuli ya madzimadzi ga mongoni ili kulola maambukizi kwa go madzimadzi kazungukago wongo, fundahuma madzimadzi gaga gandigokala gadzasala kula kwa kipimo kiki kwa maabara ga ng’ambo ili kulola kiwango cha dawa dhidi ya bakteria;

Kudza kwa ziara ya thuwirizi baada ya siku 28 ili kulola kala mwanao kana utu, hedu kala wakala na wasiwasi wowose ho mbereni. Fundariha tikiti ya kwako na fidia ya muda wa kwako (shilingi 300) kwa ziara ii.

Mambo mangine gosi gandigohendwa wakati undokala sipitali gandakala sehemy ya vipimo na matibabu ga kawaida gamalwago ni dakitari.

Mwanao akikala mukongo hedu kala una wasiwasi kuhusu afya ya kwakwe kabla ya siku iriyopangwa ya uthuwirizi ya mwezi muwenga, undapiga simu kwa namba **0740310773** hedu umurehe mwanao wodini/kliniki kwa ukaguzi wa daktari.

Kuna madhara gogosi kwa kushiriki kwa mwanangu?

Jambo ra muhimu kwehu kwa kila mushiriki ni huduma ya kwakwe. Dawa huichunguzayo yaidhinishwa kare na inahumika kwa atu azima na ahoho ko Ulaya na sehemu nyingine za dunia, lakini si Kenya. Mwanao andathuwirizwa kwa uhehi ili kulola madhara gogosi ga dawa. Mara kwa mara, madhara ambago gaonewa na kuripotiwa ni harara na kubujika tumbo (na madhara ambago ni nadra sana mabadiliko kahi za kiwango cha munyu kahi za mulatso) ela gaga si rahisi koneka na pia ganadima gakaoneka na dawa dhidi ya bakateria nyingine. Mwanao andathuwirizwa kwa uhehi na agerwe matibabu ga madhara gogosi gadimago kumbola wakati akishiriki kahi za utafiti uu.

Sampuli za mulatso za kupima kiwango cha dawa zindahalwa kwa kuhumira muridza muthithe wa plastiki ambao ni here uryahu ambao dawa zinangizirwa. Kala mwanao andahitaji vipimo zha mulatso zaidi here sehemu ya huduma ya kwakwe, vivi vinadima vikahendwa wakati mumwenga. Kumboza damu kunadima kukasababisha maumivu vichache, kuvarurwa, kubujika na uwezekano muthithe wa maambukizi. Fundahumira utaratibu kwa kumakinika ili kusaidhia kuchinga mambo gaga. Mahumizi ga kapamba wakati wa kuhala sampuli kula kwa kibinda cha mwanao kakusababisha madhara gogosi kwa mwanao.

Kuna manufaa gogosi kwangu/kwa mwanangu kwa kushiriki kahi za utafiti uno?

Mwanao andakaguliwa kila siku ni mumwenga wa daktari wehu wa utafiti, hamwenga na muhenzi wa kazi wa sipitali. Fundalazha dawa mbadala dhidi ya vidudu zha bakteria bila mariho kala zindahitajika. Pia fundalazha ukaguzi wa ziada wa kimatibabu wiki 3 baada ya mwanao kumbozwa sipitali, na unaruhusiwa kuwasiliana nasi

wakati wowosi hedu umurehe mwanao ili humuhudumie kuku wodini kabla ya siku ii kala una wasiwasi wowosi kuhusu mwanao. Kakuna manufaa mangine ga mwenge kwa mwenge kwako kwa kushiriki, lakini undafusaidhia kuboresha huduma kwa ahoho siku zidzazo.

Kundakalani nikikahala kushiriki?

Kushiriki kahi za utafiti ni hiari. Uhuru kuamua kala unamala kushiriki kahi za utafiti uu hedu kwenzi. Kala kwenzi mwanao ashiriki, sampuli za damu za utafiti kazindahalwa, idzaho daktarin anadma akamala kumupima mwanao mulatso here kawaida kwa huduma ya kwao. Ukikubali kushiriki vikara, unadima ukagaluza maazogo wakati wowosi na umumboze mwanao kula kwa utafiti na kakuna sampuli ya damu ya zaidi hedu habari za utafiti zindizohalwa. Iii kaindaathiri huduma ya afya ya mwanao vikara na siku zidzazo**.**

Sampuli zindahendwadze?

Habari zosi na sampuli zindizokusanywa zindaikwa kwa hali ambayo atu anhine kamadnadima kuzifikirira. Madzina gam utu binafsi ganambozwa kula kwa sampuli na gabadilishwe na nambari maalumu, ili sampuli zidime kuambatanishwa na ahoho ni atu ambao manahusika na utafiti kwa uheho hakeye. Baadhi ya vipimo zha utafiti ambazho vindahendwa kahi za mulatso vindahendwa Kilifi. Hat vizho, kwa vipimo ambazho kavidima kuhendwa Kenya, sehemu ya sampuli indahumwa ng'ambo kuko Ulaya ili kutambua kiwango cha dawa dhidi ya bakteria kahi za mulatso wa mwanao. Sampuli zinadima kuikwa hadi miaka mitsano.

Ni hani andedima kufikira habari za mwanangu kahi za utafiti uno?

Habari zosi za ashiriki zindizokusanywa kahi za utafiti uu zindahifadhiwa kwa hali ambayo kazindaafikira atu angine kahi za kabati zidzizofungwa na komputa zihitajizo namba maalumu kuvugulwa na zindafikirwa ni atu madzioidhinishwa kahi za utafiti. Habari zindahifadhiwa muhadi utafiti na uchambuzi ugome; na akaguzi a kimatibabu na enye mamlaka ya kisheria (here Bodi ya madawa na sumu ya Kenya [Kenyan Pharmacy and Poisons Board] hedu kamati za maadili) manadima makalola habari zizi ili kuhakikisha utafiti unahendwa sawasawa. Ripoti yoyosi hedu uchapishaji kuhusu utafiti uu kaundahumira dzina ra mushiriki hetu vitambulishi vya kwakwe.

Siku za usoni, habari zindizokusanywa hedu zimbolanazo na utafiti uu zinadima zikahumika kusaidhia tafiti mbisha za atafiti angine haha Kenya na tsi nyingine kuhusu maswali kuhusu afya ya muhoho. Kahi za hali zosi, fundalazha habari kwa atafiti angine kwa angira ambazo kazidima kugunula vitambulishi binafsi zha ashiriki. Kwa mufano, fundausa habari ambazo zinadima kumanyisa atu, here madzina gao na maishiko na fubadilishe habari zizi na namba maalumu. Tafiti zosi za siku zidzazo zihumirazo habari kula kwa utafiti uu ni lazima kwanza ziidhinishwe ni kamati za kitaalamu za haha Kilifi na ya kitaifa ili kuhakikisha kukala maslahi ga ashiriki na jamii zao ganarindwa.

Ni hani adzeruhusu utafiti uno uhendeke?

Tafiti zosi za KEMRI ni sharti ziidhinishwe kabla kuanza ni Bodi huru ya ukaguzi wa maadili, kitengo cha kukagua sayansi na maadili kuko Nairobi na Oxford University Tropical Research Ethics iriyo Uingereza, zilolazo kwa makini mpangilio wa kazi. Ni lazima makubali kukala utafiti ni muhimu, unafaa Kenya na unathuwa mulongozo ukubalikao kitaifa na kimataifa. Iii ni hamwenga na kuhakikisha usalama na haki za ashiriki vinaheshimika.

Je nikikala na swali rorosi?

Unadima ukamuza muhenzi wa kazi wehu yeyosi waswali wakati wowosi. Unadima pia ukawasiliana na kundi ra utafiti kwa kuhumira anuani ithuwayo:

0715 938 077 KEMRI – Nambari ya mradi wa utafiti (Mtu anenaye lugha zha haha)

Ukimala kumuza mutu huru chochosi kuhusu utafiti uu tafadhali wasiliana na:

Meneja wa kitengo cha uhusiano mudzo na jamii, KEMRI Wellcome Trust Research Programme, S. L. Posta 230, Kilifi. Simu: 041 7522 063, Rununu 0723 342 780 hedu 0705 154 386

Hedu

Kiongozi, Kitengo cha kukagua sayansi na maadili cha KEMRI, S. L. Posta 54840-00200, Nairobi; Nambari ya Simu: 0717 719477; 0776 399979 Barua pepe: seru@kemri.org

MSHIRIKI/MZAZI/MLEZI SASA APEWE NAKALA ILIYOWEKWA SAHIHI

Utafiti wa kupima viwango zha dawa ya nyongeza dhidi ya bakteria (fosfomycin) kahi za kutibu maambukizi makali kahi za ahoho atsanga (NeoFosfo)

Mimi nikikala ni muzhazi/murezi wa, ________________________________ (dzina), nidzaelezerwa kuhusu utafiti uu. Nidzaelewa gosi gadzigoshomwa na maswali gangu gajibiwa kikamilifu.

- ☐ **Nakubali mwanangu ashiriki kahi za utafiti uu**
- ☐ **Nakubali sampuli zihifadhiwe kwa tafiti za siku zidzazo**
- ☐ **Nakubali sampuli zisafirishwe ng'ambo ili kupima viwango zha dawa kahi za mulatso**

Ninaelewa kwamba nadima kugaluza maazo ganu wakati wowosi na kaindaniathiri mimi hedu mwanangu kizhozhosi.

Sahihi ya muzhazi/murezi: ___________________________ **Tarehe** _____________

Dzina ra muzhazi/murezi: _____________________________**Saa** ____________

(Tafadhali ndhika dzina kwa herufi bomu)

Ninathibitisha kukala nidzathuwiriza utaratibu wa kuvoya idhini wa utafiti uu ili kuvoya idhini kula kwa mushiriki. Ni wazi kukala adzaelewa hali na lengo ra utafiti na alazha idhini ya kushiriki kahi za utafiti. Adzagerwa nafasi ya kuuza maswali ambago gajibiwa kikamilifu.

Sahihi ya mtafiti/muwakilishi: _______________________ **Tarehe** ____________

Dzina ra mutafiti/muwakilishi: ________________________ **Saa** ____________Tafadhali ndhika dzina kwa herufi bomu)

Ni muhimu tu kala mushiriki kadima kushoma:

*Ninathibitisha kukala habari kuhusu utafiti uu zaelezwa kwa usahihi na ni wazi kukala zaeleweka kwa mushiriki na kukala idhini yalazhwa kwa hiari ni mushiriki.

Sahihi ya shahidi: ______________________________ **Tarehe** _____________

Dzina ra shahidi: _______________________________ **Saa** _____________

(Tafadhali ndhika dzina kwa herufi bomu)

*__Shahidi ni utu ariyehuru kula kwa utafiti hedu muhenzi wa kazi ambaye kahisikire na kuvoya idhini__

Alama ya dzalagumbe la mushiriki here adzohadzwa ho dzulu kala kadima kundhika:

MUSHIRIKI VIKARA NI AGERWE NAKALA IDZIYOIKWA SAHIHI ADZIIKIRE
