# Supplementary material for: Randomised controlled trial of fosfomycin in neonatal sepsis: pharmacokinetics and safety in relation to sodium overload
Source: Arch Dis Child. 2022 Jan 25;107(9):802–10. doi: 10.1136/archdischild-2021-322483 (PMC9411916; doi:10.1136/archdischild-2021-322483)
Supplement: Supplementary file 2 [file archdischild-2021-322483supp002.pdf]

Randomised Controlled Trial of Fosfomycin in Neonatal Sepsis: Safety and Pharmacokinetics

Supplement 2

Additional methods and results

Table of Contents

Supplementary Methods **2**
- Study Site and Population 2
- Screening and Enrolment of Study Participants 2
- Procedures 2
- Study Treatment 2
- Clinical Definitions 3
- Safety Assessment and Follow up 3
- PK Sample Processing and Measurement 3

Supplementary Figures **4**
- Figure S1: Outline of study procedures 4
- Figure S2. Plasma concentration-time curves for A: IV and B: oral fosfomycin administered at 100mg/kg/dose twice daily. 5

Supplementary Tables **6**
- Table S1: Baseline laboratory test parameters by treatment arm 6
- Table S2: Blood and CSF culture results by treatment arm 7
- Table S3: Standard-of-care (SOC) antibiotic changes and day 2 sample collection 8
- Table S4: Serious and non-serious AEs by treatment arm, severity and MedDRA coding classification 9
- Table S5. Anticipated adverse events by treatment arm 13
- Table S6: AE outcome at study termination excluding events that either resolved or occurred in neonates who died 14
- Table S7 Simulated Steady State PK Summary – Sub-populations 15

References **16**

Obiero CW, *et al. Arch Dis Child* 2022; 107:802–810. doi: 10.1136/archdischild-2021-322483

Supplementary Methods

Study Site and Population

Kilifi County Hospital (KCH) is a Level IV government health facility located along the Kenyan coast that serves a mostly rural population including patient referrals from within Kilifi county and surrounding counties. The Kenya Medical Research Institute – Wellcome Trust Research Programme (KWTRP) is located at KCH. The paediatric ward has a dedicated neonatal unit with 3 incubators and 21 cots while the high dependency unit (HDU) has 4 incubators and 12 cots, with capacity to accommodate more neonates within the main HDU ward which has six beds. Mechanical ventilation and central venous lines for monitoring and parenteral feeding are not available at KCH. Treatment of sick neonates at KCH is done according to the Kenya national paediatric guidelines.[1]

Screening and Enrolment of Study Participants

All neonates requiring admission to KCH were screened at presentation to the ward and this was enhanced by the use of an electronic-based data management system (Kilifi Integrated Data Management System). Assessment of eligibility was done by qualified clinicians and included a medical history, clinical examination, and review of admission complete blood count (CBC) and biochemistry results which were done for all admissions as part of routine care. Inclusion and exclusion criteria are outlined in the manuscript and **Supplement 1** (trial protocol). Gestational age was estimated using the Ballard Maturational Assessment.[2] Screening procedures were done in parallel with admission procedures, prioritizing the latter to ensure that treatment of sick neonates was not delayed. Trained study staff undertook the informed consent process with parents or legal guardians of neonates who were found to be eligible for study inclusion and those who provided consent for study participation were transferred to the HDU for continued treatment, randomization to treatment arm and additional study procedures.

Procedures

All neonates had blood samples for CBC, clinical biochemistry, blood slide for malaria parasites, and blood culture taken at admission as part of routine investigations. A lumbar puncture (LP) was performed as indicated according to Kenya national paediatric guidelines[1] in neonates lacking contraindications of an LP.[3]
All trial participants were reviewed daily by trained clinicians until discharge. Concomitant medication administered as a result of ongoing illness or adverse events (AEs) was documented in appropriate case report forms (CRFs). Blood samples for CBC and biochemistry were obtained at baseline (at admission as part of clinical care), 48 hours and 7 days for those still hospitalized. Additional samples were taken if clinically indicated. To minimize the number of times SOC-F participants underwent venepuncture, collection of blood samples for safety assessment was done at the same time as pharmacokinetics (PK) sample collection.
CBC and biochemistry tests were measured using Coulter AcT 5Diff CP (Beckman Coulter, Inc. USA) and ILab Aries (Instrumentation Laboratory, USA) respectively at the KWTRP laboratory, and results were reviewed in real time for patient care. Blood samples for culture were collected and processed in BACTEC Peds Plus/F bottles with a BACTEC 9050 instrument (Becton Dickinson, Oxford, UK). Positive samples were sub-cultured on standard media by routine microbiological techniques as previously described.[4] Cerebrospinal samples were collected, processed and cultured as previously described and organisms identified using standard methods.[5 6]

Study Treatment

SOC antibiotics (ampicillin [50mg/kg/dose twice daily if age ≤7 days or thrice daily if >7 days] plus gentamicin [3mg/kg or 5mg/kg once daily for participants <2kg or ≥2kg respectively] were prescribed according to WHO[7] and Kenya national paediatric guidelines.[1] Second line antibiotics were prescribed according to guidelines, or in response to culture results.
Two formulations of fosfomycin were administered in this study as follows:

1. Fomicyt™ 40 mg/ml solution powder for infusion (Infectopharm GmbH)
   i. Supplied in clear type-II glass bottles with a rubber stopper (bromobutyl rubber) and pull-off cap containing 2g fosfomycin (in 100 ml bottle)
   ii. IV dose was prescribed as 100mg /kg bodyweight, twice daily for 2 days or until the participant was able to take oral fosfomycin.
   iii. The solution was prepared for infusion by dissolving 2g fosfomycin powder in 50ml of 5% or 10% dextrose infusion.
   iv. IV fosfomycin was administered as a slow push.
   v. Each bottle was dispensed to a single participant and used for one dose only.

2. Fosfocina® 250mg/5ml powder for oral suspension (Laboratorios ERN S.A)
    i. The suspension is presented in glass bottles with a quantity sufficient to prepare 120ml of solution.
    ii. The oral dose was 100mg/kg bodyweight, twice daily for up to 5 days.
    iii. The oral suspension was prepared by filling the bottle with water to the level marked with an arrow and shaking it well before use.
    iv. Oral fosfomycin was administered via syringe, spoon or nasogastric tube.
    v. Once reconstituted the bottle would be stored under temperature monitoring conditions between 2 and 8°C. Each bottle was dispensed to a single participant and used for 24 hours only (2 doses).

Clinical Definitions

Hypoglycaemia was defined as a random blood sugar ≤2.6 mmom/L as measured using ILab Aries (Instrumentation Laboratory, USA) or Accu-Check bedside glucometer (Roche, USA). Thrombocytopenia was defined as a platelet count <150×10^3/µL, and hypothermia as axillary body temperature <35.5℃. Oxygen saturation <90% in room air or while receiving oxygen support was considered as hypoxia. A participant was in respiratory distress if observed to have at least two of the following: tachypnoea (respiratory rate ≥60 breaths/minute), grunting, and chest wall indrawing. Diarrhoea was defined as presence of at least three loose motions in 24 hours, and a participant would be in shock if they presented with cool peripheries, a rapid and weak pulse, and delayed capillary refill time >2 seconds. Definite meningitis was defined as: i) positive cerebrospinal (CSF) culture; or ii) positive CSF latex agglutination test; or iii) positive CSF Gram stain microscopy; or iv) CSF leukocyte count ≥20 cells/µL plus positive blood culture for known pathogen. Possible meningitis was defined as CSF leukocyte count ≥20 cells/µL and negative blood culture. Clinically suspected sepsis was based on clinician's judgement at admission following WHO and Kenya national guidelines. Culture-confirmed sepsis was defined as a positive blood culture in the presence of clinical features suggestive of sepsis.

Safety Assessment and Follow up

All solicited and unsolicited AEs were evaluated for severity, seriousness and causality with each study treatment. Abnormal laboratory parameters were reported if found to be clinically significant e.g. suggesting a new or worsening pre-existing disease and/or organ toxicity, leading to discontinuation of medication, or requiring medical intervention. AEs were prospectively recorded and coded according to the Medical Dictionary for Regulatory Activities (MedDRA) version 22.0. Severity was classified according to Division of AIDS Table for Grading of Adult and Paediatric Adverse Events (DAIDS) version 2.1. AE grading not described on the DAIDS scale was done as follows: mild (does not interfere with participant's usual functions), moderate (interferes to some extent with usual functions), severe (interferes significantly with usual functions), life-threatening (participant is at risk of death at the time of the AE), or death.

Day 28 visit was conducted at the study clinic for most participants. Non-attenders were contacted by phone and/or home tracing. Families were invited to make unscheduled visits in case of illness after hospital discharge up to day 28.

PK Sample Processing and Measurement

Plasma and cerebrospinal fluid (CSF) samples were centrifuged at 3,000RPM (1351 RCF) for 5 minutes then separated and frozen at -80°C within 30 minutes of collection. Samples were shipped to Analytical Services International Ltd., St. Georges University of London, UK. Fosfomycin concentration in plasma and CSF samples was assessed via an in-house validated Liquid Chromatography Tandem Mass Spectrometry (LC-MS/MS) assay, including for frozen storage stability. The lower limit of quantification for plasma and CSF was 5mg/L and 1mg/L respectively. Assay methodology and fosfomycin stability data are described elsewhere.[8]

Supplementary Figures

Figure S1: Outline of study procedures

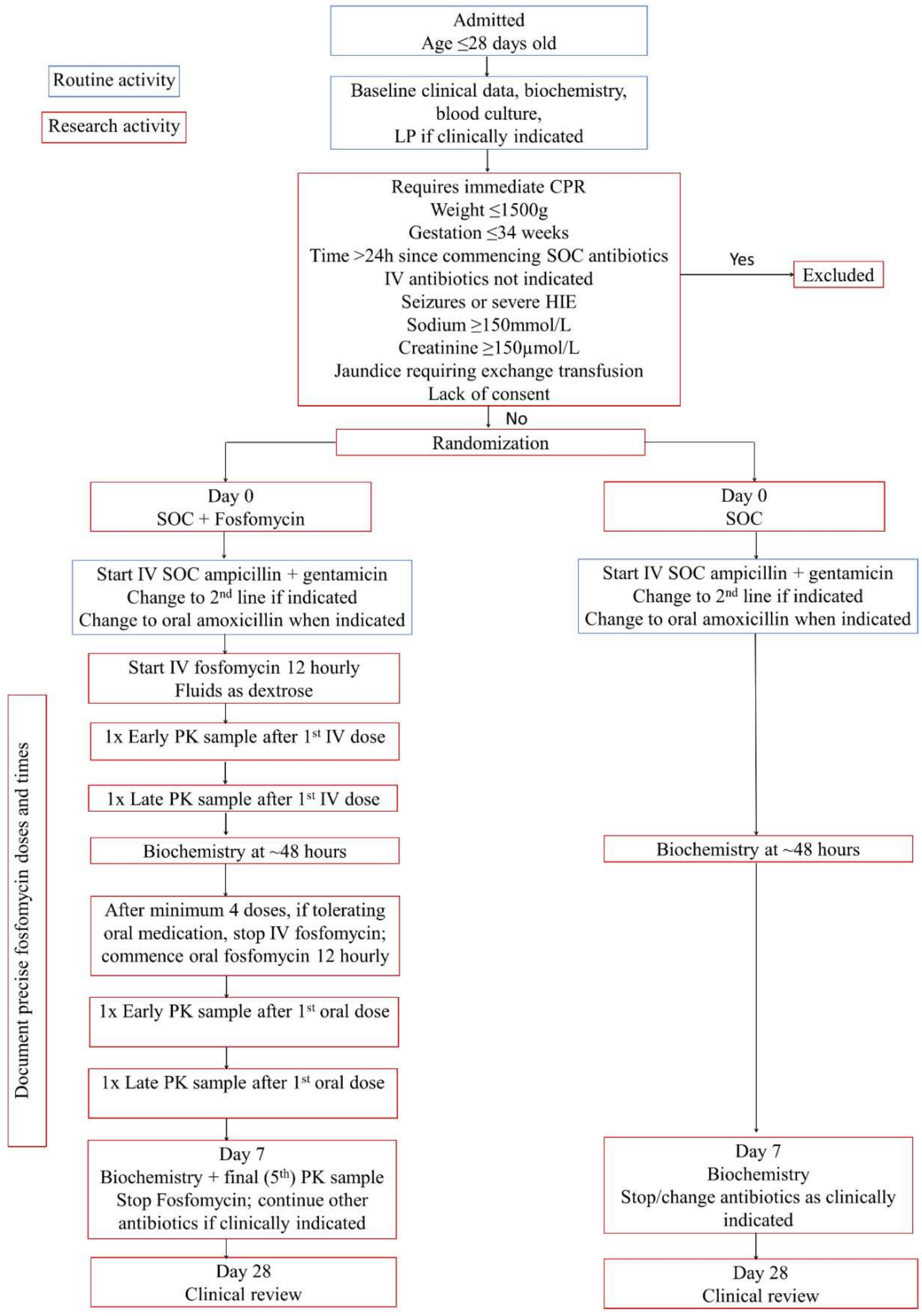

Abbreviations: LP, lumbar puncture; CPR, cardiopulmonary resuscitation; HIE, hypoxic ischaemic encephalopathy; SOC, standard of care; IV, intravenous; PK, pharmacokinetic.

Obiero CW, *et al. Arch Dis Child* 2022; 107:802–810. doi: 10.1136/archdischild-2021-322483

Figure S2. Plasma concentration-time curves for A: IV and B: oral fosfomycin administered at 100mg/kg/dose twice daily.

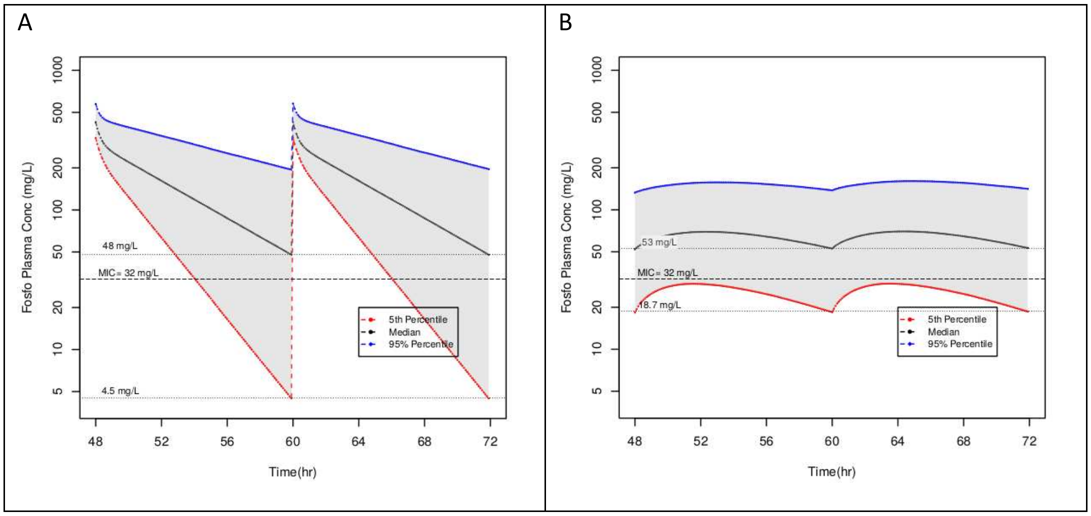

Obiero CW, *et al. Arch Dis Child* 2022; 107:802–810. doi: 10.1136/archdischild-2021-322483

Supplementary Tables

Table S1: Baseline laboratory test parameters by treatment arm

Parameter	SOC (n=59)	SOC-F (n=61)	Overall (n=120)
Haematology			
Haemoglobin (g/dL)	16 (2.9)	16 (2.3)	16 (2.6)
MCV (fl)	102 (9.0)	101 (8.0)	101 (8.5)
WBC ($x10^3/\mu L$)	16 (7.7)	16 (7.9)	16 (7.7)
Neutrophils ($x10^3/\mu L$)	7.5 (4.3)	7.6 (5.2)	7.6 (4.8)
Lymphocytes ($x10^3/\mu L$)	5.9 (3.6)	5.5 (4.3)	5.7 (4.0)
Monocytes ($x10^3/\mu L$)	1.3 (0.9)	1.4 (1.0)	1.4 (0.9)
Eosinophils ($x10^3/\mu L$)	0.3 (0.2)	0.3 (0.3)	0.3 (0.2)
Basophils ($x10^3/\mu L$)	1.1 (1.5)	1.0 (1.0)	1.1 (1.2)
Platelets ($x10^3/\mu L$)	248 (118)	268 (143)	258 (132)
Chemistry			
Creatinine (µmol/L)	92 (28)	89 (24)	90 (26)
Sodium (mmol/L)	135 (4.1)	136 (5.3)	136 (4.7)
Potassium (mmol/L)	4.3 (0.6)	4.3 (0.7)	4.3 (0.7)
AST (U/L)	91 (58)	82 (47)	86 (52)
ALT (U/L)	38 (35)	28 (20)	32 (28)
ALP (µmol/L)	225 (113)	224 (83)	225 (97)
Albumin (g/dL)	37 (4.6)	35 (5.0)	36 (4.9)
Total bilirubin (µmol/L)	58 (57)	78 (79)	68 (70)
Calcium (mmol/L)	2.3 (0.2)	2.3 (0.2)	2.3 (0.2)
Magnesium (mmol/L)	3.0 (13)	0.8 (0.2)	0.8 (8.9)
Phosphate (mmol/L)	1.9 (0.5)	2.1 (0.8)	2.0 (0.7)
Glucose (mmol/L)	3.7 (2.1)	3.4 (1.5)	3.6 (1.8)
Urea (mmol/L)	4.0 (3.4)	3.8 (2.1)	3.9 (2.8)
GGT (U/L)	119 (89)	116 (80)	117 (84)

Data are mean (sd).

Abbreviations: g/dL, gram/decilitre; MCV, mean corpuscular volume; fl, femtoliters; WBC, white blood cell; µL, microliter; µmol/L, micromole/litre; mmol/L, mmol/litre; U/L, units/litre; AST, aspartate aminotransferase; ALT, alanine transaminase; ALP, alkaline phosphatase; GGT, gamma glutamyl transferase.

Missing parameters: i) All haematological parameters (2 SOC and 1 SOC-F [sample obtained but clotted]) ii) Chemistry parameters (insufficient samples due to low sampling volume or haemoconcentration): Creatinine (1 SOC), ALT (22 SOC, 15 SOC-F), AST (21 SOC, 14 SOC-F), ALP (21 SCO, 13 SOC-F), Albumin (20 SOC, 10 SOC-F), total bilirubin (1 SOC-F), calcium (23 SOC, 12 SOC-F), magnesium (22 SOC, 18 SOC-F), phosphate (22 SOC, 15 SOC-F), glucose (3 SOC, 4 SOC-F), urea (23 SOC, 19 SOC-F), GGT (24 SOC, 16 SOC-F).

1 SOC participant missed serum creatinine at enrolment resulting in a protocol deviation. However, serum creatinine was 107 µmol/L a few hours after enrolment, 101 µmol/L at 48 hours, and 78 µmol/L prior to discharge.

Obiero CW, *et al. Arch Dis Child* 2022; 107:802–810. doi: 10.1136/archdischild-2021-322483

Table S2: Blood and CSF culture results by treatment arm

	SOC (n=59)		SOC-F (n=61)	
Isolate	CSF culture	Blood culture	CSF culture	Blood culture
Pathogen				
Acinetobacter spp.	0	1[a]	0	0
Streptococcus Group B	0	0	0	1[b]
Presumed non-significant				
Bacillus spp.	1	0	0	1
Corynebacterium spp.	0	2	0	0
L. adecarboxylata	0	0	0	1
S. epidermidis	0	0	0	1
S. haemolyticus	0	5	0	2
S. hominis	0	3	0	4

Data are n

[a]*Acinetobacter* spp. susceptible to gentamicin;

[b]*Streptococcus* Group B susceptible to penicillin.

Obiero CW, *et al. Arch Dis Child* 2022; 107:802–810. doi: 10.1136/archdischild-2021-322483

Table S3: Standard-of-care (SOC) antibiotic changes and day 2 sample collection

No.	1st line antibiotics	Date started	Date switched (specify)	Indication for antibiotics/change	Date of day 2 samples	Date IV SOC treatment stopped	Date fosfomycin stopped
SOC 1	Amp + gent	07May18	08May18 (clox + gent)	Septic skin lesions	09May18	13May18	N/A
SOC 2	Amp + gent	07Jul18	08Jul18 (ceftriaxone)	Meningitis	09Jul18	21Jul18	N/A
SOC 3	Amp + gent	23Jul18	N/A	Skin infection	25Jul18	26Jul18	N/A
SOC 4	Amp + gent	24Jul18	24Jul18 (ceftriaxone)	Clinical deterioration	26Jul18	02Aug18	N/A
SOC 5	Ceftriaxone	24Aug18	N/A	Suspected meningitis	26Aug18	27Aug18	N/A
SOC 6	Amp + gent	24Sep18	27Sep18 (ceftriaxone)	Clinical deterioration	27Sep18	03Oct18	N/A
SOC 7	Amp + gent	05Oct18	07Oct18 (ceftriaxone)	Possible meningitis	07Oct18	18Oct18	N/A
SOC 8	Amp + gent	06Oct18	14Oct18 (ceftriaxone)	Clinical deterioration	08Oct18	20Oct18	N/A
SOC 9	Amp + gent	01Nov18	02Nov18 (ceftriaxone)	Meningitis	04Nov18	15Nov18	N/A
SOC 10	Amp + gent	01Nov18	02Nov18 (ceftriaxone)	Suspected meningitis	04Nov18	14Nov18	N/A
SOC 11	Amp + gent	03Nov18	09Nov18 (ceftriaxone)	Clinical deterioration	06Nov18	13Nov18	N/A
SOC 12	Amp + gent	07Nov18	09Nov18 (ceftriaxone)	Clinical deterioration	09Nov18	13Nov18	N/A
SOC 13	Amp + gent	08Nov18	15Nov18 (ceftriaxone)	Clinical deterioration	11Nov18	19Nov18	N/A
SOC 14	Amp + gent	23Nov18	27Nov18 (ceftriaxone)	Bacteraemia	26Nov18	02Dec18	N/A
SOC 15	Clox + gent	08Jan19	N/A	Skin infection	10Jan19	11Jan19	N/A
SOC-F 1	Amp + gent	03May18	03May18 (ceftriaxone)	Bacteraemia, meningitis	05May18	16May18	09May18
SOC-F 2	Amp + gent	06Jun18	07Jun18 (ceftriaxone)	Clinical deterioration	08Jun18	11Jun18	12Jun18
SOC-F 3	Amp + gent	24Jul18	25Jul18 (ceftriaxone)	Clinical deterioration	NA (died)	25Jul18	25Jul18
SOC-F 4	Amp + gent	13Aug18	17Aug18 (ceftriaxone)	Possible meningitis	15Aug18	23Aug18	19Aug18
SOC-F 5	Amp + gent	29Oct18	01Nov18 (ceftriaxone)	Clinical deterioration	31Oct18	02Nov18	03Nov18
SOC-F 6	Amp + gent	29Oct18	29Oct18 (cefotaxime)	Clinical deterioration, hyperbilirubinemia	31Oct18	01Nov18	01Nov18
SOC-F 7	Amp + gent	02Nov18	05Nov18 (ceftriaxone)	Meningitis	04Nov18	16Nov18	09Nov18
SOC-F 8	Amp + gent	06Nov18	09Nov18 (ceftriaxone)	Clinical deterioration	08Nov18	13Nov18	09Nov18
SOC-F 9	Amp + gent	24Nov18	25Nov18 (ceftriaxone)	Clinical deterioration	27Nov18	29Nov18	29Nov18
SOC-F 10	Amp + gent	03Dec18	04Dec18 (ceftriaxone)	Clinical deterioration	06Dec18	06Dec18	07Dec18

Abbreviations: SOC, Standard-of-care; SOC-F, Standard-of-care plus fosfomycin; amp, ampicillin; gent, gentamicin; clox, cloxacillin; N/A, not applicable.

Table S4: Serious and non-serious AEs by treatment arm, severity and MedDRA coding classification

	SOC (n=34)						SOC-F (n=25)					
**DAIDS Grade**	**Grade 1**	**Grade 2**	**Grade 3**	**Grade 4**	**Grade 5**	**Total**	**Grade 1**	**Grade 2**	**Grade 3**	**Grade 4**	**Grade 5**	**Total**
Blood and lymphatic system disorders												
Anaemia	1	1	0	1	0	3	0	1	0	0	0	1
Neutropenia	0	0	1	0	0	1	0	0	0	0	0	0
Thrombocytopenia	0	2	2	0	0	4	0	2	1	0	0	3
Cardiac disorders												
Bradycardia	2	0	0	0	0	2	2	0	0	0	0	2
Congenital, familial and genetic												
Atrial septal defect	0	0	0	0	0	0	0	1	1[a]	0	0	2
Congenital intestinal malformation, aggravated	0	0	0	0	1[a]	1	0	0	0	0	0	0
Patent ductus arteriosus	0	0	0	0	0	0	0	1	0	0	0	1
Tetralogy of Fallot, aggravated	0	0	0	0	0	0	0	0	0	0	2[a]	2
Eye disorders												
Conjunctivitis	1	0	0	0	0	1	1	0	0	0	0	1
Eye discharge	1	0	0	0	0	1	0	0	0	0	0	0
Gastrointestinal disorders												
Abdominal distension	0	1	0	0	0	1	0	0	0	0	0	0

Obiero CW, *et al. Arch Dis Child* 2022; 107:802–810. doi: 10.1136/archdischild-2021-322483

	SOC (n=34)						SOC-F (n=25)					
**DAIDS Grade**	**Grade 1**	**Grade 2**	**Grade 3**	**Grade 4**	**Grade 5**	**Total**	**Grade 1**	**Grade 2**	**Grade 3**	**Grade 4**	**Grade 5**	**Total**
Diarrhoea	0	0	0	0	0	0	0	1	0	0	0	1
Vomiting	0	0	0	0	0	0	1	0	0	0	0	1
General disorders and administrative site conditions												
Fever neonatal	0	1[b]	0	0	0	1	0	0	0	0	0	0
Hypothermia	4	0	0	0	0	4	2	0	0	0	0	2
Oedema	0	1	0	0	0	1	0	0	0	0	0	0
Hepatobiliary disorders												
Jaundice	2	0	0	0	0	2	0	0	0	0	0	0
Infections and infestations												
Acrodermatitis	1	0	0	0	0	1	1	0	0	0	0	1
Lower respiratory tract infection	0	1	0	0	0	1	0	0	0	0	0	0
Meningitis neonatal	0	0	1	0	0	1	0	0	0	0	0	0
Neonatal sepsis, aggravated	0	0	0	0	2	2	0	0	0	0	1[a]	1
Pneumonia	0	1[b]	1[b]	0	0	2	0	0	0	0	0	0
Skin infection	2	0	0	0	0	2	0	0	0	0	0	0
Upper respiratory tract infection	1	0	0	0	0	1	1	0	0	0	0	1

	SOC (n=34)						SOC-F (n=25)					
**DAIDS Grade**	**Grade 1**	**Grade 2**	**Grade 3**	**Grade 4**	**Grade 5**	**Total**	**Grade 1**	**Grade 2**	**Grade 3**	**Grade 4**	**Grade 5**	**Total**
Investigations												
Hepatic enzyme increased	0	1	1	0	0	2	0	0	0	0	0	0
Metabolism and nutrition disorders												
Failure to thrive	1	0	0	0	0	1	0	0	0	0	0	0
Hypoglycaemia neonatal	2	0	2	2	0	6	0	1	2	2	0	5
Hypokalaemia	2	0	0	0	0	2	1	2	0	0	0	3
Nervous system disorders												
Neonatal seizure	0	0	1	0	1	2	0	1	2	0	0	3
Reproductive system and breast disorders												
Vaginal haemorrhage	1	0	0	0	0	1	0	0	0	0	0	0
Respiratory, thoracic and mediastinal disorders												
Infantile apnoea	0	0	0	1	1[a]	2	0	0	0	0	0	0
Neonatal asphyxia	0	0	0	0	0	0	0	0	1	0	1[a]	2
Skin and subcutaneous tissue disorders												
Dermatitis, diaper	0	0	0	0	0	0	1	0	0	0	0	1
Rash	1	0	0	0	0	1	2	0	0	0	0	2
Skin lesion	1	0	0	0	0	1	0	0	0	0	0	0

Obiero CW, *et al. Arch Dis Child* 2022; 107:802–810. doi: 10.1136/archdischild-2021-322483

	SOC (n=34)						SOC-F (n=25)					
**DAIDS Grade**	**Grade 1**	**Grade 2**	**Grade 3**	**Grade 4**	**Grade 5**	**Total**	**Grade 1**	**Grade 2**	**Grade 3**	**Grade 4**	**Grade 5**	**Total**
Total	23	9	9	4	5	50	12	10	7	2	4	35

Data are n

[a]Worsening of pre-existing conditions and those resulting in death included: Tetralogy of Fallot (2 SOC-F); congenital intestinal malformation (1 SOC); birth asphyxia (1 SOC-F); worsening neonatal sepsis together with atrial septal defect (1 SOC-F); birth asphyxia and infantile apnoea (1 SOC)

[b]Three SOC participants were re-admitted to hospital and discharged home alive (pneumonia [n=2] and febrile illness of unknown origin [n=1])

Obiero CW, *et al. Arch Dis Child* 2022; 107:802–810. doi: 10.1136/archdischild-2021-322483

Table S5. Anticipated adverse events by treatment arm

	**SOC (n=59)**	**SOC-F (n=61)**	**All (n=120)**
Anaemia	2	0	2
Atrial Septal Defect	0	1	1
Congenital intestinal malformation	1	0	1
Hypoglycaemia	6	4	10
Infantile apnoea	1	0	1
Jaundice	2	0	2
Meningitis neonatal	1	0	1
Neonatal asphyxia	0	2	2
Neonatal seizure	2	3	5
Neonatal sepsis	1	1	2
Patent Ductus Arteriosus	0	1	1
Tetralogy of Fallot	0	2	2
Total	16	14	30
Data are n			

Obiero CW, *et al. Arch Dis Child* 2022; 107:802–810. doi: 10.1136/archdischild-2021-322483

Table S6: AE outcome at study termination excluding events that either resolved or occurred in neonates who died

**Outcome**	**SOC (n=17)**	**SOC-F (n=10)**
Resolving (n=5)	Anaemia (1)	Acrodermatitis (1)
	Conjunctivitis (1)	Rash (1)
	Eye discharge (1)	
Not resolved (n=20)	Anaemia (1)	Atrial septal defect (2)
	Acrodermatitis (1)	Hypokalaemia (1)
	Failure to thrive (1)	Patent ductus arteriosus (1)
	Hepatic enzyme increased (2)	Thrombocytopenia (3)
	Neutropenia (1)	
	Oedema (1)	
	Rash (1)	
	Skin lesion (1)	
	Skin infection (2)	
	Thrombocytopenia (2)	
Resolved with sequelae (n=2)	Thrombocytopenia (1)	Hypokalaemia (1)
Data are n		

Table S7 Simulated Steady State PK Summary – Sub-populations

		AUC48-72 (hr*mg/L)			Cmin (mg/L)			Cmax (mg/L)			T>MIC (hr)		
Regimen	Group	P5	P50	P95	P5	P50	P95	P5	P50	P95	P5	P50	P95
100_IV	1	2962.5	5554.6	10019.0	34.2	126.6	304.9	379.3	497.8	687.7	24.0	24.0	24.0
150_IV	1	4443.8	8331.9	15028.4	51.2	189.9	457.3	568.9	746.6	1031.6	24.0	24.0	24.0
200_IV	1	5925.0	11109.3	20037.9	68.32	253.2	609.7	758.6	995.5	1375.4	24.0	24.0	24.0
100_PO	1	1115.2	2542.9	4968.8	38.8	95.8	200.5	51.0	113.3	218.6	24.0	24.0	24.0
200_PO	1	2230.3	5085.8	9937.6	77.7	191.5	401.1	102.0	226.6	437.2	24.0	24.0	24.0
300_PO	1	3345.5	7628.7	14906.4	116.5	287.3	601.6	153.0	340.0	655.7	24.0	24.0	24.0
100_IV	2	1256.5	2086.9	3606.5	1.8	13.8	56.9	308.6	385.2	478.8	9.8	17.0	24.0
150_IV	2	1884.7	3130.3	5409.7	2.7	20.7	85.4	462.9	577.8	718.2	11.8	20.4	24.0
200_IV	2	2512.9	4173.8	7212.9	3.5	27.5	113.9	617.2	770.4	957.5	13.2	22.6	24.0
100_PO	2	464.5	959.9	1890.0	13.4	30.4	66.3	23.0	46.1	86.0	0.0	22.1	24.0
200_PO	2	929.1	1919.9	3779.9	26.8	60.8	132.5	45.9	92.2	172.0	19.0	24.0	24.0
300_PO	2	1393.6	2879.8	5669.9	40.1	91.2	198.8	68.9	138.3	258.0	24.0	24.0	24.0
100_IV	3	1659.3	3056.4	5508.1	6.4	38.3	120.6	325.2	413.4	517.4	13.4	24.0	24.0
150_IV	3	2488.9	4584.6	8262.2	9.5	57.4	180.8	487.8	620.1	776.1	16.2	24.0	24.0
200_IV	3	3318.5	6112.8	11016.2	12.7	76.5	241.1	650.4	826.8	1034.8	18.0	24.0	24.0
100_PO	3	646.3	1392.7	2817.2	20.2	47.7	104.9	30.8	64.4	125.7	0.0	24.0	24.0
200_PO	3	1292.6	2785.5	5634.4	40.4	95.5	209.7	61.6	128.7	251.4	24.0	24.0	24.0
300_PO	3	1938.9	4178.2	8451.6	60.6	143.2	314.6	92.4	193.1	377.0	24.0	24.0	24.0
100_IV	4	2271.0	3883.8	6805.8	17.5	63.6	173.0	345.9	438.5	567.4	18.6	24.0	24.0
150_IV	4	3406.5	5825.7	10208.7	26.2	95.4	259.6	518.8	657.7	851.1	22.2	24.0	24.0
200_IV	4	4541.9	7767.6	13611.6	34.9	127.3	346.1	691.7	877.0	1134.9	24.0	24.0	24.0
100_PO	4	860.8	1792.9	3500.4	28.5	63.9	132.7	40.2	80.9	154.8	19.1	24.0	24.0
200_PO	4	1721.7	3585.8	7000.8	57.0	127.8	265.5	80.5	161.8	309.6	24.0	24.0	24.0
300_PO	4	2582.5	5378.7	10501.2	85.5	191.7	398.2	120.7	242.6	464.4	24.0	24.0	24.0

Abbreviations: AUC, area under concentration-time curve; C, plasma concentration; min, minimum; max, maximum; T>MIC, fraction of time plasma concentrations exceeds the MIC (minimum inhibitory concentration); mg, milligram; L, litre; hr, hour; P5, 5th percentile; P50, median; P95, 95th percentile; IV, intravenous; PO, oral; WT, weight; PNA, postnatal age.
T>MIC assumes MIC=32mg/L
Group 1: WT >1.5kg + PNA ≤7days
Group 2: WT >1.5kg + PNA >7days
Group 3: WT ≤1.5kg + PNA ≤7days
Group 4: WT ≤1.5kg + PNA >7days

Obiero CW, *et al. Arch Dis Child* 2022; 107:802–810. doi: 10.1136/archdischild-2021-322483

References

1. MOH. Basic Paediatric Protocols for ages up to 5 years. Fourth ed, February 2016.
2. Ballard JL, Khoury JC, Wedig K, et al. New Ballard Score, expanded to include extremely premature infants. *The Journal of pediatrics* 1991;119(3):417-23. doi: 10.1016/s0022-3476(05)82056-6 [published Online First: 1991/09/01]
3. Schulga P, Grattan R, Napier C, et al. How to use... lumbar puncture in children. *Arch Dis Child Educ Pract Ed* 2015;100(5):264-71. doi: 10.1136/archdischild-2014-307600 [published Online First: 2015/06/25]
4. Berkley JA, Lowe BS, Mwangi I, et al. Bacteremia among children admitted to a rural hospital in Kenya. *The New England journal of medicine* 2005;352(1):39-47. doi: 10.1056/NEJMoa040275 [published Online First: 2005/01/07]
5. Berkley JA, Mwangi I, Mellington F, et al. Cerebral malaria versus bacterial meningitis in children with impaired consciousness. *QJM : monthly journal of the Association of Physicians* 1999;92(3):151-7. [published Online First: 1999/05/18]
6. Mwaniki MK, Talbert AW, Njuguna P, et al. Clinical indicators of bacterial meningitis among neonates and young infants in rural Kenya. *BMC infectious diseases* 2011;11:301. doi: 10.1186/1471-2334-11-301 [published Online First: 2011/11/03]
7. WHO. Pocket book of hospital care for children: Guidelines for the management of common childhood illnesses. Second ed: World Health Organization, 2013:412.
8. Kane Z, Gastine S, Obiero C, et al. IV and oral fosfomycin pharmacokinetics in neonates with suspected clinical sepsis. *The Journal of antimicrobial chemotherapy* 2021;76(7):1855-64. doi: 10.1093/jac/dkab083 [published Online First: 2021/04/16]
